# Chemical Synergy between Ionophore PBT2 and Zinc Reverses Antibiotic Resistance

**DOI:** 10.1128/mBio.02391-18

**Published:** 2018-12-11

**Authors:** Lisa Bohlmann, David M. P. De Oliveira, Ibrahim M. El-Deeb, Erin B. Brazel, Nichaela Harbison-Price, Cheryl-lynn Y. Ong, Tania Rivera-Hernandez, Scott A. Ferguson, Amanda J. Cork, Minh-Duy Phan, Amelia T. Soderholm, Mark R. Davies, Graeme R. Nimmo, Gordon Dougan, Mark A. Schembri, Gregory M. Cook, Alastair G. McEwan, Mark von Itzstein, Christopher A. McDevitt, Mark J. Walker

**Affiliations:** a School of Chemistry and Molecular Biosciences and Australian Infectious Diseases Research Centre, The University of Queensland, Brisbane, QLD, Australia; b Institute for Glycomics, Griffith University, Brisbane, QLD, Australia; c Research Centre for Infectious Diseases, School of Biological Sciences, University of Adelaide, Adelaide, SA, Australia; d Department of Microbiology and Immunology, University of Otago, Dunedin, New Zealand; e Department of Microbiology and Immunology at the Peter Doherty Institute for Infection and Immunity, The University of Melbourne, Melbourne, VIC, Australia; f Pathology Queensland Central Laboratory, Brisbane, QLD, Australia; g Wellcome Trust Sanger Institute, Hinxton, United Kingdom; Nanyang Technological University; Emory University School of Medicine; University Hospital Zurich, University of Zurich

**Keywords:** *Enterococcus faecium*, *Staphylococcus aureus*, *Streptococcus pyogenes*, antibiotic resistance

## Abstract

The World Health Organization reports that antibiotic-resistant pathogens represent an imminent global health disaster for the 21st century. Gram-positive superbugs threaten to breach last-line antibiotic treatment, and the pharmaceutical industry antibiotic development pipeline is waning. Here we report the synergy between ionophore-induced physiological stress in Gram-positive bacteria and antibiotic treatment. PBT2 is a safe-for-human-use zinc ionophore that has progressed to phase 2 clinical trials for Alzheimer’s and Huntington’s disease treatment. In combination with zinc, PBT2 exhibits antibacterial activity and disrupts cellular homeostasis in erythromycin-resistant group A *Streptococcus* (GAS), methicillin-resistant Staphylococcus aureus (MRSA), and vancomycin-resistant *Enterococcus* (VRE). We were unable to select for mutants resistant to PBT2-zinc treatment. While ineffective alone against resistant bacteria, several clinically relevant antibiotics act synergistically with PBT2-zinc to enhance killing of these Gram-positive pathogens. These data represent a new paradigm whereby disruption of bacterial metal homeostasis reverses antibiotic-resistant phenotypes in a number of priority human bacterial pathogens.

## Abstract (precis)

The rise of bacterial antibiotic resistance coupled with a reduction in new antibiotic development has placed significant burdens on global health care. Resistant bacterial pathogens such as methicillin-resistant Staphylococcus aureus and vancomycin-resistant *Enterococcus* are leading causes of community- and hospital-acquired infection and present a significant clinical challenge. These pathogens have acquired resistance to broad classes of antimicrobials. Furthermore, Streptococcus pyogenes, a significant disease agent among Indigenous Australians, has now acquired resistance to several antibiotic classes. With a rise in antibiotic resistance and reduction in new antibiotic discovery, it is imperative to investigate alternative therapeutic regimens that complement the use of current antibiotic treatment strategies. As stated by the WHO Director-General, “On current trends, common diseases may become untreatable. Doctors facing patients will have to say, Sorry, there is nothing I can do for you.”

## INTRODUCTION

Combating antibiotic resistance remains a critical global health priority (1). Over the past decade, the overall trend of bacterial pathogens exhibiting drug resistance has steadily increased (1–3). In high-income settings such as the United States, reports estimate that more than 2 million antibiotic-resistant infections occur per annum with a death toll of 23,000 at a direct cost of $20 billion (3). In low-income settings, communicable diseases remain the leading cause of death, now heightened by emerging and reemerging infectious diseases (4). Largely driven by the excessive and inappropriate use of antibiotics, the threat of antibiotic resistance has recently been amplified by a substantial decline in new antimicrobial discovery. From 2011 to 2016, only 8 new antibiotics were approved by the U.S. FDA (5). This significant decline in novel antibiotic discovery and commercialization has paralleled an escalation in antibiotic resistance, highlighting the urgent need for new antibiotic development and complementary therapy. Today, several strategies are being investigated to combat bacterial resistance to existing antibiotics (5–7), including the repurposing of existing drugs, originally developed as therapeutics for noninfectious disease.

The hydroxyquinoline PBT2 has been developed as a potential treatment for Alzheimer’s and Huntington’s disease and has progressed to phase 2 human clinical trials, with a daily dose of 250 mg over 6 months being safe and well tolerated (8–10). PBT2 is an ionophore that facilitates the transport of first-row transition metal ions, such as zinc, across biological membranes, thereby altering intracellular metal homeostasis. Bacterial pathogens encounter significant fluctuations in metal ion abundance during host colonization and have been shown to be highly susceptible to phagocytic cell induction of zinc toxicity (11). Although metals such as zinc are critical nutrients for bacterial cell survival, metal-transporting ionophores can facilitate accumulation of high levels of zinc in the bacterial cytosol, leading to bacterial cell toxicity (11, 12). Here, we report that in combination with zinc, the ionophore PBT2 destabilizes key cellular homeostasis pathways involved in bacterial antibiotic resistance mechanisms. Furthermore, the combination of PBT2 and zinc is able to resensitize erythromycin-resistant group A *Streptococcus* (GAS), methicillin-resistant Staphylococcus aureus (MRSA), and vancomycin-resistant *Enterococcus* (VRE) to previously resistant classes of antibiotic *in vivo*. These data highlight a new and encouraging alternative therapeutic regimen which complements the use of current antibiotic strategies.

## RESULTS AND DISCUSSION

PBT2 was originally developed as a potential therapeutic for Alzheimer’s and Huntington’s disease. Here, we sought to examine the therapeutic potential of PBT2 as an antibacterial agent, which has not been previously tested. Following chemical synthesis of PBT2 (13) (see Materials and Methods and Fig. S1 in the supplemental material), the purity of the final product was determined to be >95% by ^1^H and ^13^C NMR.

10.1128/mBio.02391-18.1 FIG S1 ^1^H and ^13^C NMR spectra of PBT2. (a) ^1^H NMR spectrum of PBT2 (400 MHz, CDCl_3_). (b) ^13^C NMR spectrum of PBT2 (100 MHz, CDCl_3_). Download FIG S1, PDF file, 0.1 MB. Copyright © 2019 Bohlmann et al. 2019 Bohlmann et al. This content is distributed under the terms of the Creative Commons Attribution 4.0 International license.

**FIG 1:**
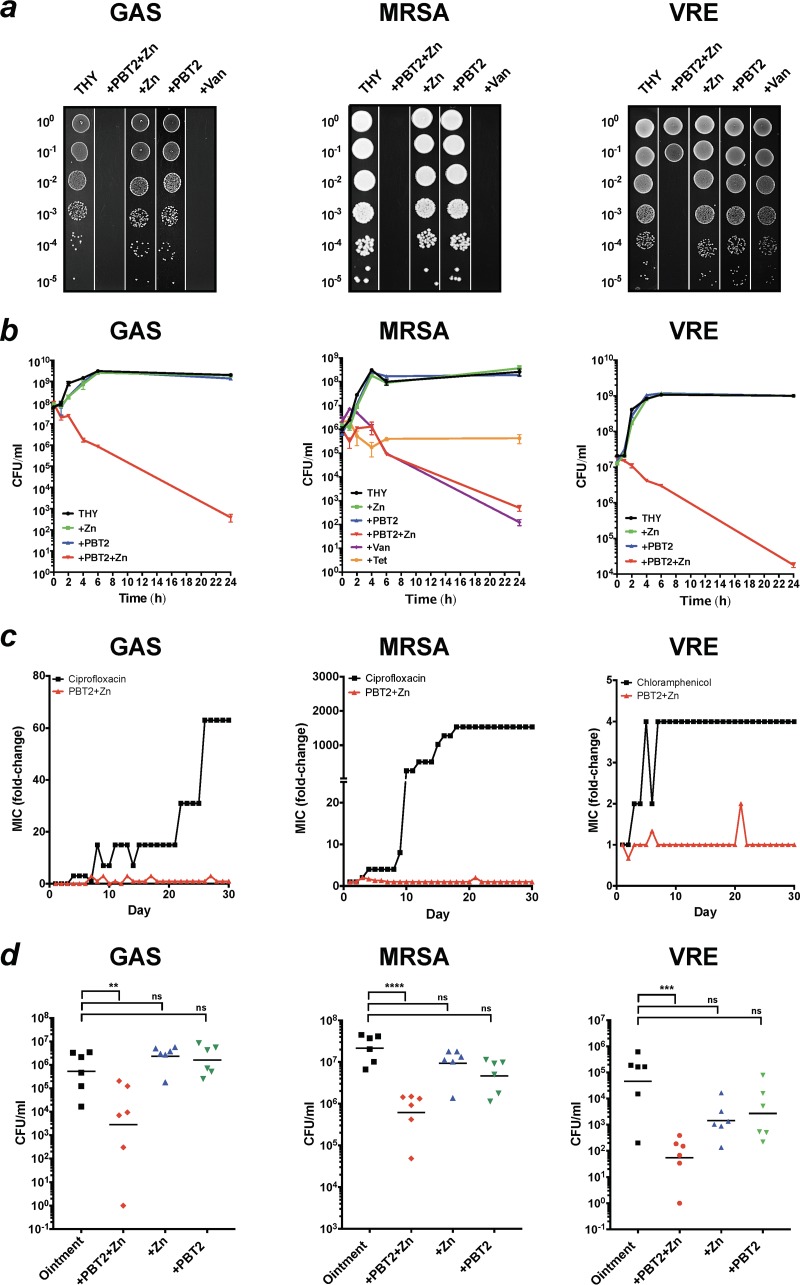
Synergistic antimicrobial activity of PBT2 and zinc. (a) Growth of GAS, MRSA, and VRE serially diluted on THY agar in the presence or absence of PBT2 (1.5 μM) and/or zinc (400 μM) or vancomycin (20 μg/ml). Dilution values are indicated on the left of each figure panel. (b) Time-kill curves of GAS, MRSA, and VRE in THY broth with or without PBT2 (2 μM for GAS or 6 μM for MRSA and VRE) and/or ZnSO_4_ (400 μM for GAS and 600 μM for MRSA and VRE) or vancomycin (bactericidal; 4 μg/ml for MRSA) or tetracycline (bacteriostatic; 1 μg/ml for MRSA). Error bars indicate standard deviations from 2 biological replicates. (c) Development of resistance during serial passage in the presence of subinhibitory concentrations of antimicrobial compounds in CAMHB. Data represent means for 3 biological replicates. (d) CFU recovered from a murine wound infection model 4 days after challenge with GAS (1.1 × 10^7^ CFU), MRSA (6 × 10^5^ CFU), or VRE (8.8 × 10^5^ CFU). Mice were treated twice daily with carrier ointment only or ointment with 5 mM PBT2 and/or 50 mM Zn (ZnSO_4_ for MRSA or ZnCl_2_ for GAS) or 2.75 mM PBT2 and/or 75 mM ZnSO_4_ for VRE. Values for individual mice are plotted, and black lines are representative of group geometric mean (*, *P* < 0.05; **, *P* < 0.005; ***, *P* < 0.001; ****, *P* < 0.0001, one-way ANOVA).

Erythromycin-resistant GAS, MRSA, and VRE are categorized by the U.S. Centers for Disease Control and Prevention as concerning or serious threats to human health (3). Using Clinical and Laboratory Standards Institute guidelines for antimicrobial sensitivity testing (14), we investigated the potential of PBT2 to act as an antibacterial agent against GAS strain HKU16 (15), MRSA strain USA300 (16), and VRE clinical isolate RBWH1 (Fig. S2). At the concentrations used, neither PBT2 nor zinc displayed significant antibacterial activity. However, the combination of PBT2 and zinc (PBT2-zinc) exhibited antibacterial activity against each pathogen (Fig. 1a and Table S1). Bactericidal activity is defined as a ≥3-log reduction in bacterial CFU relative to the initial inoculum. Measured to the effects of vancomycin (bactericidal) and tetracycline (bacteriostatic) on MRSA, the mode of action of PBT2-zinc was found to be bactericidal (Fig. 1b). We next investigated the capacity of each pathogen to develop resistance to the combination of PBT2-zinc. We were unable to isolate resistant mutants for any of the strains following serial passage for a period of 30 days in the presence of subinhibitory concentrations of PBT2-zinc (Fig. 1c). To explore the safety of PBT2-zinc in human cells, we investigated the survival of human primary tonsil epithelial cells in the presence of this combination. After 24 h, cell viability was unaffected by PBT2-zinc treatment (Fig. S3). We next investigated the efficacy of PBT2-zinc treatment using a murine wound infection model (17). The application of PBT2-zinc (zinc in the form of either ZnCl_2_ or ZnSO_4_) resulted in a significant reduction in the bacterial burden at the site of infection upon topical treatment (Fig. 1d).

10.1128/mBio.02391-18.2 FIG S2 Comparative analysis of Enterococcus faecium RBWH1 chromosome to published ST203 genome sequences. Relative position of blast hits of published Enterococcus faecium genomes 6E6 (K. Geldart and Y. N. Kaznessis, Antimicrob Agents Chemother 61:e02033-16, 2017, https://doi.org/10.1128/AAC.02033-16), Aus0085 (M. M. Lamm, T. Seemann, N. J. Tobias, H. Chen, et al., BMC Genomics 14:595, 2013, https://doi.org/10.1186/1471-2164-14-595), and VRE001 (E. S. Honsa, V. S. Cooper, M. N. Mhaissen, M. Frank, et al., mBio 8:e02124-16, 2017, https://doi.org/10.1128/mBio.02124-16) against the RBWH1 chromosome. Download FIG S2, PDF file, 1.1 MB. Copyright © 2019 Bohlmann et al. 2019 Bohlmann et al. This content is distributed under the terms of the Creative Commons Attribution 4.0 International license.

10.1128/mBio.02391-18.3 FIG S3 Cell death measured by percentage of LDH released from TEpi cells at 6 and 24 h. TEpi cells were incubated with increasing concentrations of PBT2-zinc for 24 h at 37°C, 5% CO_2_. Data are plotted as mean ± standard error of the mean and represent three independent experiments performed in triplicate and analyzed by two-way ANOVA with Tukey’s posttest. Download FIG S3, PDF file, 0.04 MB. Copyright © 2019 Bohlmann et al. 2019 Bohlmann et al. This content is distributed under the terms of the Creative Commons Attribution 4.0 International license.

10.1128/mBio.02391-18.7 TABLE S1 PBT2 and zinc are active against Gram-positive bacterial pathogens. Download Table S1, PDF file, 0.1 MB. Copyright © 2019 Bohlmann et al. 2019 Bohlmann et al. This content is distributed under the terms of the Creative Commons Attribution 4.0 International license.

**FIG 2:**
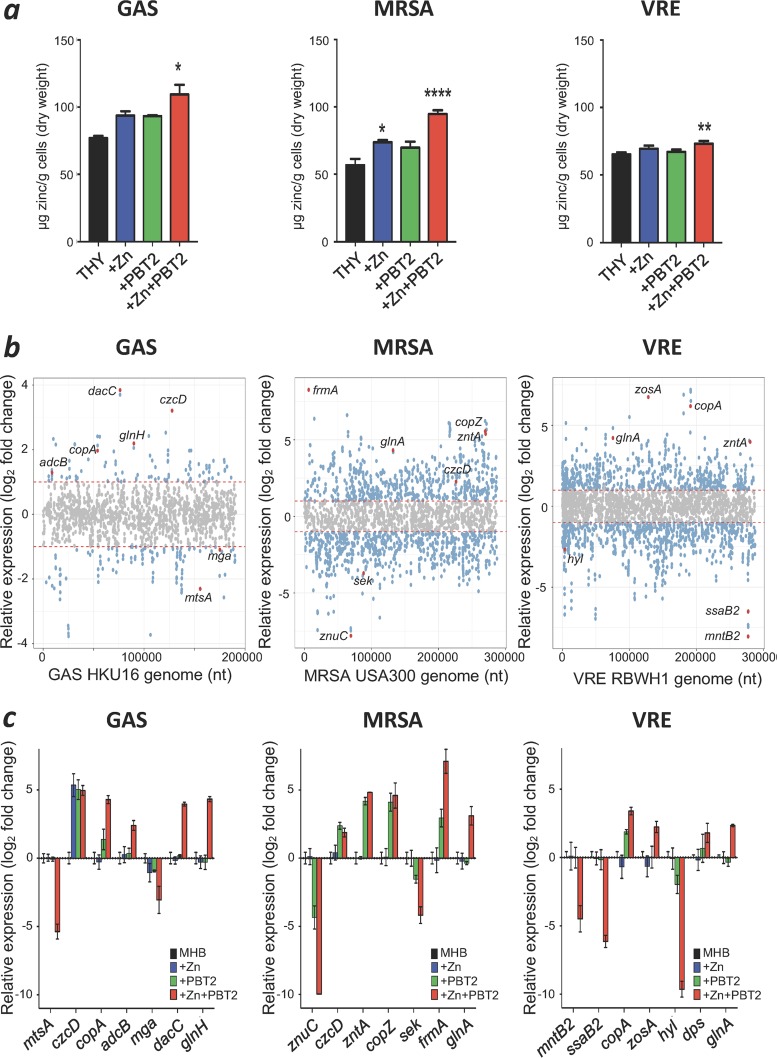
PBT2 and zinc affect heavy metal homeostasis, metabolism and virulence. (a) Intracellular zinc concentrations as determined by ICP-MS for GAS, MRSA, and VRE grown in THY with or without PBT2 and zinc (GAS, 0.3 μM PBT2 + 50 μM ZnSO_4_; MRSA, 1 μM PBT2 + 200 μM ZnSO_4_; and VRE, 1 μM PBT2 + 150 μM ZnSO_4_) (error bars indicate standard error of the mean from at least 3 biological replicates; *, *P* < 0.05; **, *P* < 0.005; ***, *P* < 0.001; ****, *P* < 0.0001, one-way ANOVA). (b) RNASeq transcriptome analysis of bacteria treated with PBT2 and ZnSO_4_ for GAS (4.75 μM PBT2 + 128 μM ZnSO_4_), MRSA (2 μM PBT2 + 50 μM ZnSO_4_), and VRE (1.75 μM PBT2 + 128 μM ZnSO_4_) in CAMHB. Genes with log_2_ fold changes of >1/<1 and *P* < 0.05 are shown in blue, and selected genes of interest are shown in red. Data were collected from 3 biological replicates. (c) Transcript levels for selected genes measured by real-time PCR. Log_2_ fold changes were calculated relative to untreated controls and normalized to a reference gene using the ΔΔ*C_T_* method (reference genes were *proS* for GAS, *rrsA* for MRSA, and 23S for VRE). Error bars represent standard deviations from 3 biological replicates.

Next, we determined the intracellular zinc content of each pathogen in response to subinhibitory concentrations of PBT2-zinc. As assessed by inductively coupled plasma mass spectrometry (ICP-MS), increased cellular levels of zinc were observed in GAS, MRSA, and VRE upon treatment (Fig. 2a). PBT2 and zinc combinations were also shown to affect intracellular iron, manganese, and copper content, suggesting a mechanism involving disruption of heavy metal homeostasis (Fig. S4A to C). To further explore the mechanism of action of PBT2-zinc treatment, we analyzed the transcriptome of each pathogen in response to subinhibitory concentrations of PBT2-zinc. Significant changes in the transcription of heavy metal homeostasis genes were observed and confirmed by quantitative real-time PCR (Fig. 2b and c; Table S2). Consistent with metal ion intoxication, the transcriptional responses to PBT2-zinc treatment in the Gram-positive pathogens included the induction of zinc efflux systems (GAS, *czcD*; MRSA, *czcD* and *zntA*), copper efflux systems (GAS, *copA*; MRSA, *copZ;* VRE, *copA*), and manganese transport systems (VRE, *mntA*). In addition, the transcription of several essential virulence, oxidative stress, and metabolic systems was also perturbed by subinhibitory concentrations of PBT2-zinc (Fig. 2b and c; Table S2). Despite these changes in the transcriptional profile, PBT2-zinc did not alter cell membrane permeability of GAS, MRSA, and VRE compared to the pore-forming antibiotic nisin (Fig. S5A). PBT2-zinc did not visibly disrupt the bacterial cell wall of GAS and MRSA (Fig. S5B and C); however, visible depressions in the VRE cell wall were observed after 3 h of treatment and VRE cells became enlarged after 24 h (Fig. S5D).

10.1128/mBio.02391-18.4 FIG S4 PBT2 and zinc affect heavy metal homeostasis. Intracellular zinc, iron, manganese, and copper concentrations as determined by ICP-MS for GAS (a), MRSA (b), and VRE (c). Bacteria were grown in THY with or without PBT2 and zinc (GAS, 0.3 μM PBT2 + 50 μM ZnSO_4_; MRSA, 1 μM PBT2 + 200 μM ZnSO_4_; and VRE, 1 μM PBT2 + 150 μM ZnSO_4_) (error bars indicate standard error of the mean from at least 3 biological replicates; *, *P* < 0.05; **, *P* < 0.005; ***, *P* < 0.001; ****, *P* < 0.0001, one-way ANOVA). Download FIG S4, PDF file, 0.1 MB. Copyright © 2019 Bohlmann et al. 2019 Bohlmann et al. This content is distributed under the terms of the Creative Commons Attribution 4.0 International license.

10.1128/mBio.02391-18.5 FIG S5 Effect of PBT2 and zinc on cell morphology. (a) Membrane permeability of GAS, MRSA, and VRE after treatment with PBT2, zinc, or nisin (25 μg/ml) for 1 h (GAS, 4.75 μM PBT2, 128 μM zinc; MRSA, 2 μM PBT2, 50 μM zinc; VRE, 1.75 μM PBT2, 128 μM zinc). Error bars indicate standard deviation from 3 biological replicates; ***, *P* < 0.001; ****, *P* < 0.0001; unpaired *t* test). (b to d) SEM images of GAS (b), MRSA (c), and VRE (d) untreated (control) or treated with 6 μM PBT2 and 500 μM zinc for 3 h or 24 h. Scale bar equals 1 μm; arrows indicate depressions (3 h) and enlarged cells (24 h). Download FIG S5, PDF file, 34.4 MB. Copyright © 2019 Bohlmann et al. 2019 Bohlmann et al. This content is distributed under the terms of the Creative Commons Attribution 4.0 International license.

10.1128/mBio.02391-18.8 TABLE S2 Differentially expressed genes after treatment with PBT2 and zinc. Download Table S2, PDF file, 1.2 MB. Copyright © 2019 Bohlmann et al. 2019 Bohlmann et al. This content is distributed under the terms of the Creative Commons Attribution 4.0 International license.

Building on the observed significant transcriptional and physiological impacts induced by the combination of PBT2-zinc upon these bacterial strains (Table 1), we then assessed whether these disruptions enhanced antibiotic sensitivity in otherwise resistant bacterial pathogens. To achieve this, the tetracycline- and macrolide-resistant GAS strain HKU16 and the multidrug-resistant strains MRSA USA300 and VRE RBWH1 were further investigated. In the presence of antibacterial only, HKU16, USA300, and RBWH1 were resistant to several antibiotic classes (Table 1). Addition of either PBT2 or zinc alone did not affect their susceptibility profile. However, in the presence of subinhibitory concentrations of PBT2-zinc, GAS strain HKU16 became susceptible to tetracycline, azithromycin, and clindamycin; MRSA strain USA300 became susceptible to oxacillin, erythromycin, and ampicillin; and VRE strain RBWH1 became susceptible to vancomycin, tetracycline, azithromycin, and clindamycin (Table 1). While resistance to erythromycin was overcome in MRSA USA300, erythromycin resistance was maintained in GAS HKU16 and VRE RBWH1. This difference may relate to the inducible erythromycin resistance mechanism in MRSA USA300 (clindamycin sensitive) compared to the alternative constitutive erythromycin resistance mechanism (clindamycin resistant) (15, 18–20) operating in GAS HKU16 and VRE RBWH1 (Table 1). Bactericidal activity of PBT2-zinc and antibiotic combinations was partially rescued with the addition of the antioxidant glutathione (Fig. S6), suggesting the involvement of reactive active oxygen species in the mechanism of cell killing (21).

**TABLE 1:** Combination of PBT2 and zinc resensitizes pathogenic Gram-positive bacteria to antibiotics of various classes

Antibiotic	MIC (μg/ml)^*a*^											
	GAS				MRSA^*c*^				VRE			
	0 μM PBT2- 0 μM Zn	4.75 μM PBT2- 128 μM Zn	4.75 μM PBT2	128 μM Zn	0 μM PBT2- 0 μM Zn	2 μM PBT2- 50 μM Zn	2 μM PBT2	50 μM Zn	0 μM PBT2- 0 μM Zn	1.75 μM PBT2- 128 μM Zn	1.75 μM PBT2	128 μM Zn
Erythromycin	>128	>128	>128	>128	64	**0.5**	32	64	>128	64	>128	>128
Azithromycin	>128	**1–2**	>128	>128	>128	16–32	128	>128	>128	**2**	>128	>128
Clindamycin	>128	**1**	>128	>128	0.5	0.25	0.25	0.5	>128	**2**	>128	>128
Tetracycline	64–128	**2–4**	64	128	0.5	<0.125	0.25	0.5	>128	**2–4**	128	>128
Ampicillin	0.5	0.25	0.25	0.5	>128	**2**	>128	>128	>128	64	>128	>128
Vancomycin	0.5	0.25	0.5	0.5	2	1	2	2	>128	**1–2**	>128	>128
Oxacillin^*b*^	0.5	0.25	0.5	0.5	128	**1–2**	128	128	>128	64	>128	>128
Ciprofloxacin	0.25	<0.125	<0.125	0.25	0.5	0.5	0.5	0.5	1	<0.125	<0.125	1

a MIC values were determined by broth microdilution in cation-adjusted Mueller-Hinton broth according to CLSI guidelines (*n* = 3). Antibiotic concentrations where GAS, MRSA, or VRE MIC changed from resistant to sensitive in the presence of PBT2-Zn are highlighted in bold.

b MIC for oxacillin against MRSA was determined in the presence of 2% NaCl per CLSI guidelines.

c Zinc concentration used for oxacillin was 25 μM and not 50 μM.

10.1128/mBio.02391-18.6 FIG S6 Reduced glutathione partially rescues GAS, MRSA, and VRE from PBT2-zinc-induced antibiotic resensitization. Time-kill curves of GAS, MRSA, and VRE in THY broth with or without PBT2 (2 μM for GAS or 6 μM for MRSA and VRE), ZnSO_4_ (400 μM for GAS and 600 μM for MRSA and VRE), antibiotic at 4 µg/ml (tetracycline for GAS, erythromycin for MRSA, and vancomycin for VRE), and reduced glutathione (1 mM, 5 mM, and 10 mM). Error bars indicate standard deviation from 2 biological replicates. Download FIG S6, PDF file, 0.2 MB. Copyright © 2019 Bohlmann et al. 2019 Bohlmann et al. This content is distributed under the terms of the Creative Commons Attribution 4.0 International license.

**FIG 3:**
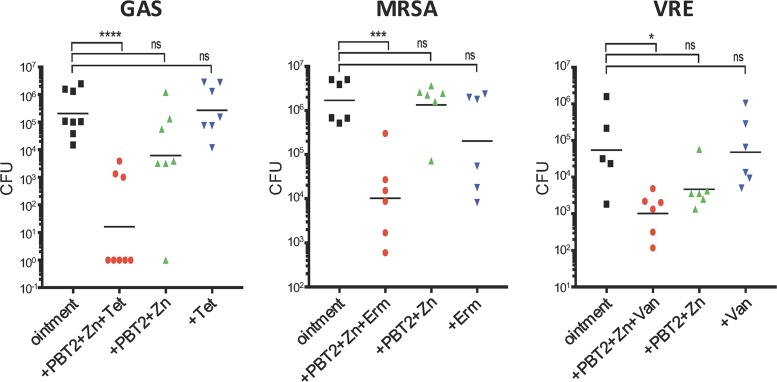
PBT2 and zinc reverse antibiotic resistance in a murine wound infection model. CFU recovered 4 days after wound infection with GAS (3.8 × 10^6^ CFU), MRSA (5.3 × 10^5^ CFU), or VRE (9.4 × 10^5^ CFU). Mice were treated twice daily with ointment only or ointment containing PBT2, Zn, and/or antibiotic (2 mM PBT2, 25 mM ZnSO_4_, and/or 15 μg/ml tetracycline for GAS; 3 mM PBT2, 30 mM ZnSO_4_, and/or 4 μg/ml erythromycin for MRSA; and 2 mM PBT2, 30 mM ZnSO_4_, and/or 20 μg/ml vancomycin for VRE). Values for individual mice are plotted, and black lines are representative of group geometric mean (*, *P* < 0.05; ***, *P* < 0.001; ****, *P* < 0.0001, one-way ANOVA). Abbreviations: Tet, tetracycline; Erm, erythromycin; Van, vancomycin.

To investigate the efficacy of PBT2-zinc in combination with antibiotics on infection, we again employed the murine wound infection model. Neither antibiotic nor subinhibitory concentrations of PBT2-zinc alone significantly reduced bacterial burden at the site of infection (Fig. 3). However, the combination of PBT2-zinc and tetracycline significantly reduced GAS infection; PBT2-zinc and erythromycin significantly reduced MRSA infection; and PBT2-zinc and vancomycin significantly reduced VRE infection (Fig. 3). Amounts of zinc and PBT2 utilized in each respective treatment were well below concentrations typically employed for the treatment of actinic keratosis (ZnSO_4_, 25% **[**wt/vol]) (22) and are significantly below the regimens tested in phase I and II clinical trials of PBT2 (8–10). Thus, concentrations of both PBT2 and zinc used in this study are likely to be well tolerated via topical delivery over a prolonged period of time. Overall, these data demonstrate the novel therapeutic synergy of PBT2-zinc in combination with antibiotics to topically treat otherwise resistant infections.

The steady and unrelenting rise in antibiotic resistance is one of the greatest global challenges confronting medicine and global heath. Strategies that are being explored to address the problem of bacterial resistance to existing antibiotics include the development of new antibiotics, blocking of resistance mechanisms against existing antibiotics, bacteriophage therapy, targeting of virulence mechanisms that weaken bacterial defense against host immunity, stimulating host immunity to improve bacterial clearance, destabilization of the Gram-negative cell envelope, vaccine development, and repurposing existing drugs used for noninfectious disease indications (6, 23–28). The Gram-positive bacterial pathogens GAS, MRSA, and VRE cause a wide range of hospital-acquired and community-acquired infections, place significant pressure and economic burden on health care systems, and are a major contributor to global human morbidity and mortality (29). Here we reveal that the safe-for-human-use ionophore PBT2, in combination with zinc, possesses antibacterial activity and at subinhibitory concentrations reverses Gram-positive bacterial resistance against a number of important antibiotics. The mechanism of action underlying this effect appears to be associated with dysregulation of transition metal ion homeostasis and profound disruption of essential virulence and metabolic systems, all of which may weaken the capacity of these bacterial pathogens to cause infection. Effects on antibiotic resistance were not universal. For instance, erythromycin resistance was reversed in MRSA but not in GAS. PBT2 is a safe-for-human-use ionophore that has progressed to phase 2 human clinical trials (9, 10). Zinc is used as a nutritional supplement and homeopathic remedy, and here we show that the delivery of zinc using the ionophore PBT2 can reduce the concentration of zinc required for efficacy to levels that are tolerated physiologically (30). Taken together, these data demonstrate a new paradigm whereby destabilizing bacterial physiology may circumvent the antibiotic resistance problem, by rescuing the function of antibiotics to which bacteria have become resistant.

## MATERIALS AND METHODS

### PBT2 synthesis.

PBT2 (compound 3) was synthesized following the reaction conditions shown below (13). Initially, oxidation of the methyl side chain of 5,7-dichloro-2-methylquinolin-8-ol (compound 1) was achieved by heating compound 1 with selenium dioxide in 1,4-dioxane to provide the required aldehyde (compound 2), in quantitative yield. The resultant crude product was then further reacted with dimethylamine-hydrochloride in 1,2-dichloroethane and triethylamine to yield a product that was reduced *in situ*, by treatment with sodium triacetoxyborohydride to provide the free amine of PBT2 as an oil. Upon acidification of the free amine with HCl, PBT2 hydrochloride-salt (compound 3) was obtained in 81% yield.


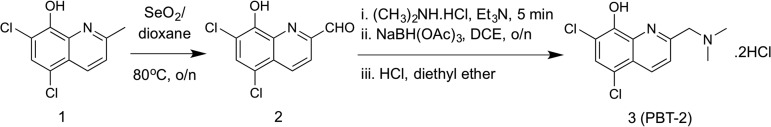


### (i) General synthetic methods.

Reagents and dry solvents purchased from commercial sources were used without further purification. Anhydrous reactions were carried out under an atmosphere of argon, using oven-dried glassware. Reactions were monitored using thin-layer chromatography (TLC) on aluminum plates precoated with Silica Gel 60 F254 (E. Merck). Developed plates were observed under UV light at 254 nm and then visualized after application of a solution of H_2_SO_4_ in EtOH (5% [vol/vol]) and heating. Flash chromatography was performed on Silica Gel 60 (0.040 to 0.063 mm) using distilled solvents. ^1^H and ^13^C NMR spectra were recorded at 400 and 100 MHz, respectively, on a Bruker Avance 400-MHz spectrometer. Chemical shifts (δ) are reported in parts per million, relative to the residual solvent peak as internal reference (CDCl_3_, 7.26 [s] for ^1^H, 77.0 [t] for ^13^C). Low-resolution mass spectra (LRMS) were recorded, in electrospray ionization mode, on a Bruker Daltonics Esquire 3000 ESI spectrometer, using positive ionization mode. The purity of the final product (compound 3) was determined to be >95% by ^1^H and ^13^C NMR. The synthetic work followed the procedures reported in the patent describing PBT2 preparation (13). The details of synthetic methods used and full characterization data of the final product are reported below.

### (ii) 5,7-Dichloro-8-hydroxy-2-quinolinecarboxaldehyde (compound 2).


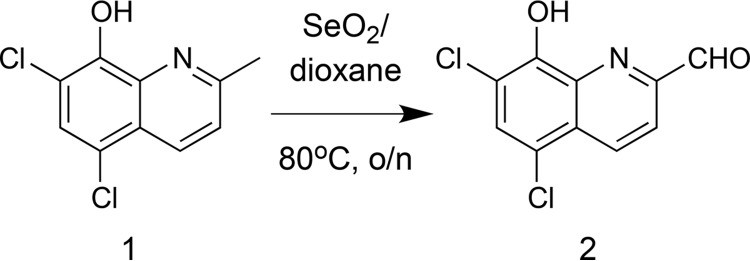


To a stirred suspension of selenium dioxide (1.75 g, 15.80 mmol) in 1,4-dioxane (80 ml) at 55°C was added a solution of 5,7-dichloro-2-methyl-quinolin-8-ol (2.0 g, 8.77 mmol) in 1,4-dioxane (20 ml) in a dropwise manner over a period of 3 h. After complete addition, the heating temperature was raised to 80°C, and heating was maintained overnight. The reaction mixture was then allowed to cool down to room temperature, and the insoluble solids were filtered off on a Celite bed. The filtrate was concentrated under vacuum, and the residue was washed with diethyl ether (10 ml × 3) to yield 2.10 g (quantitative yield) of the aldehyde 2 as a yellow powder, which was used in the following step without further purification.

### (iii) 5,7-Dichloro-2-((*N*,*N*-dimethylamino)methyl)quinolin-8-ol HCl salt (compound 3, PBT2 HCl).


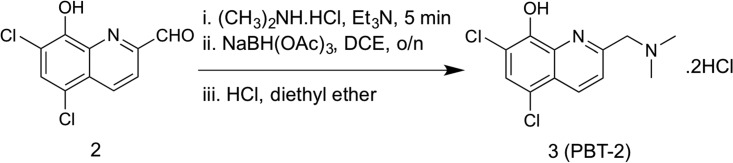


To a stirred solution of the crude aldehyde 2 (2.0 g, 8.26 mmol) and dimethylamine hydrochloride (730 mg, 8.96 mmol) in 1,2-dichloroethane (100 ml) was added triethylamine (1.25 ml, 8.96 mmol) in a dropwise manner. The mixture was stirred at room temperature (RT) for 5 min, and then sodium triacetoxyborohydride (2.4 g, 11.32 mmol) was added portionwise over a period of 5 min. The mixture was then stirred at RT overnight. Upon reaction completion, the reaction mixture was diluted with dichloromethane (200 ml) and washed with saturated sodium bicarbonate (100 ml × 3). The organic layer was then dried over anhydrous Na_2_SO_4_ and concentrated under vacuum to yield an oily product of the free amine base of PBT2. The oily product was triturated with water (100 ml) and extracted with diethyl ether (100 ml × 4). The ethereal extracts were combined, washed with brine, dried over Na_2_SO_4_, and concentrated under vacuum. To the obtained residue was added 10 ml of concentrated HCl, and the mixture was concentrated *in vacuo*. The resulting residue was washed with dichloromethane (50 ml × 3) to yield 2.30 g of PBT-HCl salt (compound 3) as pale yellow powder (81% yield). ^1^H NMR (400 MHz, CDCl_3_): δ 2.32 (s, 6H, 2NCH_3_), 3.78 (s, 2H, CH_2_), 7.51 (s, 1H, H-6), 7.61 (d, *J *=* *8.7 Hz, 1H, H-4), 8.39 (d, *J *=* *8.6 Hz, 1H, H-3); ^13^C NMR (101 MHz, CDCl_3_): δ 45.64 (NCH_3_ × 2), 65.46 (CH_2_), 115.51 (C-7), 120.35 (C-5), 122.26 (C-3), 124.07 (q carbon), 127.76 (C-6), 133.83 (C-4), 138.16 (q carbon), 147.98 (C-8), 158.93 (C-2); LRMS [C_12_H_12_Cl_2_N_2_O] (*m/z*): (positive ion mode) 272.0 [M+H]^+^.

### Bacterial strains, media, and growth conditions.

GAS HKU16, MRSA USA300, and VRE RBWH1 were grown in Todd-Hewitt broth (THB) or agar with 1% yeast extract (THY) (31) or in cation-adjusted Mueller-Hinton broth (CAMHB) (32) or agar (supplemented with 2.5% lysed horse blood [LHB] for GAS HKU16). Bacteria were routinely grown at 37°C under static conditions in ambient air.

### Sequencing of VRE.

To determine the complete genome sequence of RBWH1, long-read, single-molecule real-time (SMRT) sequencing was performed using the Pacific Biosciences RS II platform. Genomic DNA was manually sheared through a 26-gauge syringe, and a library was prepared using the SMRTbell DNA Template Prep kit 1.0 (Pacific Biosciences). Sequencing was performed on a single SMRT cell using polymerase P6 and V4 chemistry (Pacific Biosciences). Filtering of the long reads identified 147,593 reads with an average polymerase read length of 4.8 kb. To aid in genome assembly validation, RBWH1 was also sequenced on an Illumina Next-Seq 500 to produce paired-end reads with a read length of 150 bases. *De novo* genome assembly was performed using SMRT analysis v2.3.0 (Pacific Biosciences) and HGAP v3 and polished using Quiver. The chromosome and plasmids were circularized either manually or using Circlator (33), and the resulting genome assembly was validated using the Illumina short-read sequence data using Pilon v1.22 (34). Putative plasmid sequences were trimmed and orientated to published E. faecium plasmids. The final genome comprised a single circular chromosome of 2,876,110 bp and six plasmid sequences larger than 2,000 bp in length: pRBWH1.1, 167,502 bp; pRBWH1.2, 55,808 bp; pRBWH1.3, 39,621 bp; pRBWH1.4, 11,916 bp; pRBWH1.5, 4,453 bp; pRBWH1.6, 3,008 bp. Genome annotation was performed using Prokka (35).

### Drop test assay.

Bacteria were diluted from overnight cultures to obtain a starting OD_600_ of 0.01 in THY. Once the bacteria grew to an OD_600_ of 0.6, the cultures were serially diluted in PBS and 5 μl of each dilution (undiluted, 10^−1^, 10^−2^, 10^−3^, 10^−4^, 10^−5^) was plated on THY agar plates with or without zinc (400 μM) and/or PBT2 (1.5 μM) or vancomycin (20 μg/ml). Plates were incubated at 37°C overnight. Drop tests were undertaken in biological triplicates.

### Inductively coupled plasma mass spectrometry (ICP-MS).

Overnight cultures of bacteria were diluted to an OD_600_ of 0.05 in THY (GAS, 45 ml; MRSA and VRE, 20 ml) with or without PBT2 and zinc (GAS, 0.3 μM PBT2 + 50 μM ZnSO_4_; MRSA, 1 μM PBT2 + 200 μM ZnSO_4_; and VRE, 1 μM PBT2 + 150 μM ZnSO_4_) and grown to mid-log phase (GAS, OD_600_ = 0.5; MRSA and VRE, OD_600_ = 1.0). Cells were harvested at 7,000 × *g* for 7 min at 8°C. The pellet was resuspended in 20 ml PBS containing 5 mM EDTA and pelleted again at 7,000 × *g* for 7 min at 8°C. The wash was repeated two more times, and then the pellet was resuspended in 20 ml PBS without EDTA. The cells were harvested again at 7,000 × *g* for 7 min at 8°C, and then the pellet was washed with PBS. After centrifugation, the pellet was resuspended in 1 ml PBS and transferred into a microcentrifuge tube. The cells were pelleted at 18,000 × *g* for 7 min at 8°C, the supernatant was removed, and the pellet was dried at 96°C overnight. The dry pellet was weighed to determine the dry cell weight, resuspended in 1 ml of 35% HNO_3_, and carefully heated to 96°C for 60 min. After vortexing, the sample was centrifuged at 18,000 × *g* for 25 min to pellet cell debris. For ICP-MS analysis, 200 μl of the supernatant was diluted into 1.8 ml of double-distilled H_2_O. Samples were analyzed on an Agilent 8900 ICP-QQQ (Adelaide Microscopy, University of Adelaide). At least three biological replicates were analyzed.

### MIC determination.

MICs were determined by broth microdilution in accordance with CLSI guidelines (14). MIC assays were undertaken in 96-well plates in a total volume of 100 μl per well. For MRSA and VRE, the assays were performed in MHB, and for GAS, MHB plus 2.5% lysed horse blood (LHB) was used. The bacterial inoculum was prepared by direct colony suspension aiming for 2 × 10^5^ to 8 × 10^5^ CFU/ml bacteria per well. Antibiotics/compounds were serially diluted 2-fold across the 96-well plate; the last column contained no antibiotics/compounds. The inoculum was added to the plate containing antibiotic/compound and incubated for 16 to 24 h at 35 ±2°C. The MIC was determined as the lowest concentration of antibiotic/compound that showed no visible growth. MIC assays were carried out in biological triplicates.

### Resistance development studies.

The development of resistance to antibiotics/compounds was undertaken essentially as previously described (6). To investigate resistance development for GAS, MRSA, and VRE in the presence of subinhibitory concentrations of PBT2 and zinc, bacteria were sequentially passaged over 30 days in CAMHB. As a control, the antibiotic ciprofloxacin was used for GAS and MRSA, and chloramphenicol was used for VRE. Initially, the MIC for PBT2-zinc or antibiotic was determined by broth microdilution according to CLSI guidelines in a microtiter plate. The highest antibiotic or PBT2-zinc concentration that still showed growth after overnight incubation was diluted 1/250 into a new microtiter plate containing 2-fold dilutions of antibiotic or PBT2-zinc. This procedure was repeated for 30 days. The assays were undertaken in biological triplicates.

### Bacterial time-kill assays.

Bacteria were grown to mid-log phase in THY and then diluted to a starting OD_600_ of 0.05 in THY only, THY containing PBT2 (2 μM for GAS or 6 μM for MRSA and VRE) and/or ZnSO_4_ (400 μM for GAS and 600 μM for MRSA and VRE), or THY containing vancomycin (4 μg/ml for MRSA; 2× MIC) or tetracycline (1 μg/ml for MRSA; 2× MIC). To determine surviving numbers of bacteria, aliquots were removed at 0, 1, 2, 4, 6, and 24 h; serially diluted in PBS; and plated onto THY agar plates. Viable bacteria were counted after overnight incubation at 37°C. Time-kill assays were performed in biological duplicates.

### Cytotoxicity assay.

TEpi cells were incubated with increasing concentrations of PBT2-zinc (3 μM PBT2 + 300 μM ZnSO_4_ or 6 μM PBT2 + 600 μM ZnSO_4_) at 37°C, 5% CO_2_, for 24 h. Cell death was quantified at 4 h and 24 h posttreatment by measuring lactate dehydrogenase (LDH) release from cell supernatants as previously described (36). A two-way ANOVA with Sidak’s posttest was performed to detect statistical significance.

### Ethics.

Animal experiments were performed according to the Australian code of practice for the care and use of animals for scientific purposes. Permission was obtained from the University of Queensland ethics committee (SCMB/140/16/NHMRC).

### Murine wound infection model.

For wound infection (17), 4- to 7-week-old female BALB/c mice were used and housed in individual cages. The neck area of the mice was shaved, and residual hair was removed using Nair (Church & Dwight) prior to the experiment. On the day of infection, mice were anesthetized by inhalation of methoxyflurane, and a small superﬁcial scarification was made on the shaved skin using a metal ﬁle. For infection, bacteria were cultured to mid-log phase in THY medium and 5 × 10^6^ to 2 × 10^7^ CFU GAS or 5 × 10^5^ to 2 × 10^6^ CFU MRSA/VRE was applied onto the scarified skin (exact inoculum is specified in figure legends). After the inoculum had been absorbed by the skin (approximately 10 min), the mice were treated with carrier ointment only (Pharmacy Choice aqueous cream) or ointment containing PBT2 and/or zinc and/or antibiotic (tetracycline for GAS, erythromycin for MRSA, vancomycin for VRE). Mice were treated with ointment twice daily, and a total of 9 treatments were applied. Each treatment consisted of around 25 to 30 mg ointment and contained 5 mM PBT2 and/or 50 mM Zn (as ZnSO_4_ for MRSA and ZnCl_2_ for GAS) and 2.75 mM PBT2 and/or 75 mM ZnSO_4_ for VRE. For experiments including antibiotics, the ointment contained 2 mM PBT2 and/or 25 mM ZnSO_4_ and/or 1.5% tetracycline for GAS, 3 mM PBT2 and/or 30 mM ZnSO_4_ and/or 0.4% erythromycin for MRSA, and 2 mM PBT2 and/or 30 mM ZnSO_4_ and/or 2% vancomycin for VRE. After 4 days of treatment, the mice were euthanized, and the scarified skin was excised and washed twice in PBS by vortexing for 30 s. The skin was then homogenized in lysing matrix F tubes using a FastPrep instrument (MP Biomedicals) and plated out on antibiotic selective THY plates to determine viable bacteria (for GAS, homogenates were plated out on THY with 10 μg/ml neomycin; for MRSA and VRE, on THY with 10 μg/ml ampicillin). Each treatment group contained five to eight mice, and statistical significance was calculated with one-way ANOVA using log-transformed values.

### RNA isolation.

RNA was isolated using the FastRNA Pro Blue kit (MP Biomedicals) and the SV total RNA isolation system (Promega). Briefly, bacteria were grown to mid-log phase (OD_600_ of 0.4 to 0.5) in MHB (+2.5% LHB for GAS) in the presence or absence of PBT2 and/or ZnSO_4_. Two volumes of RNAprotect (Qiagen) was added to the cultures, and the samples were then centrifuged at 5,000 × *g* for 25 min at 4°C to pellet cells. The dry pellet was stored at −80°C overnight and then resuspended in 1 ml RNA Pro solution (FastRNA Pro Blue kit). The sample was transferred to lysing matrix B and processed in a FastPrep instrument (MP Biomedicals). After centrifugation at 13,000 × *g* for 15 min at 4°C, the supernatant was transferred into a fresh tube and incubated at room temperature for 5 min. Three hundred microliters of chloroform was added, and the mixture was vortexed for 10 s. After 5 min incubation at room temperature, the upper phase was moved to a fresh tube containing 200 μl cold 95% EtOH. The sample was placed on ice for at least 5 min and then transferred into an SV total RNA isolation system spin column. The sample was processed according to the manufacturer’s instructions and eluted in 110 μl of nuclease-free water. To ensure complete removal of DNA, the RNA was then further purified using the TURBO DNA-free kit (Thermo Fisher Scientific) according to the manufacturer’s instructions.

### RNASeq analysis.

RNASeq analysis was performed at the Australian Genome Research Facility. The library was prepared using a Ribo Zero stranded protocol. In brief, rRNA was depleted with Ribo Zero, RNA was fragmented (heat and divalent cations), and first-strand cDNA synthesis was done with SuperScript II reverse transcriptase (Invitrogen). For the second-strand cDNA synthesis, the strand was “marked” with dUTP. A 3′ adenylation of DNA fragments was performed followed by sequencing adapter ligation (utilizing T-A pairing of adapter and DNA fragments). The library was amplified by PCR (amplification of “unmarked” first strand only). Libraries were assessed using either Agilent’s Bioanalyzer DNA 1000 chip or the TapeStation D1K TapeScreen system. qPCR was used to quantify individual libraries before normalizing (2 nM) and pooling. Libraries were pooled and clustered through the Illumina cBot system using TruSeq PE Cluster kit v3 reagents followed by sequencing on the Illumina HiSeq 2500 system with TruSeq SBS kit v3 reagents with 110 (101 read 1, 9 cycles index read). Libraries were sequenced with a HiSeq 2500 ultrahigh-throughput sequencing system (Illumina) to produce 100-base-paired-end reads. An average of 45 million reads per sample were generated and mapped to the reference genome (Sscrofa10.2) using the 2-pass method of the STAR aligner with default parameters. Eighty percent of these reads uniquely hit to the reference genome. Duplicate reads were denoted with the MarkDuplicates tool of Picard (http://broadinstitute.github.io/picard). Differential gene expression was analyzed using Degust (http://victorian-bioinformatics-consortium.github.io/degust/), and figures were generated using RStudio (37).

### Real-time PCR.

Only selected genes associated with heavy metal homeostasis, virulence systems, and metabolic processes were selected for real-time PCR analysis. Briefly, 1 μg of isolated RNA was transformed into cDNA using the SuperScript III first-strand synthesis kit (Invitrogen). Real-time PCR was undertaken with the SYBR Green master mix (Applied Biosystems) following the manufacturer’s instructions. Measurements were performed using the ViiA7 real-time PCR system (Life Technologies), using the following conditions: 95°C for 10 min, 40 cycles of 95°C for 15 s, and 60°C for 1 min, and a final dissociation cycle of 95°C for 2 min, 60°C for 15 s, and 95°C for 15 s. Relative gene expression was calculated by the ΔΔ*C_T_* method using *proS* (GAS), *rrsA* (MRSA), and 23S (VRE) as the reference genes. All experiments were done in biological triplicates and measured in technical triplicates. Primers used for real-time PCR are given in Table S3.

10.1128/mBio.02391-18.9 TABLE S3 Real-time PCR primers used in this study. Download Table S3, PDF file, 0.1 MB. Copyright © 2019 Bohlmann et al. 2019 Bohlmann et al. This content is distributed under the terms of the Creative Commons Attribution 4.0 International license.

### Glutathione supplementation assay.

Bacteria were grown to mid-log phase in THY and then diluted to a starting OD_600_ of 0.05 in THY only or THY containing PBT2 (2 μM for GAS or 6 μM for MRSA and VRE), ZnSO_4_ (400 μM for GAS and 600 μM for MRSA and VRE), antibiotic at 4 µg/ml (tetracycline for GAS, erythromycin for MRSA, and vancomycin for VRE), and reduced glutathione (1 mM, 5 mM, and 10 mM). To determine surviving numbers of bacteria, aliquots were removed at 0, 1, 2, 4, 6, and 24 h; serially diluted in PBS; and plated on THY agar plates. Viable bacteria were counted after overnight incubation at 37°C. Glutathione supplementation assays were performed in biological duplicates.

### Cell membrane permeability assays.

Membrane permeability of bacterial strains was measured using the Live/Dead BacLight bacterial viability kit (L7007) (ThermoFisher Scientific) according to the manufacturer’s instructions. Strains were grown as previously described (in CAMHB [+2.5% LHB for GAS]) with the exception of MRSA USA300, which was grown in tryptic soy broth (TSB). Mid-exponential-phase cells were harvested (4,000 × *g*, 10 min, 4°C), washed, and resuspended to an OD_600_ of 0.6 in potassium phosphate buffer (100 mM, pH 7.5), containing MgCl_2_ (2 mM) with the addition of 10 mM NaCl for MRSA USA300. A 0.4 ml volume of the cell suspension was removed and maintained on ice as the *t* = 0 sample. The cell suspension was energized by a 10-min incubation with glucose (20 mM). Aliquots (5 ml) were either untreated or treated with relevant concentrations of PBT2 and zinc (alone or in combination), nigericin (10 μM), or nisin (25 μg/ml) for 1 h under the same conditions in which they were grown. Aliquots (100 μl) of the *t* = 0 and 60-min samples were resuspended with 2× Live/Dead BacLight staining reagent (100 μl) in a 96-well, flat-bottom microtiter plate. Following incubation in the dark (15 min, RT), the fluorescence emission was measured using a Varioskan Flash plate reader (ThermoFisher Scientific). Membrane permeability was measured as a relative fluorescence emission ratio (530/630 nm) of the fluorescent dyes SYTO9 (which stains cells with intact or damaged membranes) and propidium iodide (which stains only cells with damaged membranes) and expressed as the relative change in membrane integrity after 1-h treatment with compounds. All experiments were done in biological triplicates and measured in technical triplicates.

### Scanning electron microscopy (SEM).

SEM studies were undertaken at the Centre for Microscopy and Microanalysis at the University of Queensland. Bacterial strains were cultured in THY medium to mid-exponential phase and treated with PBT2 (6 µM) and ZnSO_4_ (500 µM) for up to 24 h at 37°C. Bacteria were washed twice preceding glutaraldehyde fixation followed by secondary fixation in 1% osmium tetroxide. Samples were then dehydrated via a series of ethanol treatments and were applied to coverslips coated with poly-l-lysine (1 mg/ml) before being dried in a critical point dryer (Tousimis) according to the manufacturer’s instructions. Coverslips were attached to stubs with double-sided carbon tabs and coated with gold using a sputter coater (SPI) following manufacturer’s instructions. Samples were imaged in a JEOl JSM 7001F or JEOl JSM 7100F field emission SEM at an accelerating voltage of 1 kV.

### Accession number(s).

The genome of RBWH1 is publicly available under the BioProject identifier PRJNA497960 (accession numbers CP033206 to CP033212). RNASeq data are available at the GEO repository (GSE121555).
