# Supplementary figures and images for: Chemical Synergy between Ionophore PBT2 and Zinc Reverses Antibiotic Resistance
Source: mBio. 2018 Dec 11;9(6):e02391-18. doi: 10.1128/mBio.02391-18 (PMC6299484; doi:10.1128/mBio.02391-18)

# Supplementary Figure 1

**a**

IE1294.257.fid  
IE1294-100  
CDCl<sub>3</sub>  
IE1294 #257-60

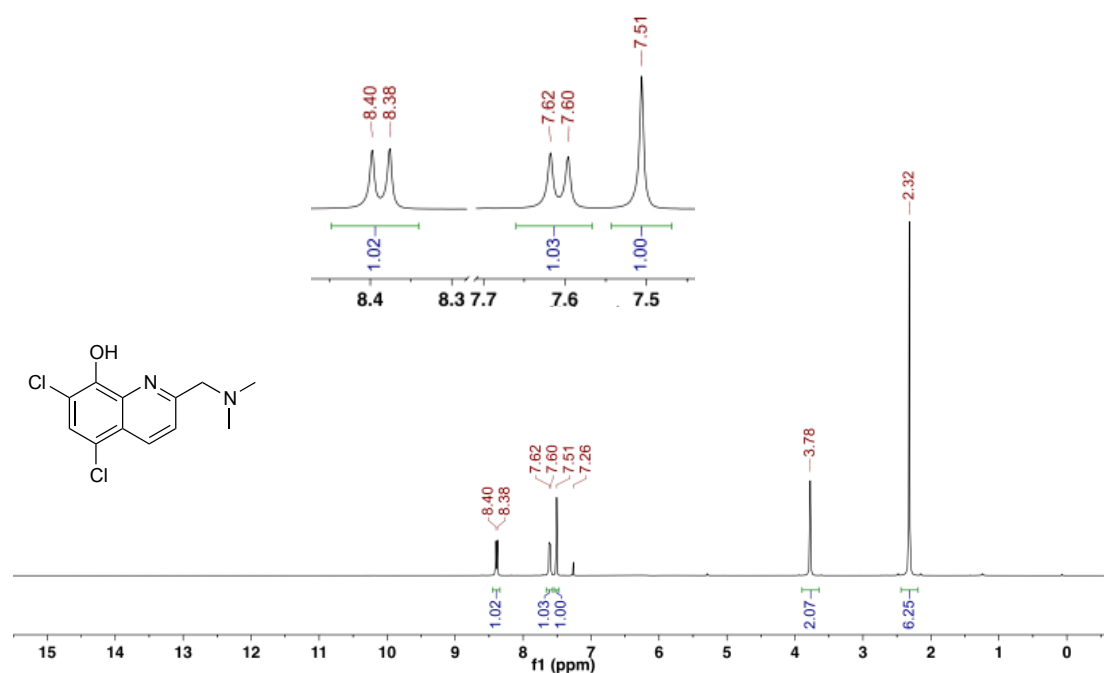

**b**

IE1294.259.fid  
IE1294-100  
CDCl<sub>3</sub>  
IE1294 #257-60

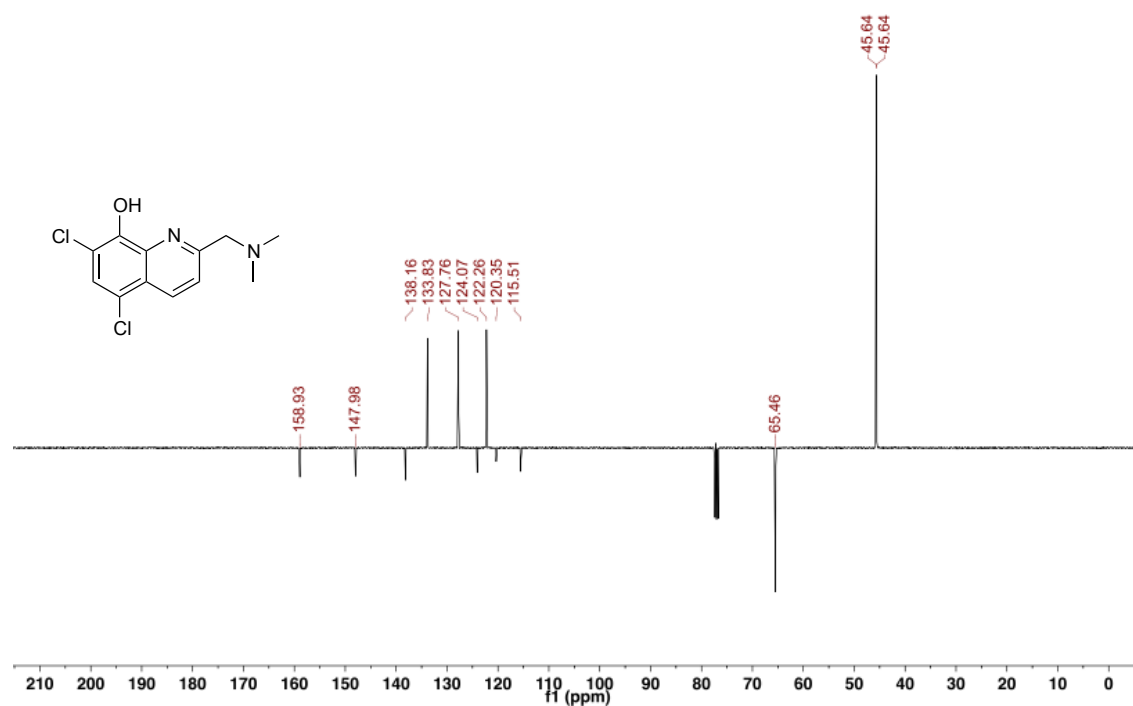

Supplement: FIG S1 [file mbo006184211sf1.pdf]

*Supplementary Figure 2*

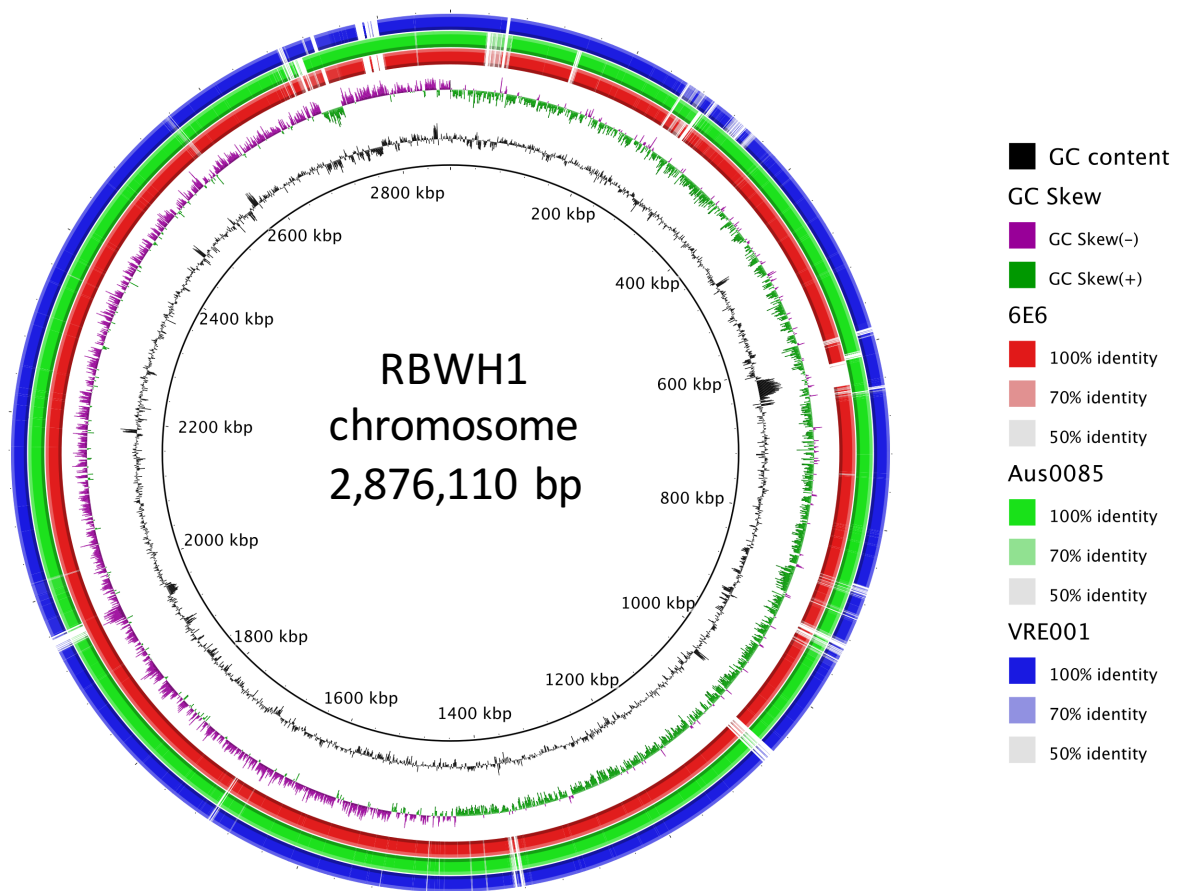

Supplement: FIG S2 [file mbo006184211sf2.pdf]

*Supplementary Figure 3*

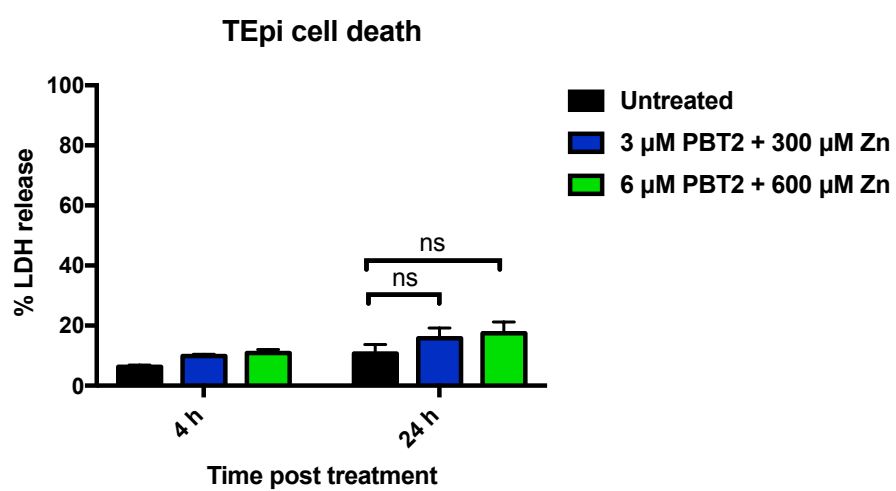

Supplement: FIG S3 [file mbo006184211sf3.pdf]

Supplementary Figure 4

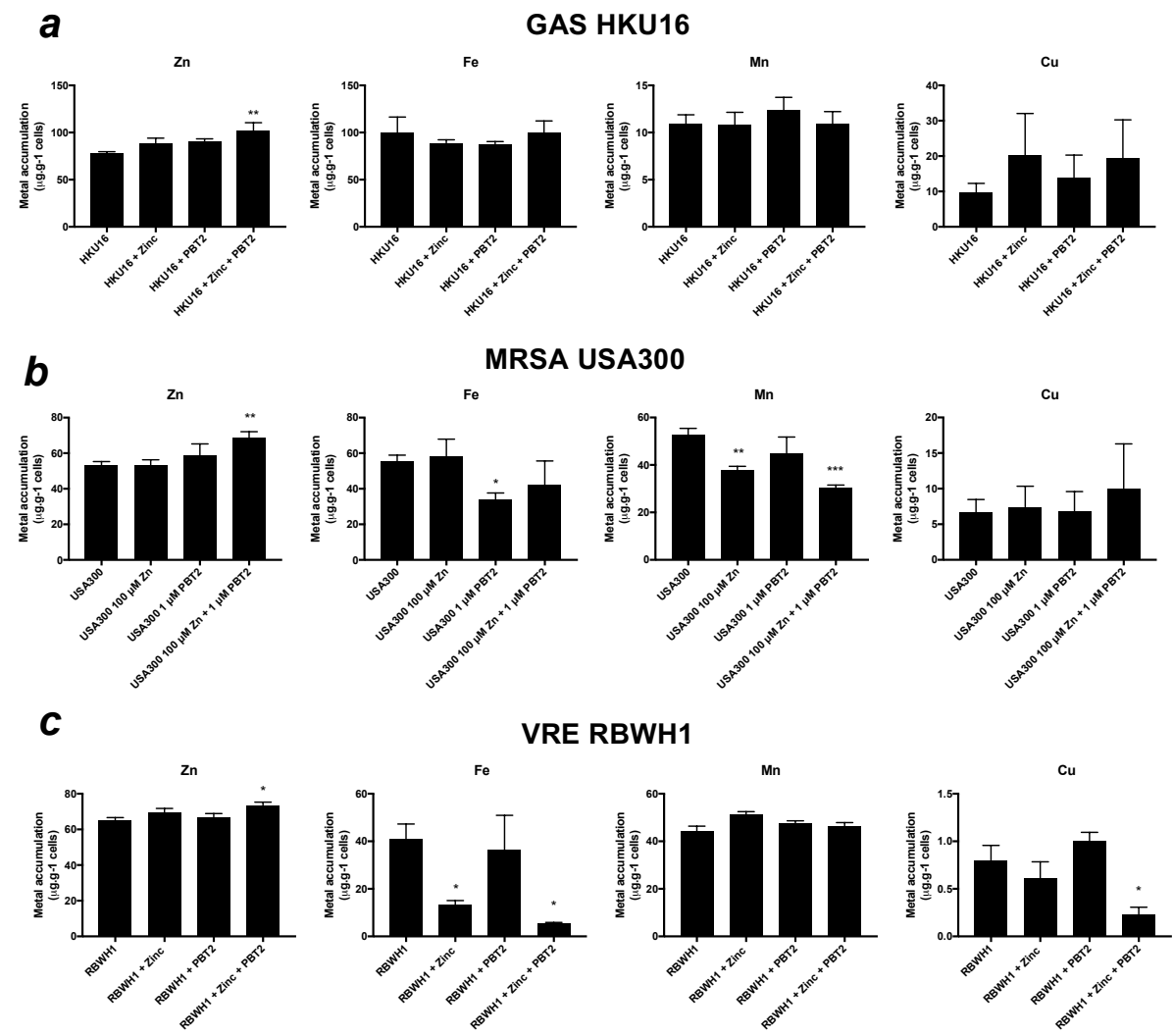

Supplement: FIG S4 [file mbo006184211sf4.pdf]

**Supplementary Figure 5**

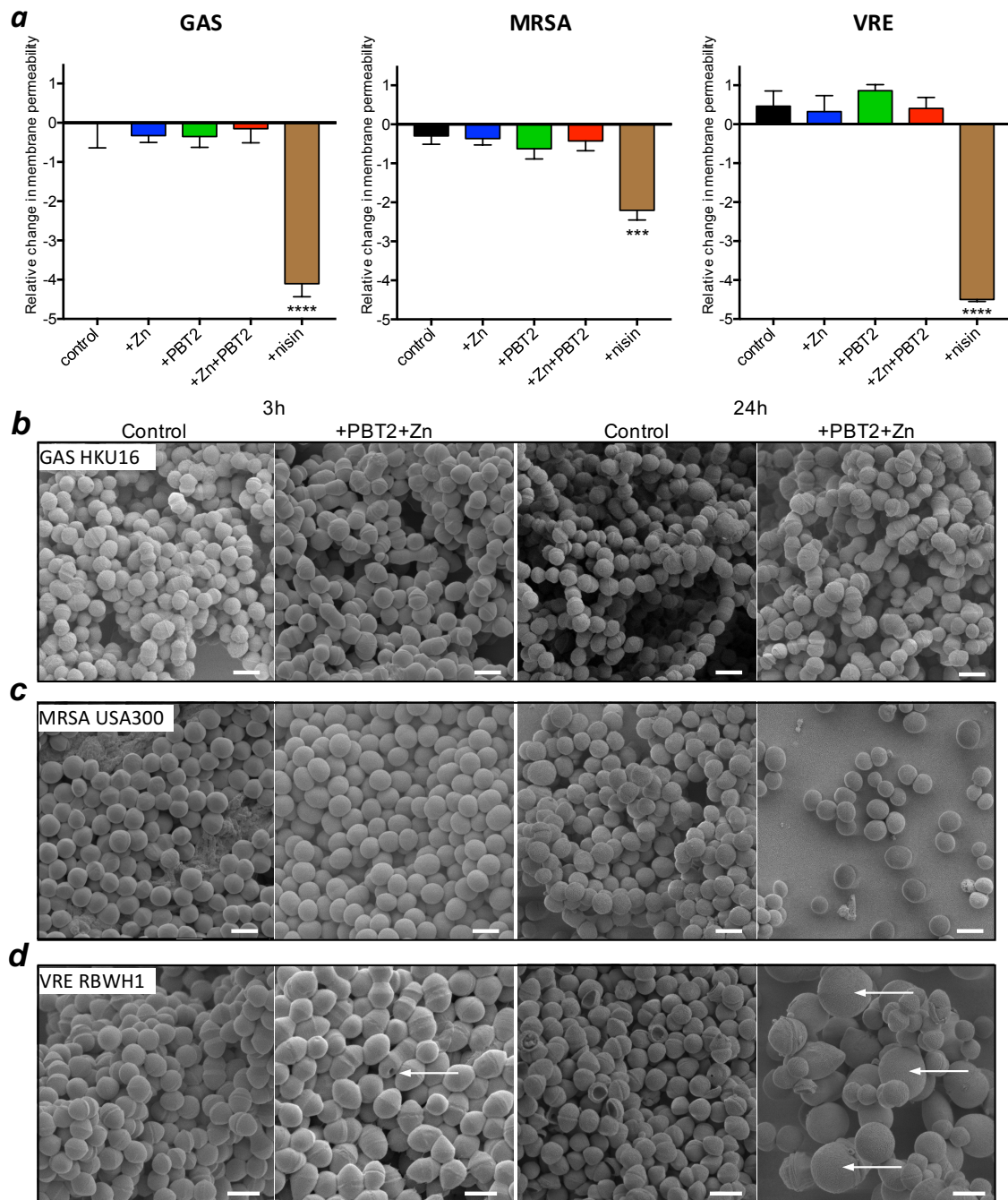

Supplement: FIG S5 [file mbo006184211sf5.pdf]

**Supplementary Figure 6**

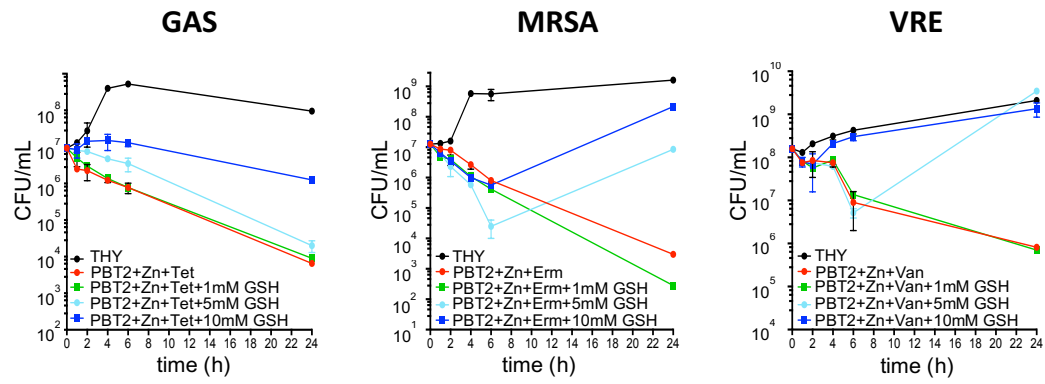

Supplement: FIG S6 [file mbo006184211sf6.pdf]
