# Supplementary material for: Chemical Synergy between Ionophore PBT2 and Zinc Reverses Antibiotic Resistance
Source: mBio. 2018 Dec 11;9(6):e02391-18. doi: 10.1128/mBio.02391-18 (PMC6299484; doi:10.1128/mBio.02391-18)
Supplement: TABLE S1 [file mbo006184211st1.pdf]

Supplementary Tables

Supplementary Table 1. PBT2 and zinc are active against Gram-positive bacterial pathogens.

	**GAS**	**MRSA**	**VRE**
**PBT2**	>30 µM	3.75 µM	3.75 µM
**Zinc**	2 mM	3 mM	3 mM
**PBT2+Zn**	4 µM PBT2+800 µM Zn	1 µM PBT2+800 µM Zn	1 µM PBT2+800 µM Zn
**PBT2+Zn**	8 µM PBT2+25 µM Zn	2 µM PBT2+200 µM Zn	2 µM PBT2+200 µM Zn

MIC values were determined by broth micro dilution in cation-adjusted Mueller Hinton broth according to CLSI guidelines, n=3.
