# Supplementary material for: Chemical Synergy between Ionophore PBT2 and Zinc Reverses Antibiotic Resistance
Source: mBio. 2018 Dec 11;9(6):e02391-18. doi: 10.1128/mBio.02391-18 (PMC6299484; doi:10.1128/mBio.02391-18)
Supplement: TABLE S2 [file mbo006184211st2.pdf]

Supplementary Table 2. Differentially expressed genes after treatment with PBT2 and zinc.

Supplementary Table 2a. GAS HKU16 up-regulated genes following 1 h treatment with 4.75 μM PBT2 and 128 μM zinc in CAMHB+LHB grown at 37°C.

Locus_tag*	LogFC**	FDR***	Gene	Putative gene product function
5350	3.84	1.67E-06	*dacC*	Penicillin-binding protein 5, C-terminal domain
5355	3.70	1.67E-06	-	hypothetical protein
2880	3.21	3.36E-06	*czcD*	zinc exporter
3200	2.53	1.67E-06	-	hypothetical protein
4080	2.42	4.09E-06	-	hypothetical protein
6390	2.41	4.44E-06	*copY*	transcriptional regulator (copper export)
109275	2.40	4.82E-05	*Yko1*	Uncharacterized membrane protein YkoI
8610	2.33	3.36E-06	-	dipicolinate synthase
4750	2.19	3.36E-06	*glnH*	amino acid ABC transporter permease
7620	2.17	1.67E-06	-	MerR family transcriptional regulator
7615	2.17	3.36E-06	*glnA*	type I glutamate--ammonia ligase
8475	2.16	4.84E-05	-	YSIRK-targeted surface antigen transcriptional regulator
6380	2.09	1.18E-05	*copZ*	Copper chaperone CopZ
4755	2.06	5.46E-06	-	amino acid ABC transporter ATP-binding protein

3080	1.98	1.22E-05	*rpiA*	ribose-5-phosphate isomerase
2055	1.97	4.09E-06	-	hypothetical protein
2155	1.97	4.09E-06	-	hypothetical protein
6385	1.97	1.69E-05	*copA*	copper-translocating P-type ATPase
5640	1.94	8.75E-05	*dpr*	DNA starvation/stationary phase protection protein
3085	1.84	1.43E-05	*deoB*	phosphopentomutase
4010	1.82	2.53E-05	-	lipase/acylhydrolase
6915	1.70	7.77E-05	-	universal stress protein
500	1.69	1.85E-04	*groEL*	molecular chaperone GroEL
3900	1.67	3.64E-05	-	hypothetical protein
3090	1.66	1.08E-05	*arsC*	arsenate reductase (glutaredoxin)
970	1.66	1.13E-04	-	aldehyde dehydrogenase
7890	1.66	7.15E-06	-	23S rRNA (guanosine(2251)-2'-O)-methyltransferase RlmB
5550	1.64	4.09E-06	*mutT*	NUDIX hydrolase
3095	1.63	1.22E-05	*punA*	purine-nucleoside phosphorylase
495	1.62	9.52E-05	*groES*	co-chaperone GroES
2110	1.61	1.90E-05	-	hypothetical protein
6015	1.60	1.38E-05	-	YigZ family protein
1675	1.55	8.91E-05	-	oleate hydratase
5555	1.55	2.53E-05	-	hypothetical protein
1290	1.51	3.38E-05	-	membrane protein
2150	1.50	2.18E-05	-	membrane protein
1155	1.50	2.56E-04	-	O-sialoglycoprotein endopeptidase
6065	1.49	2.18E-05	*fmt*	methionyl-tRNA formyltransferase
395	1.48	1.97E-05	*dexS*	glucohydrolase
3740	1.46	7.38E-05	*csrA*	peptide-methionine (R)-S-oxide reductase
1160	1.45	3.18E-04	-	methionine import ATP-binding protein MetN
1285	1.42	1.18E-05	-	hypothetical protein
7885	1.41	1.97E-05	-	hypothetical protein
2090	1.40	1.67E-05	-	YtxH domain-containing protein
465	1.38	1.90E-03	*nox1*	alkyl hydroperoxide reductase subunit F
2305	1.38	4.82E-05	-	MBL fold metallo-hydrolase
8655	1.37	7.05E-05	-	4-diphosphocytidyl-2C-methyl-D-erythritol kinase
470	1.35	1.43E-03	*ahpC*	peroxiredoxin
7230	1.34	9.81E-04	-	hypothetical protein
8660	1.31	5.79E-05	-	4-diphosphocytidyl-2C-methyl-D-erythritol kinase
1165	1.31	5.53E-04	-	methionine ABC transporter permease
5245	1.29	5.36E-04	-	phosphocarrier protein HPr
1475	1.29	8.75E-05	-	copper homeostasis protein CutC
8640	1.29	3.06E-05	*adcB*	zinc ABC transporter permease
745	1.25	8.91E-05	*speG*	exotoxin
5545	1.25	1.35E-04	*clpE*	ATP-dependent Clp protease ATP-binding subunit
2180	1.24	4.51E-04	-	elongation factor Tu
3050	1.22	1.97E-04	*dyr*	dihydrofolate reductase

4600	1.22	5.21E-05	-	cation transporter
8645	1.20	1.97E-05	*adcC*	zinc ABC transporter ATP-binding protein
1630	1.20	1.70E-04	-	membrane protein
8915	1.18	4.84E-05	-	low molecular weight phosphotyrosine protein phosphatase
7550	1.18	9.34E-05	*dnaQ*	3'-5' exonuclease
3955	1.17	1.59E-04	*murB*	UDP-N-acetylenolpyruvoylglucosamine reductase
8230	1.16	1.57E-04	-	integrase
7860	1.16	4.23E-05	-	30S ribosomal protein S9
9675	1.16	1.41E-04	-	hypothetical protein
4070	1.15	6.75E-04	*pta*	phosphate acetyltransferase
7865	1.15	2.53E-05	-	50S ribosomal protein L13
205	1.13	2.29E-04	-	coenzyme A pyrophosphatase
7485	1.12	6.27E-05	*dinP*	DNA polymerase IV
3610	1.12	8.53E-05	-	pyruvate dehydrogenase E1 subunit alpha
1005	1.12	3.64E-05	-	ABC transporter ATP-binding protein
8010	1.11	5.79E-05	-	thiol reductase thioredoxin
4655	1.11	4.14E-04	-	GntR family transcriptional regulator
2085	1.10	2.50E-05	-	DUF948 domain containing protein
2075	1.10	4.77E-05	*ptsK*	HPr kinase/phosphorylase
1175	1.10	8.43E-05	-	serine/threonine transporter SstT
8650	1.09	5.21E-05	*adcR*	transcriptional regulator
9375	1.09	1.19E-04	-	hypothetical protein
2070	1.09	2.55E-05	-	membrane protein
5455	1.08	4.54E-04	-	heavy metal translocating P-type ATPase
3745	1.08	5.61E-05	-	membrane protein
4660	1.07	5.71E-04	-	ABC transporter ATP-binding protein
3250	1.07	2.57E-05	*apt*	adenine phosphoribosyltransferase
3135	1.07	6.16E-05	-	ABC transporter substrate-binding protein
7545	1.06	7.38E-05	-	MerR family transcriptional regulator
2615	1.06	7.39E-04	*mscL*	large-conductance mechanosensitive channel
5440	1.05	3.11E-03	-	transcriptional regulator
5170	1.04	3.30E-04	*grab*	protein G alpha 2M-binding protein
8910	1.04	4.82E-05	-	membrane protein
9665	1.04	1.10E-04	-	DUF3042 domain-containing protein
2145	1.03	9.15E-05	-	N-acetylmuramoyl-L-alanine amidase
5910	1.02	7.00E-03	-	glycosyl hydrolase
1425	1.01	8.44E-05	*clpP*	ATP-dependent Clp protease proteolytic subunit
5300	1.01	2.55E-05	*alaS*	alanine-tRNA ligase
825	1.01	1.04E-04	*gltX*	glutamate-tRNA ligase
1375	1.00	8.75E-05	*pflC*	pyruvate formate lyase-activating protein

*Locus_tag: identifier corresponding to reference genome sequence, prefix to all numbers listed is “SPYOHK_RS”, **logFC: log_2 fold change, ***FDR: false discovery rate.

Supplementary Table 2b. GAS HKU16 down-regulated genes following 1 h treatment with 4.75 µM PBT2 and 128 µM zinc in CAMHB+LHB grown at 37°C.

Locus_tag*	LogFC**	FDR***	Gene	Putative gene product function
7930	-3.77	9.05E-05	-	transaldolase
3990	-3.73	4.09E-06	*sfcA*	malate dehydrogenase
7935	-3.69	8.15E-05	*ulaA*	PTS ascorbate transporter subunit IIC
7940	-3.54	1.07E-04	-	PTS maltose transporter subunit IIBC
7945	-3.44	8.91E-05	*bglG*	PTS fructose transporter subunit IIA
8310	-2.63	1.64E-05	*sgaB*	PTS ascorbate transporter subunit IIB
510	-2.57	7.15E-06	*pepD*	dipeptidase
2470	-2.42	4.64E-05	*sagH*	ABC transporter permease
8290	-2.41	5.16E-04	*araD*	L-ribulose-5-phosphate 4-epimerase
2465	-2.36	8.07E-05	*ccmA*	ABC transporter ATP-binding protein
2460	-2.32	6.55E-05	-	hypothetical protein
1605	-2.31	3.36E-06	*mtsA*	metal ABC transporter substrate-binding protein
9380	-2.29	1.18E-05	-	hypothetical protein
8630	-2.21	8.91E-05	-	tyrosine--tRNA ligase
2475	-2.19	8.18E-05	-	ABC transporter permease
8295	-2.16	2.14E-04	-	L-xylulose 5-phosphate 3-epimerase
2680	-2.12	1.14E-04	-	membrane protein
8300	-2.11	4.24E-04	*ulaD*	3-keto-L-gulonate-6-phosphate decarboxylase
9010	-2.10	1.07E-04	-	amidase
6340	-2.10	1.67E-04	*lacD.1*	tagatose-bisphosphate aldolase
3865	-2.08	3.63E-05	-	serine/threonine dehydratase
2675	-1.99	1.08E-05	-	glycosyl transferase family 2
8025	-1.98	1.79E-04	-	ABC transporter ATP-binding protein
2450	-1.98	7.32E-05	-	streptolysin associated protein SagD
2455	-1.97	5.79E-05	-	CPBP family intramembrane metalloprotease
3860	-1.96	9.33E-06	-	lanthionine synthetase
8895	-1.96	4.84E-05	*adhA*	zinc-dependent alcohol dehydrogenase
2670	-1.95	1.64E-05	-	phosphoglycerol transferase
695	-1.90	4.12E-05	*emm12*	YSIRK signal domain/LPXTG anchor domain surface protein
6350	-1.90	1.80E-04	-	galactose-6-phosphate isomerase
8315	-1.88	2.44E-04	-	PTS ascorbate transporter subunit IIC
460	-1.87	5.62E-04	*hutI*	imidazolonepropionase
8280	-1.87	3.73E-04	-	L-ascorbate-6-phosphate lactonase
2445	-1.83	9.42E-05	*sagC*	streptolysin associated protein SagC
3855	-1.81	4.06E-05	-	ABC transporter ATP-binding protein
5520	-1.79	9.33E-06	-	phosphomannomutase
2440	-1.72	1.10E-04	*sagB*	streptolysin associated protein SagB
5680	-1.72	1.05E-03	*asnA*	asparagine synthetase A
8305	-1.70	2.47E-04	-	PTS ascorbate transporter subunit IIA

6355	-1.70	4.96E-04	*lacA.1*	galactose-6-phosphate isomerase subunit LacA
2685	-1.69	1.08E-05	-	transcriptional activator amrA
6150	-1.68	1.61E-05	-	aminopeptidase C
8480	-1.67	4.24E-06	-	hypothetical protein
8485	-1.64	3.84E-06	-	SrtB family sortase
2665	-1.60	4.37E-05	*rgpFc*	alpha-L-Rha alpha-1,3-L-rhamnosyltransferase
4355	-1.53	4.82E-05	*lplA*	lipoate--protein ligase A
6250	-1.53	5.36E-04	-	transaldolase
2690	-1.52	4.12E-05	-	hypothetical protein
4865	-1.48	2.31E-04	-	multidrug transporter MatE
45	-1.47	2.02E-03	-	YitT family protein
3885	-1.47	1.84E-04	*srtE*	lantibiotic ABC transporter permease
5450	-1.46	2.82E-03	*pyrD*	dihydroorotate oxidase
6255	-1.45	7.08E-04	-	DNA-binding protein
2415	-1.45	1.61E-03	-	hypothetical protein
35	-1.42	4.84E-04	-	copper ABC transporter permease
5875	-1.40	3.74E-03	-	ABC transporter substrate-binding protein
3850	-1.39	3.18E-04	*srtA*	lantibiotic streptin
4700	-1.39	3.93E-03	*malF*	sugar ABC transporter permease
5220	-1.38	6.65E-04	*udk*	uridine kinase
4360	-1.38	5.21E-05	-	deacetylase SIR2
2515	-1.38	3.92E-03	*atpH*	ATP synthase subunit delta
3880	-1.37	2.27E-04	*srtF*	lantibiotic transport ATP-binding protein SrtF
4350	-1.36	1.54E-05	*fhs.1*	formate-tetrahydrofolate ligase
3890	-1.35	8.53E-05	*srtG*	lantibiotic ABC transporter permease
4705	-1.35	2.59E-03	-	maltose ABC transporter permease
4870	-1.35	1.40E-04	-	ABC transporter ATP-binding protein
8490	-1.34	2.37E-05	-	QVPTGV class sortase B protein-sorting domain-containing protein
2535	-1.33	1.17E-03	*atpC*	ATP synthase epsilon chain
4695	-1.32	5.63E-03	-	maltose/maltodextrin-binding protein
2660	-1.32	5.36E-05	*rgpEc*	glycosyl transferase
6260	-1.30	1.69E-03	-	NAD(P)/FAD-dependent oxidoreductase
2345	-1.29	1.10E-04	-	dipeptidase
40	-1.28	1.81E-04	-	ABC-F family ATPase
2655	-1.27	5.21E-05	*rgpDc*	ABC transporter ATP-binding protein
5210	-1.27	4.56E-04	-	PrsW family intramembrane metalloprotease
8495	-1.27	1.11E-04	-	signal peptidase I
3650	-1.26	7.52E-05	-	membrane protein
2530	-1.26	1.24E-03	*atpD*	ATP synthase subunit beta
2510	-1.26	3.63E-03	*atpF*	ATP synthase subunit B
6820	-1.25	2.00E-02	-	acyl carrier protein
5500	-1.25	5.79E-05	*fps*	geranyltranstransferase
4365	-1.25	8.91E-05	-	hypothetical protein
3655	-1.23	6.55E-05	-	hypothetical protein

2500	-1.21	6.40E-03	*atpE*	F0F1 ATP synthase subunit C
2505	-1.20	5.78E-03	*atpB*	ATP synthase subunit A
6920	-1.19	4.88E-04	-	haloacid dehalogenase
2650	-1.19	1.42E-04	*rgpCc*	LPS ABC transporter
3895	-1.18	7.99E-04	-	transcriptional regulator
2525	-1.17	4.36E-03	*atpG*	ATP synthase subunit gamma
3285	-1.16	8.47E-05	*cpsFP*	dTDP-4-dehydrorhamnose 3,5-epimerase
9795	-1.15	5.45E-04	-	hypothetical protein
2520	-1.15	4.15E-03	*atpA*	ATP synthase subunit alpha
1040	-1.14	4.65E-05	-	peptide ABC transporter substrate-binding protein
4000	-1.14	1.42E-03	-	acid phosphatase/phosphotransferase
6695	-1.14	5.79E-05	-	tRNA (guanosine(46)-N7)-methyltransferase TrmB
1610	-1.14	1.24E-04	*mtsB*	ABC transporter ATP-binding protein
210	-1.13	8.15E-04	-	DUF368 domain-containing protein
4405	-1.13	4.74E-05	*pgmA*	phosphoglucomutase
5485	-1.12	5.59E-05	*recN*	DNA repair protein RecN
7510	-1.10	1.40E-02	*glpF.2*	aquaporin
3280	-1.10	8.18E-05	*cpsFO*	glucose-1-phosphate thymidylyltransferase
690	-1.09	1.89E-04	*mga*	transcriptional regulator
4325	-1.08	1.90E-03	-	membrane protein
2160	-1.08	4.82E-05	*bsaA*	glutathione peroxidase
105	-1.08	6.26E-04	*pgsA*	CDP-diacylglycerol-glycerol-3-phosphate 3-phosphatidyltransferase
2850	-1.07	1.14E-04	-	ABC transporter permease
3290	-1.07	8.44E-05	*cpsFQ*	dTDP-glucose 4,6-dehydratase
5880	-1.07	4.97E-03	-	carbohydrate ABC transporter permease
1910	-1.06	2.84E-03	*acpA*	ribonuclease 3
2645	-1.05	5.79E-05	*rgpBc*	alpha-L-Rha alpha-1,3-L-rhamnosyltransferase
5720	-1.05	5.05E-04	-	arginine regulator
4250	-1.05	1.10E-04	*satD*	DNA-binding protein
2480	-1.04	4.84E-05	-	nuclease
2855	-1.04	3.74E-05	-	glycerophosphodiester phosphodiesterase
2125	-1.03	6.60E-04	-	HAD family hydrolase
3780	-1.02	9.11E-05	-	iron ABC transporter substrate-binding protein
1340	-1.02	2.89E-04	-	membrane protein insertion efficiency factor YidD
4830	-1.01	2.42E-02	-	diaminopimelate epimerase
875	-1.01	2.90E-02	-	sugar ABC transporter permease
5495	-1.01	8.28E-05	*hlyA1*	TlyA family rRNA (cytidine-2'-O)-methyltransferase
4670	-1.00	4.82E-05	-	TVP38/TMEM64 family protein
4875	-1.00	4.77E-04	-	ABC transporter ATP-binding protein

*Locus_tag: identifier corresponding to reference genome sequence, prefix to all numbers listed is “SPYOHK_RS”, **logFC: log_2 fold change, ***FDR: false discovery rate.

Supplementary Table 2c. MRSA USA300 up-regulated genes following 1 h treatment with 2 µM PBT2 and 50 µM zinc in CAMHB grown at 37°C.

Locus_tag*	LogFC**	FDR***	Gene	Putative gene product function
280	8.27	2.18E-10	*frmA*	alcohol dehydrogenase
3005	6.61	3.85E-11	*vraX*	protein VraX
670	6.38	1.21E-10	*butA*	diacetyl reductase ((S)-acetoin forming)
13850	6.22	7.50E-10	-	hypothetical protein
10975	6.10	3.21E-10	*amt*	ammonia permease
11055	6.04	3.85E-11	*leuA*	2-isopropylmalate synthase
13940	6.04	7.52E-10	*ynzC*	hypothetical protein
11050	6.00	9.11E-11	*ilvC*	ketol-acid reductoisomerase
11060	5.80	1.18E-10	*leuB*	3-isopropylmalate dehydrogenase
11065	5.75	6.55E-11	*leuC*	3-isopropylmalate dehydratase large subunit
11070	5.67	4.15E-10	*leuD*	3-isopropylmalate dehydratase small subunit
13935	5.67	1.01E-09	-	unknown
11075	5.63	8.76E-11	*ilvA*	L-threonine dehydratase biosynthetic IlvA
13860	5.57	5.46E-12	*copZ*	copper chaperone CopZ
13855	5.56	1.08E-11	*zntA*	copper-exporting P-type ATPase A
11045	5.54	3.47E-09	*ilvN*	acetolactate synthase
3085	5.52	2.18E-10	-	transcriptional regulator
11040	5.32	2.91E-10	*ilvB*	acetolactate synthase, large subunit, biosynthetic type
11035	5.26	4.18E-10	*ilvD*	dihydroxy-acid dehydratase
3080	5.25	8.96E-11	-	dihydrolipoamide dehydrogenase
13600	5.23	5.87E-10	-	MarR family transcriptional regulator
4080	5.15	6.05E-10	*gap*	aldehyde dehydrogenase
13080	5.15	6.55E-11	*hlgB*	gamma-hemolysin component B
4090	5.10	3.77E-10	*tpiA*	triose-phosphate isomerase
4095	5.08	4.27E-10	*gpmI*	2,3-bisphosphoglycerate-independent phosphoglycerate mutase
4085	5.06	5.31E-10	*pgk*	phosphoglycerate kinase
13385	4.98	8.27E-10	-	carboxymuconolactone decarboxylase family protein
13075	4.89	8.96E-11	*hlgC*	gamma-hemolysin component C
13595	4.76	6.05E-10	-	MFS transporter
13730	4.73	1.72E-10	-	thiol reductase thioredoxin
4075	4.71	1.28E-09	-	transcriptional regulator
395	4.66	3.56E-11	-	copper-translocating P-type ATPase
400	4.64	1.69E-11	-	hypothetical protein
13070	4.64	1.03E-10	*hlgA*	gamma-hemolysin component A
10265	4.58	2.61E-10	-	DNA polymerase IV
4730	4.54	3.02E-09	*clpB*	chaperone protein ClpB
2725	4.50	1.98E-09	-	protein arginine kinase
4990	4.44	2.48E-09	-	hypothetical protein
11830	4.44	3.65E-10	-	NADP-dependent oxidoreductase

2715	4.40	1.69E-09	*ctsR*	transcriptional regulator CtsR
3960	4.37	1.52E-09	*yfiA*	ribosomal subunit interface protein
13170	4.34	1.03E-10	-	hypothetical protein
6480	4.33	8.54E-11	-	MerR family transcriptional regulator
6485	4.31	3.85E-11	*glnA*	glutamine synthetase
13635	4.28	8.40E-09	-	ring-cleaving dioxygenase
2730	4.27	1.37E-09	*clpC*	ATP-dependent Clp protease ATP-binding subunit ClpC
13630	4.27	1.39E-09	-	carboxylesterase
3245	4.20	2.61E-10	-	alpha/beta hydrolase
6490	4.19	1.94E-08	-	hypothetical protein
2110	4.18	6.05E-10	-	hypothetical protein
13735	4.17	1.48E-10	-	acyl-CoA thioester hydrolase
13780	4.16	4.00E-08	-	hypothetical protein
1980	4.07	5.87E-10	-	peptidase
14120	4.06	3.58E-10	-	antibiotic biosynthesis monooxygenase
11870	4.03	1.03E-10	*lacA*	unknown
340	4.00	3.83E-09	-	universal stress protein
11805	4.00	2.18E-10	*asp23*	Asp23/Gls24 family envelope stress response protein
3310	4.00	6.55E-11	-	sodium:proton antiporter
3270	3.98	4.96E-10	-	recombinase
13785	3.97	1.49E-09	-	hypothetical protein
11810	3.97	2.61E-10	-	hypothetical protein
13725	3.96	8.96E-11	-	alpha/beta hydrolase
12160	3.92	2.35E-10	-	hypothetical protein
11815	3.92	2.99E-10	-	hypothetical protein
11860	3.89	2.18E-10	*lacC*	tagatose-6-phosphate kinase
14620	3.89	3.28E-09	-	membrane protein
9425	3.89	3.67E-11	-	transaldolase
11430	3.84	2.18E-10	-	aldehyde dehydrogenase family protein
2105	3.82	2.74E-10	-	NAD(P)-dependent oxidoreductase
14615	3.82	6.44E-09	-	arylamine N-acetyltransferase
9640	3.82	5.77E-09	*epiE*	hypothetical protein
2025	3.81	1.89E-10	*ahpC*	alkyl hydroperoxide reductase subunit C
14095	3.80	2.87E-09	-	hypothetical protein
13775	3.78	1.02E-08	-	hypothetical protein
10855	3.77	9.68E-11	-	succinyl-diaminopimelate desuccinylase
11855	3.77	3.41E-10	*lacD*	tagatose 1,6-diphosphate aldolase
9645	3.72	1.66E-09	*epiF*	bacitracin ABC transporter ATP-binding protein
13750	3.71	2.38E-10	*cidB*	holin-like protein CidB
8075	3.70	1.97E-08	-	hypothetical protein
11840	3.67	2.45E-10	*lacG*	6-phospho-beta-galactosidase
13745	3.66	1.10E-10	*cidC*	pyruvate oxidase
12860	3.66	7.52E-10	-	general stress protein
4225	3.66	2.18E-10	-	hypothetical protein

7505	3.62	8.76E-11	-	hypothetical protein
5270	3.62	1.62E-09	-	hypothetical protein
2030	3.61	6.65E-11	-	NADPH-dependent oxidoreductase
3815	3.61	2.09E-10	-	lipid kinase
545	3.61	2.91E-09	-	amidohydrolase
13290	3.61	2.18E-10	-	peptidase M28
12605	3.60	2.45E-10	*hutG*	formimidoylglutamase
11930	3.60	1.34E-09	-	toxin
550	3.60	1.34E-09	-	MFS transporter
11550	3.58	1.21E-10	-	NAD(P)-dependent oxidoreductase
4400	3.55	2.09E-10	-	CsbD family protein
11850	3.54	4.97E-10	*lacF*	lactose-specific phosphotransferase enzyme IIA component
8750	3.54	7.52E-10	-	DUF4930 domain-containing protein
13145	3.48	4.96E-10	-	GtrA family protein
355	3.47	1.38E-09	-	lysophospholipase
2100	3.44	4.00E-08	-	membrane protein
10140	3.44	8.96E-10	-	glutamine amidotransferase
4065	3.42	2.84E-10	-	epimerase
1805	3.41	2.72E-10	-	NAD(P)H-dependent oxidoreductase
1795	3.39	3.03E-10	-	glyoxalase
12575	3.38	8.96E-11	-	oxidoreductase
13805	3.38	1.13E-09	-	ATP-dependent Clp protease ATP-binding subunit
12320	3.38	1.35E-10	*fdhD*	sulfurtransferase FdhD
9040	3.38	9.44E-09	-	universal stress protein UspA
4100	3.37	2.63E-09	*eno*	enolase
3305	3.34	1.54E-10	-	cation:proton antiporter
3275	3.34	1.10E-10	-	cation:proton antiporter
2020	3.34	3.01E-10	*ahpF*	alkyl hydroperoxide reductase subunit F
1800	3.34	2.35E-10	-	LLM class flavin-dependent oxidoreductase
13655	3.34	8.62E-10	*frp*	NAD(P)H-dependent oxidoreductase
2055	3.33	1.86E-10	-	hypothetical protein
8395	3.32	1.88E-08	*dnaK*	molecular chaperone DnaK
10425	3.31	2.20E-08	-	hypothetical protein
3280	3.31	1.01E-09	-	Na(+)/H(+) antiporter subunit B
10105	3.29	9.16E-10	-	metal-dependent hydrolase
14010	3.27	8.96E-10	-	fructosamine kinase family protein
13800	3.27	7.82E-08	-	methylated-DNA--[protein]-cysteine S-methyltransferase
1090	3.27	4.76E-10	-	peptidase M23
1240	3.25	7.57E-10	-	nitric oxide dioxygenase
9020	3.25	2.50E-09	-	metal-dependent hydrolase
3285	3.24	2.63E-09	-	cation:proton antiporter
8080	3.19	3.87E-09	-	mannosyl-glycoprotein endo-beta-N-acetylglucosamidase
14090	3.18	4.48E-08	-	aspartate aminotransferase family protein
1750	3.17	7.03E-10	-	aldehyde reductase

7325	3.17	2.18E-10	*nth*	endonuclease III
4060	3.16	4.18E-10	*clpP*	ATP-dependent Clp protease proteolytic subunit
1235	3.14	1.15E-07	-	hypothetical protein
3585	3.14	4.95E-10	-	hypothetical protein
4030	3.14	2.18E-10	*trxB*	thioredoxin reductase
3290	3.13	2.18E-10	-	cation:proton antiporter
7330	3.13	1.65E-10	-	chromosome replication protein DnaD
2550	3.13	4.54E-10	-	stage V sporulation protein G
14560	3.11	4.43E-09	-	polyisoprenoid-binding protein
3575	3.10	2.18E-10	-	Rossman fold protein, TIGR00730 family
3300	3.10	3.63E-10	-	cation:proton antiporter
9790	3.10	7.48E-10	-	hypothetical protein
8405	3.10	5.26E-08	*hrcA*	HrcA family transcriptional regulator
11835	3.09	2.18E-10	-	alpha/beta hydrolase
13140	3.07	8.68E-09	-	hypothetical protein
14370	3.07	1.03E-09	-	accessory Sec system translocase SecA2
3295	3.06	2.72E-08	-	Na+/H+ antiporter subunit E
1645	3.06	4.56E-10	-	peptide ABC transporter permease
9210	3.05	9.94E-10	-	DUF948 domain containing protein
7365	3.04	6.76E-10	-	zinc metallopeptidase
3730	3.04	1.72E-09	-	7-carboxy-7-deazaguanine synthase QueE
4045	3.03	2.74E-10	-	YvcK family protein
7320	3.03	1.54E-10	-	hypothetical protein
12790	3.03	5.36E-09	-	hypothetical protein
7975	3.02	1.38E-09	-	hypothetical protein
3725	3.02	3.84E-10	-	hypothetical protein
5805	3.01	1.18E-08	-	hypothetical protein
8400	2.99	2.39E-08	*grpE*	nucleotide exchange factor GrpE
1650	2.97	4.96E-10	-	ABC transporter ATP-binding protein
7045	2.95	2.72E-10	-	hypothetical protein
2545	2.94	4.75E-10	-	RidA family protein
3910	2.94	2.61E-10	-	glycerate kinase
4050	2.93	1.79E-10	-	sporulation regulator WhiA
7160	2.93	2.10E-09	-	hypothetical protein
9055	2.92	1.43E-10	*tpx*	2-Cys peroxiredoxin
7485	2.91	4.26E-10	*ebpS*	elastin-binding protein EbpS
10450	2.91	2.38E-09	-	thioredoxin family protein
7165	2.90	1.38E-09	*crr*	glucose-specific phosphotransferase enzyme IIA component
13640	2.89	1.78E-09	-	MarR family transcriptional regulator
10475	2.88	4.25E-10	-	GntR family transcriptional regulator
4635	2.88	2.45E-10	-	NADH-dependent flavin oxidoreductase
12400	2.88	9.63E-09	-	hypothetical protein
5895	2.87	2.83E-10	-	glyoxalase
4260	2.87	8.62E-10	-	thiol reductase thioredoxin

4245	2.87	4.38E-09	-	organic hydroperoxide reductase OsmC/OhrA
7615	2.87	5.40E-09	-	tail protein
3580	2.86	7.50E-10	-	DUF188 domain-containing protein
5605	2.85	2.18E-10	-	colicin V production protein CvpA
11825	2.85	6.51E-10	-	zinc-binding alcohol dehydrogenase
13370	2.84	7.28E-10	-	hypothetical protein
740	2.83	8.24E-09	*deoB*	phosphopentomutase
3735	2.82	1.43E-08	-	6-carboxytetrahydropterin synthase QueD
5735	2.82	6.71E-09	-	hypothetical protein
11615	2.80	9.42E-10	*mtlA*	PTS mannitol transporter subunit IIA
13975	2.80	5.75E-10	-	alpha/beta hydrolase
8610	2.80	2.32E-10	-	LLM class flavin-dependent oxidoreductase
9650	2.79	3.11E-09	*epiP*	peptidase S8
10900	2.78	3.07E-08	*groL*	molecular chaperone GroEL
7970	2.77	1.43E-09	*gnd*	phosphogluconate dehydrogenase (NADP(+)-dependent, decarboxylating)
5630	2.77	1.48E-10	-	excinuclease ABC subunit C
3940	2.77	7.62E-10	-	YigZ family protein
1990	2.77	2.20E-10	-	GlsB/YeaQ/YmgE family stress response membrane protein
4550	2.77	2.53E-10	-	hypothetical protein
5285	2.75	2.61E-10	-	hypothetical protein
9240	2.75	3.42E-09	-	glutamyl aminopeptidase
11530	2.74	5.17E-10	-	hypothetical protein
11620	2.74	3.16E-10	*mtlD*	mannitol-1-phosphate 5-dehydrogenase
3235	2.74	3.73E-09	-	hypothetical protein
12470	2.73	7.52E-10	-	hypothetical protein
7620	2.72	5.13E-08	-	tail protein
14355	2.71	1.44E-08	-	hypothetical protein
4295	2.67	4.86E-09	-	thiol reductase thioredoxin
12510	2.67	1.28E-09	-	MurR/RpiR family transcriptional regulator
12475	2.67	8.74E-10	-	formate dehydrogenase subunit alpha
7170	2.67	4.86E-09	*msrB*	peptide-methionine (R)-S-oxide reductase
6725	2.67	2.45E-09	-	transketolase
10470	2.64	1.04E-09	-	ABC transporter ATP-binding protein
4845	2.63	1.24E-09	-	adaptor protein MecA
10905	2.62	3.30E-08	*groES*	co-chaperone GroES
5405	2.62	2.83E-10	-	membrane protein
8795	2.61	5.56E-08	-	hypothetical protein
8745	2.61	5.14E-09	-	hypothetical protein
11775	2.58	3.83E-09	-	IucA/IucC family siderophore biosynthesis protein
14675	2.58	8.10E-10	-	nucleoid occlusion protein
13415	2.57	4.96E-10	-	hypothetical protein
11230	2.55	1.49E-08	-	hypothetical protein
8260	2.55	9.04E-10	-	superoxide dismutase
6680	2.54	1.75E-07	-	catalase

3410	2.54	9.00E-10	-	dihydroxyacetone kinase subunit DhaK
11610	2.53	6.05E-10	-	PTS lactose transporter subunit IIB
11590	2.53	1.74E-09	-	HAD family phosphatase
2280	2.52	4.47E-08	-	NADH dehydrogenase subunit 5
8790	2.52	2.56E-10	-	aminopeptidase
3700	2.52	4.62E-09	-	glycosyltransferase
7600	2.51	3.93E-09	-	phage tail tape measure protein
8615	2.51	2.74E-10	-	hypothetical protein
4290	2.49	2.06E-09	-	topiosmerase
9275	2.48	2.18E-10	-	YtxH domain-containing protein
995	2.48	6.78E-10	*ipdC*	indolepyruvate decarboxylase
13270	2.46	5.79E-09	*pnbA*	carboxylesterase/lipase family protein
705	2.45	2.92E-10	-	Superoxide dismutase [Mn/Fe] 2
10465	2.44	2.06E-09	-	ABC-2 transporter family protein
10455	2.44	2.56E-09	-	membrane protein
13685	2.44	5.69E-09	-	N-acetyltransferase
990	2.43	8.25E-10	*entB*	isochorismatase
10065	2.42	2.20E-10	-	2-hydroxyacid dehydrogenase
10070	2.41	2.23E-10	-	peroxiredoxin
5620	2.40	3.33E-10	*trx*	thiol reductase thioredoxin
8620	2.40	2.31E-09	-	CsbD family protein
4860	2.39	2.56E-10	-	hypothetical protein
725	2.39	6.30E-07	*deoD*	purine-nucleoside phosphorylase
9490	2.39	2.99E-10	-	membrane protein insertion efficiency factor YidD
730	2.39	8.89E-08	-	tetracycline efflux MFS transporter Tet(38)
7175	2.38	3.19E-09	*msrA*	peptide-methionine (S)-S-oxide reductase
6305	2.38	3.09E-10	*ribF*	bifunctional riboflavin kinase/FMN adenylyltransferase
14365	2.38	2.46E-09	-	accessory Sec system glycosyltransferase GtfA
9105	2.37	4.96E-10	-	peroxiredoxin
595	2.37	4.56E-10	*sirC*	iron ABC transporter permease
14315	2.37	1.29E-06	*isaB*	hypothetical protein
14450	2.37	1.81E-09	*tetR*	biofilm operon icaADBC HTH-type negative transcriptional regulator IcaR
7475	2.36	5.20E-10	-	thioredoxin reductase
8365	2.36	1.81E-09	-	hypothetical protein
10925	2.36	3.01E-09	-	hydrolase in agr operon
6805	2.35	3.38E-09	-	protein GlcT
3335	2.35	8.58E-09	-	membrane protein
2865	2.35	7.19E-10	-	Molecular chaperone Hsp31 and glyoxalase 3
8360	2.33	8.74E-10	-	hypothetical protein
12620	2.33	1.21E-09	-	MOSC domain-containing protein
7940	2.33	2.74E-10	*zwf*	glucose-6-phosphate dehydrogenase
8355	2.32	1.09E-09	-	hypothetical protein
13420	2.32	4.00E-08	-	hypothetical protein
10875	2.30	1.66E-09	-	N-acetyltransferase

9770	2.30	1.44E-09	-	signal transduction protein TRAP
4425	2.29	3.01E-10	-	iron-sulfur cluster assembly scaffold protein NifU
9875	2.29	5.10E-10	-	glucosamine-6-phosphate isomerase
7295	2.29	1.22E-09	-	hypothetical protein
2285	2.28	1.22E-07	-	hypothetical protein
9870	2.28	6.51E-10	-	hypothetical protein
4415	2.28	3.58E-10	*sufD*	Fe-S cluster assembly protein SufD
14415	2.28	5.75E-10	-	hypothetical protein
11560	2.27	4.13E-10	*czcD*	cation transporter
10390	2.26	1.81E-09	-	pectate lyase
3450	2.26	1.43E-09	-	N-acetyltransferase
8385	2.26	3.71E-08	*prmA*	50S ribosomal protein L11 methyltransferase
8390	2.26	4.83E-08	*dnaJ*	molecular chaperone DnaJ
220	2.26	5.28E-08	-	hypothetical protein
12285	2.26	1.96E-09	*moeA*	molybdopterin molybdenumtransferase
9455	2.25	2.82E-10	-	aldo/keto reductase
8880	2.25	8.12E-10	*lysP*	gamma-aminobutyrate permease
14630	2.25	4.87E-07	-	ABC transporter permease
9235	2.25	3.52E-09	-	thiol reductase thioredoxin
3415	2.24	1.43E-09	-	dihydroxyacetone kinase subunit L
6060	2.24	2.99E-10	*recG*	DNA helicase RecG
12560	2.23	3.64E-08	-	hypothetical protein
4865	2.22	1.55E-09	-	hypothetical protein
14350	2.22	1.26E-08	-	cysteine hydrolase
3955	2.22	3.29E-07	-	competence protein ComF
5795	2.21	1.15E-08	-	hemolytic protein
5965	2.20	3.20E-10	-	hypothetical protein
4430	2.20	3.03E-10	*sufB*	Fe-S cluster assembly protein SufB
5790	2.20	5.80E-09	-	hemolytic protein
5810	2.19	4.43E-09	-	bacillithiol biosynthesis cysteine-adding enzyme BshC
10460	2.19	1.88E-09	-	ABC transporter ATP-binding protein
560	2.19	1.07E-07	-	oleate hydratase
13970	2.19	6.43E-10	-	amidohydrolase
12490	2.18	1.43E-09	-	YafY family transcriptional regulator
4555	2.18	5.45E-10	-	iron-sulfur cluster assembly accessory protein
575	2.17	1.55E-07	-	hypothetical protein
2955	2.16	2.33E-09	-	glucosamine-6-phosphate deaminase
6365	2.15	6.78E-10	*cinA*	competence protein CinA
6380	2.13	1.72E-09	-	hypothetical protein
8925	2.12	1.54E-09	*polA*	DNA polymerase I
6815	2.12	6.78E-10	-	AI-2E family transporter
7235	2.12	2.39E-09	-	hyperosmolarity resistance protein Ebh
12675	2.11	4.19E-09	-	3-hydroxyacyl-CoA dehydrogenase
4040	2.11	4.96E-10	-	RNase adaptor protein RapZ

1415	2.11	5.37E-09	-	ribose transporter RbsU
4410	2.10	3.68E-10	*sufC*	ABC transporter ATP-binding protein
6200	2.09	2.33E-09	*hslV*	HslU--HslV peptidase proteolytic subunit
7300	2.09	7.29E-09	-	hypothetical protein
6685	2.08	5.89E-07	-	50S ribosomal protein L33
6630	2.08	1.81E-09	-	hypothetical protein
8915	2.07	1.78E-09	*coaE*	dephospho-CoA kinase
11500	2.07	3.71E-08	-	membrane protein
10260	2.05	6.68E-09	-	DNA polymerase III subunit epsilon
12260	2.04	4.96E-10	*moaA*	cyclic pyranopterin monophosphate synthase
6215	2.04	2.20E-07	-	hypothetical protein
12280	2.04	1.04E-09	*mobB*	molybdopterin-guanine dinucleotide biosynthesis protein B
9325	2.03	1.65E-08	*rot*	MarR family transcriptional regulator
10170	2.02	3.45E-09	-	protein-tyrosine-phosphatase
7060	2.02	6.25E-10	-	tellurite resistance protein TelA
5980	2.01	4.96E-10	*coaBC*	phosphopantothenoylcysteine decarboxylase
3420	2.01	1.92E-08	-	PTS-dependent dihydroxyacetone kinase phosphotransferase subunit DhaM
12265	2.01	2.73E-09	*mobA*	molybdenum cofactor guanylyltransferase
6465	2.00	8.62E-10	-	glutathione peroxidase
8920	2.00	1.34E-09	*mutM*	DNA-formamidopyrimidine glycosylase
4700	2.00	1.19E-08	-	hypothetical protein
485	1.99	5.21E-08	-	LysR family transcriptional regulator
900	1.98	3.12E-08	-	cation transporter
4670	1.98	3.12E-09	*pgi*	glucose-6-phosphate isomerase
6765	1.98	9.14E-09	*acnA*	aconitate hydratase
14075	1.97	2.61E-08	-	L-lactate dehydrogenase
12420	1.96	2.81E-08	-	hypothetical protein
7355	1.96	1.74E-09	-	N-acetyl-alpha-D-glucosaminyl L-malate synthase BshA
14605	1.96	7.44E-08	-	DNA-binding protein
6195	1.95	5.96E-09	*xerC*	tyrosine recombinase XerC
12940	1.94	2.03E-07	-	uroporphyrinogen-III C-methyltransferase
12680	1.93	1.81E-09	-	hypothetical protein
4445	1.93	3.79E-09	-	HlyC/CorC family transporter
14360	1.92	1.31E-08	-	accessory Sec system glycosylation chaperone GtfB
5290	1.91	1.81E-09	*ptsH*	phosphocarrier protein HPr
3530	1.91	1.24E-09	-	LysR family transcriptional regulator
2350	1.90	3.01E-09	-	hypothetical protein
6210	1.90	8.49E-09	*codY*	GTP-sensing pleiotropic transcriptional regulator CodY
11950	1.89	2.12E-07	-	hypothetical protein
13280	1.89	3.41E-08	-	iron export ABC transporter permease subunit FetB
6205	1.88	2.69E-09	*hslU*	HslU--HslV peptidase ATPase subunit
8955	1.88	2.03E-09	*gltA*	citrate synthase
7050	1.88	5.23E-09	-	acylphosphatase
10175	1.88	8.62E-10	-	hypothetical protein

6810	1.88	1.99E-07	-	hypothetical protein
14100	1.87	7.52E-10	-	class I fructose-bisphosphate aldolase
8950	1.87	5.27E-09	*icd*	isocitrate dehydrogenase (NADP(+))
3250	1.87	7.61E-09	*sarA*	transcriptional regulator
5010	1.86	2.00E-06	-	putative holin-like toxin
12550	1.86	6.95E-09	*glvC*	PTS alpha-glucoside transporter subunit IIBC
3525	1.85	2.19E-09	-	hypothetical protein
4695	1.85	2.89E-09	*rexA*	ATP-dependent helicase/nuclease subunit A
14690	1.84	4.46E-09	*trmE*	tRNA uridine-5-carboxymethylaminomethyl(34) synthesis GTPase MnmE
9205	1.84	9.63E-09	-	membrane protein
4165	1.84	2.14E-09	*clfA*	clumping factor A
14685	1.84	2.77E-09	*gidA*	tRNA uridine-5-carboxymethylaminomethyl(34) synthesis enzyme MnmG
7985	1.82	1.43E-09	-	membrane protein
5295	1.82	2.10E-09	*ptsI*	phosphoenolpyruvate--protein phosphotransferase
13260	1.82	2.24E-09	-	hypothetical protein
7445	1.82	9.67E-09	*rpsA*	30S ribosomal protein S1
4690	1.82	2.81E-09	*rexB*	ATP-dependent helicase/deoxyribonuclease subunit B
13845	1.81	9.50E-09	-	maltose O-acetyltransferase
1410	1.81	8.58E-09	*rbsD*	D-ribose pyranase
2965	1.81	4.26E-09	-	6-phospho-3-hexuloisomerase
14115	1.81	9.94E-10	-	acetyl-CoA synthetase
14680	1.81	7.29E-09	*gidB*	ribosomal RNA small subunit methyltransferase G
13285	1.80	4.17E-09	-	ABC transporter ATP-binding protein
7360	1.80	3.62E-09	-	hypothetical protein
5395	1.80	5.91E-08	*potD*	spermidine/putrescine ABC transporter substrate-binding protein
11555	1.80	1.43E-09	*arsR*	transcriptional regulator
6590	1.79	1.96E-07	-	hypothetical protein
12295	1.78	1.67E-08	*moaB*	molybdenum cofactor biosynthesis protein
2960	1.77	5.93E-09	-	3-hexulose-6-phosphate synthase
6690	1.76	6.27E-07	*rpmN*	30S ribosomal protein S14
5365	1.75	4.25E-09	*lpdA*	dihydrolipoyl dehydrogenase
11905	1.75	1.42E-07	-	MerR family transcriptional regulator
5275	1.75	2.35E-09	-	hypothetical protein
5355	1.75	5.32E-09	*pdhB*	pyruvate dehydrogenase E1 component subunit beta
3495	1.75	1.55E-08	-	hypothetical protein
2290	1.74	7.23E-09	-	hypothetical protein
13765	1.74	1.06E-08	-	hypothetical protein
4885	1.73	9.44E-09	-	NAD(+) kinase
13680	1.73	2.06E-09	*srtA*	class A sortase SrtA
225	1.72	1.42E-08	-	ATPase AAA
2050	1.72	1.97E-09	-	hypothetical protein
12485	1.72	3.33E-08	-	inositol monophosphatase
5070	1.71	1.60E-08	-	acetyltransferase

3965	1.71	1.04E-08	*secA*	preprotein translocase subunit SecA
12800	1.70	4.92E-09	-	quinone oxidoreductase
5420	1.70	7.32E-08	-	inositol monophosphatase
1405	1.69	3.58E-09	*rbsK*	ribokinase
14590	1.69	5.77E-09	-	DinB family protein
10060	1.69	4.18E-08	-	transcriptional repressor
13570	1.69	6.82E-09	-	hypothetical protein
7350	1.68	2.31E-09	-	CCA-adding enzyme
9365	1.68	6.68E-04	*ribE*	riboflavin synthase
735	1.67	2.73E-07	*deoC*	2-deoxyribose-5-phosphate aldolase
8835	1.67	1.71E-09	*clpX*	ATP-dependent Clp protease ATP-binding subunit ClpX
12960	1.67	6.44E-07	-	N-acetyltransferase
4195	1.66	1.27E-07	-	hypothetical protein
12950	1.66	2.01E-07	*nirB*	nitrite reductase large subunit
5350	1.65	6.15E-09	*pdhA*	pyruvate dehydrogenase E1 component subunit alpha
7115	1.65	4.33E-09	*arlR*	DNA-binding response regulator
13650	1.65	2.29E-08	-	glyoxalase
13965	1.64	1.18E-07	-	short-chain dehydrogenase
5360	1.64	2.80E-09	-	dihydrolipoyllysine-residue acetyltransferase component of pyruvate dehydrogenase complex
3630	1.63	1.07E-07	-	deoxyribodipyrimidine photo-lyase
13740	1.63	6.69E-08	*ptsG*	PTS glucoside EIICBA component
3535	1.63	1.64E-07	-	hypothetical protein
13410	1.63	5.10E-09	-	oxidoreductase
6165	1.63	3.73E-09	*sucD*	succinyl-CoA ligase subunit alpha
945	1.62	3.10E-08	-	MFS transporter
13205	1.62	3.72E-09	-	protein flp
9250	1.61	5.14E-09	-	MBL fold metallo-hydrolase
4070	1.61	2.86E-08	-	DUF4887 domain-containing protein
12275	1.61	5.24E-09	*moaE*	molybdopterin synthase catalytic subunit
2190	1.60	8.35E-08	-	hypothetical protein
11310	1.59	1.71E-09	*fabZ*	beta-hydroxyacyl-ACP dehydratase
4640	1.59	1.73E-08	*rocD*	Ornithine aminotransferase 2
4250	1.59	1.47E-08	*aroD*	3-dehydroquinase
65	1.59	4.48E-09	-	homoserine O-acetyltransferase
13645	1.58	6.11E-07	-	N-acetyltransferase
7990	1.58	1.32E-08	-	hypothetical protein
5650	1.58	1.41E-08	*murI*	glutamate racemase
11900	1.58	9.36E-09	-	aldo/keto reductase
1310	1.56	3.70E-09	-	2-C-methyl-D-erythritol 4-phosphate cytidylyltransferase
13960	1.56	7.54E-07	-	TetR family transcriptional regulator
5610	1.55	1.70E-08	-	DNA polymerase/3'-5' exonuclease PolX
3375	1.55	4.36E-09	-	ABC transporter ATP-binding protein
3565	1.53	1.37E-08	-	hypothetical protein
4645	1.53	1.43E-08	*gudB*	NAD-specific glutamate dehydrogenase

11320	1.53	8.36E-09	-	membrane protein
12730	1.52	2.22E-08	*tcaR*	transcriptional regulator
12270	1.51	4.59E-06	*moaD*	molybdopterin synthase sulfur carrier subunit
14125	1.51	1.76E-06	-	hypothetical protein
6230	1.51	1.41E-08	*tsf*	elongation factor Ts
3065	1.51	5.63E-09	*mvaD*	diphosphomevalonate decarboxylase
13615	1.50	4.35E-09	-	fructose 1,6-bisphosphatase
4995	1.49	8.55E-09	-	lipoate--protein ligase A
7695	1.49	2.39E-07	-	hypothetical protein
9340	1.49	8.49E-09	-	alpha/beta hydrolase
11770	1.48	2.73E-07	-	alanine racemase
6375	1.48	4.34E-09	-	ribonuclease Y
7945	1.48	4.26E-09	-	AraC family transcriptional regulator
6850	1.48	3.21E-08	-	aminopeptidase
5190	1.48	8.28E-08	*folD*	bifunctional methylenetetrahydrofolate dehydrogenase/methenyltetrahydrofolate cyclohydrolase
4380	1.48	3.22E-08	-	phage capsid protein
9185	1.47	3.31E-09	*ccpA*	catabolite control protein A
7090	1.47	2.13E-07	-	hypothetical protein
7590	1.46	7.89E-06	-	peptidase
6905	1.46	1.28E-07	-	SWIM zinc finger family protein
9360	1.46	1.12E-03	*ribBA*	riboflavin biosynthesis protein RibBA
4715	1.46	6.31E-09	-	HAD family phosphatase
8975	1.45	2.54E-07	*pyk*	pyruvate kinase
7095	1.45	8.45E-08	-	hypothetical protein
3545	1.45	1.14E-08	-	membrane protein
7515	1.45	1.87E-07	-	hypothetical protein
12300	1.45	3.77E-08	*moeB*	molybdopterin biosynthesis protein MoeB
11705	1.44	2.97E-09	-	chromosome partitioning protein ParA
11525	1.44	9.83E-05	*dps*	DNA starvation/stationary phase protection protein
13495	1.44	9.78E-08	-	MFS transporter
2380	1.44	1.28E-07	*gltB*	glutamate synthase large subunit
4880	1.43	3.50E-08	-	GTP pyrophosphokinase
7345	1.42	6.44E-09	*birA*	bifunctional biotin--[acetyl-CoA-carboxylase] synthetase/biotin operon repressor
2395	1.42	2.06E-04	*treP*	PTS ascorbate transporter subunit IIA
175	1.42	1.30E-07	-	type I restriction endonuclease
10580	1.41	1.69E-06	-	hypothetical protein
5095	1.41	9.30E-09	*menB*	1,4-dihydroxy-2-naphthoyl-CoA synthase
8085	1.41	8.47E-08	-	membrane protein
3540	1.40	2.14E-08	-	MFS transporter
8335	1.40	1.45E-07	*cdd*	cytidine deaminase
1050	1.40	6.28E-08	-	RES domain-containing protein
5440	1.39	7.29E-09	-	hypothetical protein
7290	1.39	4.29E-08	-	cell cycle protein GpsB

8340	1.39	1.64E-07	*dgkA*	diacylglycerol kinase
4005	1.39	2.99E-08	*uvrA*	excinuclease ABC subunit A
5320	1.39	1.59E-07	-	hypothetical protein
14030	1.38	1.60E-07	-	VOC family protein
12775	1.37	3.73E-08	*mqo*	malate:quinone oxidoreductase
8440	1.37	1.87E-08	-	ComE operon protein 2
10075	1.37	6.42E-09	*hemL*	glutamate-1-semialdehyde 2,1-aminomutase
8310	1.36	7.68E-08	-	phosphoenolpyruvate synthetase regulatory protein
8150	1.36	3.70E-07	-	membrane protein
7100	1.36	2.13E-08	*sucB*	dihydrolipoyllysine-residue succinyltransferase component of 2-oxoglutarate dehydrogenase complex
6160	1.36	2.44E-08	*sucC*	succinyl-CoA ligase subunit beta
13210	1.36	3.52E-08	-	NAD(P)-dependent oxidoreductase
12365	1.36	3.05E-07	*ureC*	urease subunit alpha
9355	1.35	1.04E-03	*ribH*	6,7-dimethyl-8-ribityllumazine synthase
9550	1.35	8.12E-06	-	hypothetical protein
14600	1.35	4.31E-08	*rarD*	protein RarD
14580	1.35	1.01E-06	-	hypothetical protein
5085	1.35	2.44E-08	*menD*	2-succinyl-5-enolpyruvyl-6-hydroxy-3- cyclohexene-1-carboxylate synthase
2975	1.35	2.58E-08	-	proline/betaine transporter
14035	1.34	2.24E-07	-	TetR/AcrR family transcriptional regulator
7105	1.34	5.35E-08	*sucA*	2-oxoglutarate dehydrogenase E1 component
1890	1.33	3.35E-08	-	metal-dependent hydrolase
170	1.32	6.18E-08	-	beta-lactam sensor/signal transducer MecR1
5615	1.32	4.06E-08	*mutS2*	endonuclease MutS2
4970	1.32	3.45E-08	-	bifunctional metallophosphatase/5'-nucleotidase
3070	1.32	6.66E-09	-	phosphomevalonate kinase
3240	1.32	3.83E-08	-	hypothetical protein
3650	1.31	5.57E-08	*norA*	multidrug efflux MFS transporter NorA
12985	1.31	1.06E-06	-	Txe/YoeB family addiction module toxin
5315	1.31	4.77E-08	*trkA*	potassium transporter Trk
8980	1.31	3.52E-07	*pfkA*	ATP-dependent 6-phosphofructokinase
3675	1.31	4.47E-06	*fruA*	PTS fructose transporter subunit IIC
4775	1.31	6.71E-08	*fabF*	beta-ketoacyl-[acyl-carrier-protein] synthase II
4240	1.31	3.25E-07	-	N-acetyltransferase
7110	1.29	1.55E-08	*arlS*	two-component sensor histidine kinase
8350	1.28	6.26E-08	*phoH*	PhoH family protein
5985	1.27	1.44E-08	*priA*	primosomal protein N'
8345	1.27	9.58E-08	-	endoribonuclease YbeY
9800	1.27	1.51E-08	*prsA*	foldase
3720	1.27	5.21E-08	-	hypothetical protein
5435	1.26	4.77E-08	-	DUF2197 domain-containing protein
10870	1.26	1.78E-06	-	potassium transporter KtrB
3595	1.26	3.18E-08	-	cysteine ABC transporter ATP-binding protein

10135	1.26	5.97E-08	*sgtB*	monofunctional glycosyltransferase
3600	1.25	7.44E-08	-	cysteine ABC transporter ATP-binding protein
5655	1.25	2.94E-07	-	non-canonical purine NTP pyrophosphatase
4935	1.25	1.13E-08	-	diacylglycerol beta-glucosyltransferase
10755	1.25	4.93E-07	-	ATPase
12220	1.24	1.29E-06	-	hypothetical protein
10095	1.24	2.20E-08	-	hypothetical protein
12380	1.24	2.71E-07	*ureG*	urease accessory protein UreG
6825	1.24	2.49E-08	*msrA*	peptide-methionine (S)-S-oxide reductase
8070	1.23	1.23E-06	-	hypothetical protein
8145	1.23	5.30E-08	-	hypothetical protein
10750	1.23	8.52E-07	-	recombinase
1970	1.23	2.79E-07	-	toxin
11315	1.22	1.00E-08	*murA*	UDP-N-acetylglucosamine 1-carboxyvinyltransferase
9085	1.22	1.74E-08	-	hypothetical protein
12375	1.21	3.45E-07	*ureF*	urease accessory protein UreF
11595	1.21	3.89E-07	-	iron ABC transporter ATP-binding protein
12370	1.20	8.80E-06	*ureE*	urease accessory protein UreE
14420	1.20	5.44E-07	-	hypothetical protein
10130	1.20	8.47E-08	-	regulatory protein RecX
10410	1.19	4.08E-08	*aldA2*	aldehyde dehydrogenase
5090	1.19	2.59E-08	-	2-succinyl-6-hydroxy-2, 4-cyclohexadiene-1-carboxylate synthase
7190	1.19	3.18E-08	*thyA*	thymidylate synthase
5300	1.19	3.26E-07	-	NrdH-redoxin
9400	1.19	5.72E-08	-	hypothetical protein
13870	1.18	3.68E-08	-	N-succinyldiaminopimelate aminotransferase
11505	1.18	1.42E-06	*pdp*	pyrimidine-nucleoside phosphorylase
14020	1.18	3.99E-06	-	hypothetical protein
12850	1.18	1.85E-07	-	hypothetical protein
10785	1.17	3.92E-06	-	oxidoreductase
45	1.17	6.59E-08	*hutH*	histidine ammonia-lyase
4925	1.17	2.39E-06	-	hypothetical protein
6320	1.16	2.81E-08	-	ribonuclease J 2
445	1.16	8.66E-07	-	MBL fold metallo-hydrolase
4875	1.14	5.90E-08	-	hypothetical protein
4200	1.14	1.35E-07	-	hypothetical protein
7950	1.14	2.56E-07	-	alpha-glucosidase
12385	1.14	3.37E-07	*ureD*	urease accessory protein
8095	1.13	7.87E-07	-	ATPase AAA
13890	1.13	4.54E-07	-	diapolycopene oxygenase
4545	1.11	2.04E-06	-	NADH dehydrogenase family protein
3345	1.11	2.34E-08	*tagH*	teichoic acids export ATP-binding protein TagH
13175	1.11	6.23E-07	-	phosphohydrolase
2385	1.11	2.16E-05	*gltD*	glutamate synthase subunit beta

3190	1.10	1.75E-06	*adh*	zinc-dependent alcohol dehydrogenase
4120	1.10	6.05E-08	*rnr*	ribonuclease R
4115	1.10	3.45E-08	*est*	carboxylesterase
25	1.09	5.32E-07	*recF*	DNA replication and repair protein RecF
10480	1.09	7.78E-05	-	hypothetical protein
6945	1.09	3.71E-05	*phoU*	phosphate transport system regulatory protein PhoU
3460	1.08	7.30E-08	-	DNA-binding response regulator
4000	1.08	6.25E-07	*uvrB*	excinuclease ABC subunit B
3695	1.08	6.60E-07	-	aldo/keto reductase
12990	1.08	5.32E-06	-	prevent-host-death protein
8800	1.06	4.61E-07	*hemL*	glutamate-1-semialdehyde 2,1-aminomutase
2195	1.05	4.13E-07	-	hypothetical protein
13885	1.05	2.43E-07	-	4,4'-diaponeurosporenoate glycosyltransferase
8805	1.05	6.15E-07	*hemB*	delta-aminolevulinic acid dehydratase
30	1.05	4.48E-07	*gyrB*	DNA gyrase subunit B
8455	1.04	3.00E-07	-	ribosome silencing factor RsfS
13310	1.04	1.14E-06	-	hypothetical protein
5485	1.04	3.68E-07	-	regulator
1335	1.03	7.57E-07	-	ribitol-5-phosphate dehydrogenase
2935	1.03	2.68E-06	-	glycosyl transferase family 1
11710	1.03	1.90E-07	-	MFS transporter
6710	1.02	5.21E-06	*lexA*	LexA repressor
7540	1.02	2.12E-07	*lukF-PV*	beta-channel forming cytolysin
5480	1.02	1.52E-06	-	hypothetical protein
3455	1.02	7.91E-08	-	NAD(P)-dependent oxidoreductase
1985	1.01	4.15E-07	-	transcriptional regulator
2240	1.01	2.76E-05	-	tandem-type lipoprotein
310	1.01	1.84E-07	*arcC*	carbamate kinase
4370	1.00	1.16E-05	-	hypothetical protein
710	1.00	4.12E-05	-	hypothetical protein

*Locus_tag: identifier corresponding to reference genome sequence, prefix to all numbers listed is “SAUSA300_RS”, **logFC: log_2 fold change, ***FDR: false discovery rate.

Supplementary Table 2d. MRSA USA300 down-regulated genes following 1 h treatment with 2 μM PBT2 and 50 μM zinc in CAMHB grown at 37°C.

Locus_tag*	LogFC**	FDR***	Gene	Putative gene product function
3325	-7.80	3.55E-12	*znuC*	phosphonate ABC transporter ATP-binding protein
3320	-7.46	3.55E-12	*znuB*	metal ABC transporter permease

915	-7.43	2.18E-10	*tauB*	sulfonate ABC transporter ATP-binding protein
3315	-7.30	5.46E-12	*znuA*	metal ABC transporter substrate-binding protein
5540	-6.61	3.16E-10	*isdA*	iron-regulated surface determinant protein A
1635	-6.36	2.18E-10	-	5'-nucleotidase, lipoprotein e(P4) family
920	-6.32	9.59E-11	-	hypothetical protein
925	-6.27	6.44E-10	-	ABC transporter permease
2060	-6.22	6.55E-11	*xpt*	xanthine phosphoribosyltransferase
910	-6.20	2.18E-10	-	hypothetical protein
5050	-5.38	2.92E-10	-	hypothetical protein
1790	-5.34	8.76E-11	*glpT*	glycerol-3-phosphate transporter
1510	-5.28	5.87E-10	-	protein EssB
2065	-5.20	1.15E-11	*pbuX*	xanthine permease
4185	-5.04	6.55E-11	*nuc*	thermonuclease
13355	-4.88	3.82E-10	-	hypothetical protein
605	-4.83	1.12E-08	*sirA*	iron ABC transporter substrate-binding protein
5550	-4.67	2.23E-08	-	hypothetical protein
1495	-4.58	2.09E-10	-	type VII secretion protein EsaA
1470	-4.48	2.18E-10	-	hypothetical protein
11760	-4.48	3.52E-09	-	ABC transporter substrate-binding protein
7830	-4.47	5.75E-10	-	hypothetical protein
5545	-4.46	1.24E-08	-	iron-regulated surface determinant protein C
12500	-4.42	1.55E-10	-	CPBP family intramembrane metalloprotease
1515	-4.40	1.89E-10	-	protein EssC
6435	-4.28	2.03E-10	*glpF*	aquaporin family protein
4985	-4.21	6.99E-10	*comK*	competence protein ComK
12155	-4.18	1.14E-09	*rpsJ*	30S ribosomal protein S10
12645	-4.12	1.10E-09	-	sodium ABC transporter ATP-binding protein
5915	-4.08	1.58E-09	*pyrR*	bifunctional pyrimidine operon transcriptional regulator/uracil phosphoribosyltransferase
9520	-4.06	3.31E-09	-	hypothetical protein
13365	-4.04	3.09E-09	-	diaminopimelate epimerase
11755	-4.02	1.04E-08	-	iron ABC transporter permease
3435	-4.02	1.43E-09	-	alpha/beta hydrolase
3825	-4.01	1.21E-09	-	MFS transporter
8910	-4.00	1.43E-10	*gap*	aldehyde dehydrogenase
13335	-3.99	2.41E-09	-	ABC transporter ATP-binding protein
1025	-3.92	9.48E-11	-	MurR/RpiR family transcriptional regulator
10300	-3.92	2.35E-10	*gatC*	Asp-tRNA(Asn)/Glu-tRNA(Gln) amidotransferase GatCAB subunit C
6440	-3.91	1.21E-10	*glpK*	glycerol kinase
1065	-3.91	9.16E-09	-	ABC transporter permease
1020	-3.89	1.86E-10	-	permease
4220	-3.88	3.46E-09	-	hypothetical protein
7075	-3.88	5.43E-10	*brnQ*	branched-chain amino acid transport system II carrier protein
1680	-3.87	2.10E-09	*nanA*	N-acetylneuraminate lyase

12150	-3.87	4.05E-10	*rplC*	50S ribosomal protein L3
1500	-3.84	5.55E-09	-	protein EssA
930	-3.84	2.18E-10	-	acyl-CoA dehydrogenase
2180	-3.82	7.52E-10	-	restriction endonuclease subunit S
1015	-3.81	6.39E-10	-	N-acetylmuramic acid 6-phosphate etherase
1875	-3.80	9.02E-10	*ltrA*	hypothetical protein
1465	-3.75	2.36E-09	-	ABC transporter permease
5375	-3.74	2.07E-09	-	XRE family transcriptional regulator
12415	-3.74	7.80E-10	*ssaA*	CHAP domain-containing protein
14175	-3.73	4.47E-10	*nrdD*	anaerobic ribonucleoside-triphosphate reductase
7835	-3.72	5.87E-10	-	hypothetical protein
12145	-3.72	3.60E-09	*rplD*	50S ribosomal protein L4
4320	-3.71	6.05E-10	*sek*	exotoxin
12140	-3.70	1.88E-09	*rplW*	50S ribosomal protein L23
12555	-3.69	5.52E-10	-	MurR/RpiR family transcriptional regulator
7405	-3.64	1.45E-10	*ndk*	nucleoside-diphosphate kinase
3120	-3.64	9.28E-09	-	hypothetical protein
1170	-3.63	1.81E-09	-	glycerophosphoryl diester phosphodiesterase
3115	-3.62	9.53E-10	-	DUF443 domain-containing protein
12440	-3.61	2.04E-06	*ssaA*	CHAP domain-containing protein
13360	-3.61	1.07E-08	-	hypothetical protein
600	-3.60	2.45E-09	*sirB*	iron ABC transporter permease
7825	-3.60	4.96E-09	-	transcriptional regulator
8540	-3.58	3.11E-09	-	allophanate hydrolase
3140	-3.57	7.85E-09	-	DUF443 domain-containing protein
12660	-3.56	1.10E-08	*gltS*	sodium/glutamate symporter
2070	-3.55	2.12E-10	*guaB*	IMP dehydrogenase
10530	-3.53	2.11E-08	*chs*	chemotaxis inhibitory protein
12130	-3.52	2.97E-09	*rpsS*	30S ribosomal protein S19
3850	-3.51	4.44E-09	-	ribonucleotide-diphosphate reductase subunit beta
6565	-3.50	1.08E-08	-	hypothetical protein
3125	-3.48	5.40E-09	-	hypothetical protein
12125	-3.48	1.09E-08	*rplV*	50S ribosomal protein L22
1520	-3.47	8.87E-07	-	protein EsaC
12115	-3.46	1.60E-09	*rplP*	50S ribosomal protein L16
8535	-3.46	3.00E-09	-	allophanate hydrolase
4510	-3.46	6.73E-09	-	teichoic acid D-Ala incorporation-associated protein DltX
1675	-3.45	1.61E-09	-	hypothetical protein
2925	-3.45	8.62E-10	*sdrE*	MSCRAMM family adhesin SdrE
1685	-3.44	9.00E-10	-	ROK family protein
13180	-3.44	1.89E-10	-	alanine glycine permease
860	-3.40	3.02E-09	*cap5L*	glycosyltransferase WbuB
12110	-3.40	1.88E-09	*rpmC*	50S ribosomal protein L29
9435	-3.39	1.37E-08	-	camphor resistance protein CrcB

12120	-3.37	1.81E-09	*rpsC*	30S ribosomal protein S3
12105	-3.37	5.17E-10	*rpsQ*	30S ribosomal protein S17
9415	-3.35	9.15E-09	-	hypothetical protein
12100	-3.34	4.96E-10	*rplN*	50S ribosomal protein L14
13915	-3.34	2.21E-08	*isaA*	transglycosylase IsaA
12870	-3.34	1.26E-09	*gltT*	proton/sodium-glutamate symport protein GltT
9620	-3.33	3.93E-09	*splA*	serine protease SplA
13330	-3.33	3.26E-08	-	ABC transporter ATP-binding protein
8520	-3.33	3.16E-09	-	hypothetical protein
2075	-3.32	1.03E-10	*guaA*	GMP synthase (glutamine-hydrolyzing)
4515	-3.32	2.00E-10	*dltA*	D-alanine--poly(phosphoribitol) ligase
130	-3.31	3.02E-09	-	multifunctional 2',3'-cyclic-nucleotide 2'-phosphodiesterase/5'-nucleotidase/3'-nucleotidase
12095	-3.31	3.20E-10	*rplX*	50S ribosomal protein L24
9525	-3.29	4.95E-08	-	hypothetical protein
5780	-3.29	1.46E-08	-	hypothetical protein
6250	-3.29	3.52E-10	*cdsA*	phosphatidate cytidylyltransferase
4570	-3.28	4.72E-09	-	sodium:proton antiporter
1720	-3.26	1.05E-07	-	hypothetical protein
8525	-3.24	4.96E-10	*accC*	acetyl-CoA carboxylase biotin carboxylase subunit
5200	-3.24	4.39E-07	*purK*	5-(carboxyamino)imidazole ribonucleotide synthase
855	-3.24	5.96E-09	*cap5K*	capsular polysaccharide biosynthesis protein
12090	-3.22	4.45E-10	*rplE*	50S ribosomal protein L5
9600	-3.21	3.24E-08	*splE*	serine protease SplE
7020	-3.21	4.69E-09	*alr2*	alanine racemase 2
2710	-3.19	1.86E-10	*nupC*	pyrimidine nucleoside transporter NupC
2320	-3.19	1.88E-07	*cysM*	cysteine synthase
13900	-3.18	1.28E-09	-	CHAP domain-containing protein
13340	-3.16	1.73E-08	-	nickel ABC transporter permease
5380	-3.15	8.96E-09	*potA*	spermidine/putrescine import ATP-binding protein PotA
5570	-3.14	2.14E-06	-	monooxygenase IsdI
9630	-3.14	6.75E-09	-	hypothetical protein
10295	-3.14	3.03E-10	*gatA*	Asp-tRNA(Asn)/Glu-tRNA(Gln) amidotransferase GatCAB subunit A
8530	-3.14	9.44E-09	*accB*	acetyl-CoA carboxylase biotin carboxyl carrier protein subunit
14180	-3.13	1.88E-09	-	citrate transporter
9835	-3.13	2.00E-08	-	transcriptional regulator
5210	-3.12	6.11E-08	*purS*	phosphoribosylformylglycinamidine synthase
865	-3.12	3.61E-09	*cap5M*	sugar transferase
11740	-3.10	1.08E-09	-	hypothetical protein
5990	-3.10	6.91E-10	-	hypothetical protein
835	-3.09	5.06E-08	*cap5G*	UDP-N-acetylglucosamine 2-epimerase (non-hydrolyzing)
10980	-3.08	1.82E-08	-	oxidoreductase
9465	-3.08	2.14E-08	*metK*	S-adenosylmethionine synthase
14525	-3.08	2.98E-08	-	ATP phosphoribosyltransferase regulatory subunit

6760	-3.07	3.20E-10	*opuD*	glycine/betaine ABC transporter
1770	-3.07	4.79E-09	-	hypothetical protein
5555	-3.06	1.50E-07	-	heme uptake system protein IsdE
7840	-3.06	9.14E-10	-	hypothetical protein
1360	-3.03	1.13E-09	-	DNA-binding response regulator
840	-3.02	1.23E-08	*cap5H*	O-acetyltransferase
4655	-3.01	1.14E-08	*glpQ*	glycerophosphodiester phosphodiesterase
6335	-3.01	2.50E-10	-	peptidase M16
1810	-3.01	1.12E-09	-	membrane protein
3370	-3.00	5.34E-08	*pbp4*	D-alanyl-D-alanine carboxypeptidase
1695	-3.00	1.18E-09	-	N-acetylmannosamine-6-phosphate 2-epimerase
870	-2.97	2.55E-09	*cap5N*	UDP-glucose 4-epimerase
830	-2.97	2.98E-08	*cap5F*	capsular polysaccharide biosynthesis protein Cap8F
12600	-2.97	2.86E-08	*fosB*	metallothiol transferase FosB
10865	-2.96	3.79E-07	-	ferrichrome-binding protein FhuD
12085	-2.96	7.43E-10	*rpsN*	30S ribosomal protein S14 type Z
4325	-2.96	1.03E-09	*seq*	enterotoxin
11295	-2.96	1.04E-08	-	transglycosylase SceD
1525	-2.96	8.95E-09	-	virulence factor EsxB
3135	-2.95	1.27E-07	-	hypothetical protein
825	-2.95	2.69E-08	*cap5E*	UDP-glucose 4-epimerase
13910	-2.93	2.62E-08	-	N-acetyltransferase
4330	-2.93	1.25E-09	-	hypothetical protein
14170	-2.93	4.34E-09	*nrdG*	anaerobic ribonucleoside-triphosphate reductase activating protein
13905	-2.92	1.54E-10	-	O-acetyltransferase OatA
10535	-2.92	2.11E-09	-	peptidoglycan hydrolase
1395	-2.92	8.12E-08	-	SAM-dependent methyltransferase
2870	-2.92	1.43E-09	-	ribulokinase
3020	-2.91	4.30E-09	-	hypothetical protein
14015	-2.90	1.86E-09	*pyrD*	dihydroorotate dehydrogenase (quinone)
13350	-2.89	9.83E-10	*opp-1A*	nickel ABC transporter, nickel/metallophore periplasmic binding protein
845	-2.89	1.04E-08	*cap5I*	capsular polysaccharide biosynthesis protein
4485	-2.88	2.42E-07	-	hypothetical protein
2895	-2.87	1.41E-08	-	deoxynucleoside kinase
5205	-2.87	7.05E-06	*purC*	phosphoribosylaminoimidazolesuccinocarboxamide synthase
3395	-2.87	1.87E-09	*fhuA*	ABC transporter ATP-binding protein
12875	-2.85	1.78E-08	-	hypothetical protein
5470	-2.84	7.21E-09	-	membrane protein
8960	-2.83	2.01E-09	-	alanine glycine permease
815	-2.83	1.52E-08	*cap5C*	capsular polysaccharide biosynthesis protein Cap8C
5215	-2.83	1.26E-07	*purQ*	phosphoribosylformylglycinamidine synthase subunit PurQ
4855	-2.81	8.36E-09	*pepF*	oligoendopeptidase F
1070	-2.81	5.69E-09	-	peptide ABC transporter substrate-binding protein

2175	-2.80	7.37E-09	*hsdM*	type I restriction-modification system subunit M
9310	-2.80	1.55E-08	-	MFS transporter
8565	-2.79	5.77E-09	-	O-methyltransferase
13185	-2.79	7.52E-10	-	sodium:proton antiporter
10290	-2.79	3.33E-10	*gatB*	Asp-tRNA(Asn)/Glu-tRNA(Gln) amidotransferase GatCAB subunit B
10910	-2.79	5.96E-10	-	CPBP family intramembrane metalloprotease
8515	-2.78	5.10E-09	-	divalent metal cation transporter
1765	-2.78	3.09E-07	-	PTS ascorbate transporter subunit IIA
6675	-2.78	1.54E-10	-	gamma-aminobutyrate permease
12080	-2.77	8.74E-10	*rpsH*	30S ribosomal protein S8
11545	-2.77	3.16E-10	*manA*	mannose-6-phosphate isomerase
11405	-2.77	1.95E-08	-	protein-(glutamine-N5) methyltransferase, release factor-specific
6535	-2.76	2.25E-08	-	hypothetical protein
14700	-2.76	5.35E-09	*rpmH*	50S ribosomal protein L34
3400	-2.75	1.23E-08	*fhuB*	iron ABC transporter permease
9615	-2.75	5.96E-09	*splB*	serine protease SplB
875	-2.75	2.45E-10	*cap5O*	nucleotide sugar dehydrogenase
8675	-2.74	1.21E-09	*recJ*	single-stranded-DNA-specific exonuclease RecJ
8650	-2.71	2.98E-08	*hisS*	histidine--tRNA ligase
2330	-2.70	3.59E-07	-	methionine import ATP-binding protein MetN 1
12445	-2.70	1.48E-06	-	hypothetical protein
11345	-2.69	9.15E-09	*atpA*	ATP synthase subunit alpha
1610	-2.67	1.40E-07	-	TIGR01741 family protein
11340	-2.66	1.81E-09	*atpG*	ATP synthase subunit gamma
11265	-2.66	5.68E-09	*oxaA*	protein translocase component YidC
2365	-2.65	2.79E-08	-	hypothetical protein
12970	-2.65	1.15E-06	-	hypothetical protein
810	-2.63	2.63E-08	*cap5B*	capsular polysaccharide biosynthesis protein Cap5B
10815	-2.63	1.44E-09	-	ATP-dependent helicase
985	-2.62	3.35E-10	*brnQ*	branched-chain amino acid transporter II carrier protein
11005	-2.60	8.69E-09	-	DNA mismatch repair protein MutS
1760	-2.60	3.04E-08	-	PTS lactose transporter subunit IIB
880	-2.59	2.56E-10	-	UDP-N-acetylglucosamine 2-epimerase (non-hydrolyzing)
1755	-2.58	6.15E-09	-	PTS ascorbate transporter subunit IIC
9440	-2.58	4.32E-08	-	camphor resistance protein CrcB
10215	-2.58	2.87E-09	-	membrane protein
6635	-2.57	1.64E-09	-	hypothetical protein
13265	-2.57	2.61E-08	-	amino acid:proton symporter
5385	-2.55	3.07E-08	*potB*	spermidine/putrescine ABC transporter permease
11410	-2.54	3.64E-09	*prfA*	peptide chain release factor 1
11750	-2.53	1.11E-08	-	iron-dicitrate ABC transporter permease
3025	-2.52	1.43E-08	-	DUF423 domain-containing protein
5390	-2.52	1.31E-08	*potC*	spermidine/putrescine ABC transporter permease

12690	-2.52	3.49E-08	-	hypothetical protein
12865	-2.52	2.46E-09	-	DUF4889 domain-containing protein
9135	-2.52	4.32E-09	-	1-acyl-sn-glycerol-3-phosphate acyltransferase
11350	-2.51	2.07E-09	*atpH*	ATP synthase subunit delta
1485	-2.51	1.42E-08	-	CHAP domain-containing protein
10375	-2.51	5.66E-09	*pheA*	prephenate dehydratase
4205	-2.51	9.71E-09	-	hypothetical protein
4145	-2.51	1.25E-09	-	DUF5067 domain-containing protein
11540	-2.50	2.95E-08	-	hypothetical protein
11355	-2.50	1.04E-08	*atpF*	ATP synthase subunit B
11415	-2.50	1.45E-09	*tdk*	thymidine kinase
12075	-2.49	4.66E-09	*rplF*	50S ribosomal protein L6
5890	-2.49	7.52E-10	*ileS*	isoleucine--tRNA ligase
5465	-2.49	5.10E-09	*cyoE*	protoheme IX farnesyltransferase
5045	-2.48	1.56E-09	-	glycosyl transferase family 1
3015	-2.48	2.81E-09	*ung*	uracil-DNA glycosylase
9605	-2.48	1.78E-08	*splD*	serine protease SplD
3490	-2.48	5.72E-07	-	peptidase M23
10340	-2.48	2.33E-09	-	staphopain A
11395	-2.48	5.96E-09	-	protein-tyrosine-phosphatase
10445	-2.47	5.93E-09	-	membrane protein
10305	-2.47	5.69E-09	*putP*	sodium:proline symporter
3205	-2.46	1.47E-09	*argS*	arginine--tRNA ligase
1590	-2.45	8.69E-08	-	TIGR01741 family protein
2475	-2.45	5.59E-10	*holB*	DNA polymerase III subunit delta'
2615	-2.45	6.96E-09	-	tRNA(Ile)-lysidine synthetase
5115	-2.45	6.73E-08	-	N-acetyl-L,L-diaminopimelate aminotransferase
5595	-2.45	2.11E-08	*rnhC*	ribonuclease HIII
3380	-2.44	3.44E-10	-	nucleoside permease
9610	-2.44	2.99E-09	*splC*	serine protease SplC
1460	-2.43	5.28E-08	-	ABC transporter permease
1035	-2.43	5.87E-10	*hsdR*	type-1 restriction enzyme R protein
3895	-2.43	1.21E-06	-	GrpB family protein
9595	-2.42	2.59E-08	*splF*	serine protease SplF
9130	-2.42	4.96E-10	*nagE*	PTS glucose transporter subunit IIBC
11335	-2.41	3.13E-09	*atpD*	ATP synthase subunit beta
14510	-2.41	6.69E-08	*hisC*	histidinol-phosphate aminotransferase
5220	-2.41	9.30E-08	*purL*	phosphoribosylformylglycinamidine synthase subunit PurL
6745	-2.41	8.83E-08	*sbcD*	exonuclease sbcCD subunit D
805	-2.40	4.89E-08	*cap5A*	capsular polysaccharide type 5 biosynthesis protein cap5A
11785	-2.40	1.71E-09	-	IucA/IucC family siderophore biosynthesis protein
2530	-2.38	3.09E-10	-	hypothetical protein
5230	-2.36	2.50E-07	*purM*	phosphoribosylformylglycinamidine cyclo-ligase
975	-2.36	2.14E-07	*argC*	N-acetyl-gamma-glutamyl-phosphate reductase

12640	-2.36	2.06E-09	-	sodium ABC transporter permease
5225	-2.33	1.42E-07	*purF*	amidophosphoribosyltransferase
13520	-2.33	1.03E-09	*galU*	UTP--glucose-1-phosphate uridylyltransferase
4795	-2.33	1.90E-06	*oppD*	ABC transporter ATP-binding protein
14270	-2.32	2.14E-09	*clfB*	clumping factor B
12070	-2.32	3.38E-09	*rplR*	50S ribosomal protein L18
8870	-2.30	2.21E-09	*rpmI*	50S ribosomal protein L35
1745	-2.29	5.72E-08	-	lipoate--protein ligase A
4140	-2.29	6.12E-09	-	hypothetical protein
13470	-2.29	8.80E-08	-	(deoxy)nucleoside triphosphate pyrophosphohydrolase
5920	-2.29	1.00E-05	*pyrP*	uracil permease
2700	-2.29	3.17E-09	-	pyridoxal 5'-phosphate synthase lyase subunit PdxS
1725	-2.29	8.33E-09	-	LLM class flavin-dependent oxidoreductase
6755	-2.27	4.62E-09	*mscL*	large-conductance mechanosensitive channel
390	-2.27	1.31E-07	-	NAD+ synthetase
8645	-2.26	4.72E-09	*aspS*	aspartate--tRNA ligase
6800	-2.26	2.22E-08	-	alanine:cation symporter family protein
7270	-2.25	2.40E-06	-	PepSY domain-containing protein
5450	-2.25	1.72E-08	-	cell division protein FtsW
3475	-2.25	4.62E-09	-	ABC transporter permease
11360	-2.25	2.90E-08	*atpE*	ATP synthase subunit C
515	-2.25	3.64E-08	*plc*	1-phosphatidylinositol phosphodiesterase
11425	-2.25	4.69E-09	*rho*	transcription termination factor Rho
5260	-2.25	2.98E-08	-	thiamine ABC transporter permease
5110	-2.23	4.88E-09	*sspA*	serine protease
4315	-2.23	2.38E-06	*int*	site-specific integrase
5560	-2.22	3.32E-06	-	hemin ABC transporter permease
12340	-2.22	3.80E-09	-	ferrichrome ABC transporter substrate-binding protein
9795	-2.20	8.98E-07	-	hypothetical protein
55	-2.20	1.95E-07	-	branched-chain amino acid ABC transporter permease
2480	-2.20	9.86E-10	-	signal peptidase II
1740	-2.19	8.13E-09	-	deacetylase SIR2
1475	-2.19	2.15E-06	-	DUF5079 domain-containing protein
2375	-2.19	1.62E-07	*gltC*	LysR family transcriptional regulator
1055	-2.18	8.50E-09	-	ABC transporter ATP-binding protein
9580	-2.18	8.69E-08	*hsdS*	specificity determinant HsdS
1735	-2.18	1.38E-07	-	hypothetical protein
11330	-2.17	1.08E-09	*atpC*	ATP synthase epsilon chain
8865	-2.17	1.10E-08	*rplT*	50S ribosomal protein L20
3340	-2.17	1.66E-09	*tagA*	N-acetylmannosaminyltransferase
12065	-2.16	5.65E-09	*rpsE*	30S ribosomal protein S5
5175	-2.16	3.16E-10	*qoxA*	quinol oxidase subunit 2
980	-2.16	1.72E-07	*rocD*	ornithine aminotransferase 1
8590	-2.16	1.17E-09	-	hypothetical protein

2620	-2.15	1.81E-09	-	hypoxanthine-guanine phosphoribosyltransferase
4175	-2.14	6.52E-07	*empbp*	hypothetical protein
7015	-2.14	1.68E-07	-	hydrolase
4335	-2.14	1.49E-08	-	transcriptional regulator
12965	-2.14	1.08E-07	-	formate/nitrite transporter
10335	-2.13	1.90E-08	*purB*	adenylosuccinate lyase
4785	-2.13	1.65E-08	*oppB*	ABC transporter permease
2805	-2.13	1.78E-08	*rplJ*	50S ribosomal protein L10
2705	-2.13	5.97E-09	-	pyridoxal 5'-phosphate synthase glutaminase subunit PdxT
610	-2.13	9.06E-07	-	siderophore biosynthesis protein SbnA
6900	-2.13	2.78E-08	-	hydrolase
5040	-2.12	2.86E-07	-	hypothetical protein
4790	-2.11	6.28E-07	*oppC*	ABC transporter permease
5770	-2.11	7.07E-08	-	hypothetical protein
5235	-2.11	2.60E-06	*purN*	phosphoribosylglycinamide formyltransferase
6385	-2.11	6.75E-09	-	metallophosphoesterase
14390	-2.11	3.04E-07	-	accessory Sec system protein translocase subunit SecY2
6245	-2.10	2.29E-09	*uppS*	isoprenyl transferase
4800	-2.10	3.52E-08	*oppF*	peptide ABC transporter ATP-binding protein
9530	-2.09	7.52E-07	-	hypothetical protein
1375	-2.09	1.58E-09	-	GntR family transcriptional regulator
9145	-2.09	3.19E-09	*tyrS*	tyrosine--tRNA ligase
11780	-2.09	7.43E-08	-	MFS transporter
2335	-2.08	4.35E-08	-	ABC transporter permease
12205	-2.07	3.23E-07	-	hypothetical protein
6340	-2.07	1.85E-09	-	peptidase M16
9515	-2.06	1.21E-09	-	DUF4352 domain-containing protein
2890	-2.06	2.49E-08	-	deoxynucleoside kinase
12060	-2.06	1.38E-08	*rpmD*	50S ribosomal protein L30
11365	-2.06	3.65E-09	*atpB*	ATP synthase subunit A
11480	-2.05	2.52E-08	-	DUF2750 domain-containing protein
7080	-2.05	7.89E-07	-	nitric oxide reductase activation protein NorD
5140	-2.05	4.28E-07	-	GNAT family N-acetyltransferase
12330	-2.03	1.68E-07	-	biotin transporter BioY
7085	-2.03	2.94E-07	-	hypothetical protein
10370	-2.02	1.94E-08	-	nitric oxide synthase oxygenase
2000	-2.01	1.08E-07	-	histidine phosphatase family protein
2905	-2.01	4.55E-08	-	HAD family phosphatase
13690	-2.01	5.21E-06	-	hypothetical protein
60	-2.01	2.04E-07	-	membrane protein
4680	-2.00	3.71E-08	*spsA*	inactive signal peptidase IA
4135	-2.00	5.37E-09	-	hypothetical protein
10820	-2.00	2.44E-07	-	hypothetical protein
6025	-2.00	5.96E-09	*pknB*	serine/threonine protein kinase

1075	-2.00	5.57E-09	*ggt*	gamma-glutamyltransferase
8420	-1.99	1.04E-08	*lepA*	elongation factor 4
12610	-1.99	3.23E-07	-	lysostaphin resistance protein A
7390	-1.99	1.18E-07	*aroB*	3-dehydroquinate synthase
14655	-1.98	1.14E-07	-	cold-shock protein
12565	-1.98	1.30E-08	-	sodium:proton antiporter
2485	-1.98	6.46E-09	-	initiation-control protein YabA
1135	-1.97	1.09E-07	*uhpT*	hexose phosphate transporter
6640	-1.97	1.29E-06	-	aspartate kinase
4805	-1.95	3.73E-08	*oppA*	peptide ABC transporter substrate-binding protein
12595	-1.94	1.31E-08	-	LysR family transcriptional regulator
8425	-1.94	1.97E-08	*rpsT*	30S ribosomal protein S20
12465	-1.94	2.85E-07	-	hypothetical protein
14340	-1.94	1.70E-09	-	phage infection protein
4950	-1.93	5.21E-08	*prfC*	peptide chain release factor 3
5170	-1.93	1.28E-09	*qoxB*	cytochrome ubiquinol oxidase subunit I
8480	-1.93	1.49E-08	-	ribosome biogenesis GTPase YqeH
3470	-1.93	3.34E-08	-	ABC transporter ATP-binding protein
4520	-1.92	5.87E-10	*dltB*	D-alanyl-lipoteichoic acid biosynthesis protein DltB
7285	-1.92	1.04E-07	-	RNA methyltransferase
3605	-1.92	1.47E-07	-	MarR family transcriptional regulator
6985	-1.91	4.19E-08	-	ABC transporter ATP-binding protein
7935	-1.91	8.18E-08	-	ribonuclease Z
9315	-1.90	2.55E-08	-	TIGR01212 family radical SAM protein
1480	-1.88	6.57E-07	-	DUF5080 domain-containing protein
9430	-1.88	1.49E-07	-	hypothetical protein
13345	-1.87	3.09E-08	-	peptide ABC transporter permease
1430	-1.86	1.39E-07	-	hypothetical protein
1145	-1.86	4.69E-08	-	sensor histidine kinase
365	-1.86	2.09E-07	-	methyltransferase
4530	-1.85	7.57E-10	*dltD*	D-alanyl-lipoteichoic acid biosynthesis protein DltD
970	-1.85	2.62E-07	*argJ*	bifunctional glutamate N-acetyltransferase/amino-acid N-acetyltransferase
9290	-1.84	8.89E-08	-	aminoacetone oxidase family FAD-binding enzyme
4770	-1.84	4.08E-09	*fabH*	3-oxoacyl-ACP synthase III
5975	-1.83	9.93E-08	*rpoZ*	DNA-directed RNA polymerase subunit omega
8670	-1.83	6.09E-08	*apt*	adenine phosphoribosyltransferase
4940	-1.83	9.85E-09	*murE*	UDP-N-acetylmuramoyl-L-alanyl-D-glutamate--L- lysine ligase
13770	-1.82	1.72E-06	-	CHAP domain-containing protein
13025	-1.82	1.95E-09	-	amino acid ABC transporter substrate-binding protein
5925	-1.81	8.21E-06	*pyrB*	aspartate carbamoyltransferase
4965	-1.81	2.00E-08	-	Ktr system potassium uptake protein D
4525	-1.81	3.91E-09	*dltC*	D-alanine--poly(phosphoribitol) ligase subunit 2
2985	-1.81	1.83E-06	-	long-chain-fatty-acid--CoA ligase

12325	-1.81	3.45E-08	-	N-acetyltransferase
13130	-1.80	1.28E-05	-	ABC transporter ATP-binding protein
8765	-1.80	2.25E-06	-	prepilin peptidase
6625	-1.80	5.25E-06	*nuc*	thermonuclease
4490	-1.80	3.21E-08	-	DUF86 domain-containing protein
2260	-1.79	8.98E-09	-	hypothetical protein
7150	-1.79	1.89E-09	*ctpA*	serine protease
10830	-1.79	7.44E-08	-	toxin MazF
9860	-1.78	1.08E-07	-	hypothetical protein
11460	-1.78	1.79E-08	*rpoE*	DNA-directed RNA polymerase subunit delta
3550	-1.77	2.15E-06	-	hypothetical protein
8815	-1.77	2.51E-08	*hemC*	hydroxymethylbilane synthase
2800	-1.76	3.47E-08	*rplA*	50S ribosomal protein L1
3150	-1.76	7.67E-07	-	DUF443 domain-containing protein
5835	-1.76	5.32E-09	*mraY*	phospho-N-acetylmuramoyl-pentapeptide- transferase
3095	-1.76	2.39E-09	-	transposase
7470	-1.75	2.87E-08	*ansA*	L-asparaginase
2340	-1.75	4.90E-08	-	ABC transporter substrate-binding protein
8375	-1.75	2.90E-09	-	tRNA (N(6)-L-threonylcarbamoyladenosine(37)-C(2))-methylthiotransferase MtaB
5720	-1.75	5.23E-08	-	alpha-hemolysin
4685	-1.75	3.68E-08	-	signal peptidase IB
9470	-1.74	5.07E-09	*pckA*	phosphoenolpyruvate carboxykinase (ATP)
3840	-1.74	9.96E-08	*nrdI*	protein NrdI
2640	-1.74	1.53E-07	*folP*	dihydropteroate synthase
1730	-1.74	3.00E-07	-	glycine cleavage system protein H
8475	-1.74	2.37E-08	*aroE*	shikimate dehydrogenase
11135	-1.74	1.95E-09	*rsbU*	serine phosphatase
3405	-1.73	1.73E-08	*fhuG*	iron ABC transporter permease
9740	-1.73	1.55E-06	-	serine hydrolase family protein
4660	-1.73	5.58E-07	*argH*	argininosuccinate lyase
4665	-1.73	4.73E-07	*argG*	argininosuccinate synthase
160	-1.73	1.09E-07	-	hypothetical protein
9840	-1.73	9.30E-08	-	DNA-binding response regulator
10430	-1.72	2.38E-07	-	choloylglycine hydrolase
10955	-1.72	1.93E-07	-	hypothetical protein
6820	-1.72	2.25E-08	*fmtC*	phosphatidylglycerol lysyltransferase
12200	-1.72	3.56E-06	-	hypothetical protein
6830	-1.72	8.50E-09	*msrR*	regulatory protein MsrR
2900	-1.71	6.19E-07	-	tRNA-specific adenosine deaminase
6020	-1.71	4.76E-08	-	protein phosphatase
13325	-1.70	2.98E-07	-	MFS transporter
10345	-1.70	1.57E-07	-	staphostatin A
9845	-1.69	7.17E-08	-	sensor histidine kinase
385	-1.69	2.02E-07	-	ABC transporter ATP-binding protein

3845	-1.69	1.08E-07	-	ribonucleotide-diphosphate reductase subunit alpha
10860	-1.69	2.81E-06	-	hypothetical protein
1250	-1.69	2.12E-07	-	L-lactate dehydrogenase
8285	-1.69	3.83E-09	-	DEAD/DEAH box family ATP-dependent RNA helicase
12185	-1.68	1.15E-07	*glcU*	glucose uptake protein GlcU
10810	-1.68	1.41E-07	-	XRE family transcriptional regulator
2345	-1.68	4.08E-05	-	N-acetylmuramoyl-L-alanine amidase
430	-1.68	1.37E-06	-	anion permease
5970	-1.67	1.32E-08	*gmk*	guanylate kinase
10420	-1.67	5.98E-06	-	YolD-like family protein
5240	-1.66	9.17E-07	*purH*	bifunctional phosphoribosylaminoimidazolecarboxamide formyltransferase/inosine monophosphate cyclohydrolase
8025	-1.66	8.36E-09	-	geranyltranstransferase
8595	-1.65	1.66E-07	-	hypothetical protein
8680	-1.65	2.69E-07	*secF*	protein translocase subunit SecDF
5940	-1.65	4.59E-08	*carB*	carbamoyl-phosphate synthase large chain
10385	-1.64	5.37E-09	-	sodium-dependent dicarboxylate transporter SdcS
5690	-1.64	1.02E-07	*efb*	fibrinogen-binding protein
1620	-1.64	7.97E-09	-	membrane protein
2510	-1.64	2.12E-09	*metS*	methionine--tRNA ligase
11250	-1.64	1.53E-07	-	phosphohydrolase
7035	-1.62	3.09E-09	-	cold-shock protein CspA
8280	-1.62	3.46E-08	-	endonuclease
12410	-1.62	9.26E-08	-	AraC family transcriptional regulator
1555	-1.62	2.76E-05	-	DUF5080 domain-containing protein
1150	-1.62	8.19E-08	-	lipoprotein
8490	-1.62	1.78E-07	*mtnN*	5'-methylthioadenosine/S-adenosylhomocysteine nucleosidase
1005	-1.61	3.71E-08	*ptsG*	PTS glucose EIICBA component
5695	-1.61	2.88E-07	-	fibrinogen-binding protein
2585	-1.60	3.74E-08	*mfd*	transcription-repair coupling factor
4675	-1.60	2.24E-07	-	TVP38/TMEM64 family protein
5930	-1.60	2.42E-07	*pyrC*	dihydroorotase
8485	-1.60	1.47E-07	-	hypothetical protein
13380	-1.60	3.06E-07	-	transporter
13160	-1.59	1.15E-07	-	Bcr/CflA family drug resistance efflux transporter
14385	-1.59	1.15E-05	-	accessory Sec system protein Asp1
10825	-1.58	2.76E-06	-	hypothetical protein
11180	-1.58	9.24E-08	*kdpC*	potassium-transporting ATPase subunit C
11185	-1.56	2.74E-07	*kdpB*	potassium-transporting ATPase subunit B
1095	-1.56	8.33E-07	-	sugar ABC transporter ATP-binding protein
6345	-1.56	4.55E-08	-	3-oxoacyl-ACP reductase
12855	-1.56	6.27E-09	-	transcriptional regulator
7280	-1.55	6.15E-07	-	DUF4889 domain-containing protein
4910	-1.55	2.73E-09	-	AI-2E family transporter

665	-1.55	1.07E-07	-	MFS transporter
1550	-1.55	4.66E-06	-	DUF5079 domain-containing protein
5165	-1.54	1.27E-08	*qoxC*	quinol oxidase subunit 3
7865	-1.54	2.51E-08	-	hypothetical protein
11190	-1.54	2.73E-07	*kdpA*	potassium-transporting ATPase A chain
510	-1.53	1.62E-07	-	hypothetical protein
11455	-1.53	3.00E-06	*pyrG*	CTP synthetase
6135	-1.53	2.89E-09	*rplS*	50S ribosomal protein L19
4835	-1.52	1.36E-08	*trpS*	tryptophan--tRNA ligase
12975	-1.52	2.40E-07	-	glutathione S-transferase
9115	-1.51	4.73E-07	*serA*	D-3-phosphoglycerate dehydrogenase
9665	-1.51	3.18E-07	*epiB*	hypothetical protein
11720	-1.51	8.47E-08	-	MFS transporter
2795	-1.50	1.25E-08	*rplK*	50S ribosomal protein L11
12230	-1.50	2.16E-05	-	MarR family transcriptional regulator
14535	-1.49	4.70E-07	-	N-acetyltransferase
2910	-1.49	7.54E-07	-	FMN-dependent NADPH-azoreductase
3000	-1.49	1.36E-05	-	hypothetical protein
7870	-1.49	3.18E-08	*srrB*	two-component sensor histidine kinase
8730	-1.49	3.88E-08	*rplU*	50S ribosomal protein L21
5005	-1.48	7.23E-06	-	hypothetical protein
890	-1.48	7.37E-06	-	DUF454 domain-containing protein
8655	-1.47	5.49E-07	*lytH*	cell wall amidase
12290	-1.47	4.27E-08	*moaC*	cyclic pyranopterin monophosphate synthase MoaC
7395	-1.47	1.82E-07	*aroC*	chorismate synthase
4280	-1.47	8.42E-08	-	hypothetical protein
5000	-1.46	8.06E-06	-	DUF2187 domain-containing protein
5105	-1.46	1.86E-07	*sspB*	staphopain B
5255	-1.46	1.61E-08	-	ABC transporter ATP-binding protein
1820	-1.45	1.45E-06	-	RimJ/RimL family protein N-acetyltransferase
12335	-1.45	8.53E-07	-	purine nucleosidase
13320	-1.45	3.69E-07	-	hypothetical protein
11450	-1.45	7.83E-08	-	hypothetical protein
2005	-1.45	1.03E-07	-	hypothetical protein
7370	-1.45	9.18E-07	-	membrane protein
6940	-1.44	1.49E-08	*pepF*	oligoendopeptidase F
90	-1.44	1.42E-03	*purA*	adenylosuccinate synthetase
11565	-1.44	6.77E-09	-	lytic regulatory protein
3640	-1.44	1.51E-05	-	hypothetical protein
2820	-1.43	9.67E-08	-	DNA-directed RNA polymerase subunit beta
8465	-1.43	5.08E-08	*nadD*	nicotinate-nicotinamide nucleotide adenylyltransferase
2645	-1.42	1.05E-07	*folB*	dihydroneopterin aldolase
2520	-1.42	1.31E-07	-	ribonuclease M5
10850	-1.42	3.93E-07	-	succinyl-diaminopimelate desuccinylase

5830	-1.42	8.39E-09	*pbpA*	penicillin-binding protein
505	-1.41	1.14E-08	-	ATPase
370	-1.41	1.16E-07	-	nickel ABC transporter substrate-binding protein
1850	-1.41	1.62E-05	-	hypothetical protein
3925	-1.40	2.13E-06	-	membrane protein
11195	-1.40	4.56E-05	-	potassium-transporting ATPase subunit F
380	-1.40	6.47E-06	*opp-3C*	ABC transporter permease
3830	-1.40	2.23E-07	*folE*	NADPH-dependent 7-cyano-7-deazaguanine reductase QueF
2325	-1.39	1.06E-06	*metB*	cystathionine gamma-synthase
13565	-1.39	6.12E-09	-	GTP pyrophosphokinase
755	-1.39	2.32E-05	*phnC*	phosphonates import ATP-binding protein PhnC
2810	-1.39	2.44E-06	*rplL*	50S ribosomal protein L7/L12
6235	-1.39	5.74E-08	*pyrH*	UMP kinase
12700	-1.38	9.93E-06	-	HlyD family secretion protein
14530	-1.37	4.77E-06	-	polysaccharide deacetylase
1605	-1.37	2.08E-05	-	TIGR01741 family protein
1455	-1.37	2.24E-06	-	ABC transporter ATP-binding protein
9270	-1.37	5.96E-09	-	dipeptidase PepV
11280	-1.37	2.33E-05	*thiD*	bifunctional hydroxymethylpyrimidine kinase/phosphomethylpyrimidine kinase
11285	-1.36	1.69E-05	-	thiaminase II
13830	-1.36	8.47E-08	-	TetR/AcrR family transcriptional regulator
11275	-1.36	1.73E-05	*thiM*	hydroxyethylthiazole kinase
9260	-1.35	1.08E-07	-	phosphotransferase
5245	-1.35	2.32E-06	*purD*	phosphoribosylamine--glycine ligase
14130	-1.35	5.38E-06	*betA*	oxygen-dependent choline dehydrogenase
3645	-1.35	2.15E-06	-	hypothetical protein
9265	-1.35	1.17E-08	*dat*	D-alanine aminotransferase
5585	-1.35	2.41E-07	*pheS*	phenylalanine--tRNA ligase subunit alpha
9900	-1.34	7.28E-07	-	PTS transporter subunit IIC
4565	-1.34	1.18E-08	*ampA*	cytosol aminopeptidase
12405	-1.34	7.83E-06	-	transcriptional regulator
2650	-1.33	3.15E-08	*folK*	2-amino-4-hydroxy-6- hydroxymethyldihydropteridine diphosphokinase
13060	-1.33	2.27E-07	*sbi*	immunoglobulin-binding protein sbi
1825	-1.33	3.37E-04	-	EfeM/EfeO family lipoprotein
6315	-1.33	2.41E-07	*pnpA*	polyribonucleotide nucleotidyltransferase
7385	-1.32	3.56E-08	*aroA*	3-phosphoshikimate 1-carboxyvinyltransferase
11245	-1.31	5.68E-07	-	cardiolipin synthase
2835	-1.31	1.00E-06	*rpsL*	30S ribosomal protein S12
12180	-1.31	1.12E-07	-	N-acetyltransferase
14320	-1.30	6.05E-06	-	hypothetical protein
800	-1.30	2.26E-06	*adhE*	bifunctional acetaldehyde-CoA/alcohol dehydrogenase
13605	-1.30	6.79E-06	-	lantibiotic ABC transporter ATP-binding protein
5430	-1.30	2.42E-07	*typA*	translational GTPase TypA

4735	-1.30	4.08E-06	-	LysR family transcriptional regulator
2885	-1.29	4.67E-07	-	HAD family hydrolase
10440	-1.29	4.33E-06	-	hypothetical protein
13125	-1.29	2.63E-05	-	ABC transporter ATP-binding protein
6125	-1.29	1.38E-05	*rimM*	ribosome maturation factor RimM
5825	-1.28	2.25E-08	*ftsL*	cell division protein FtsL
2830	-1.28	1.30E-06	-	50S ribosomal protein L7ae-like protein
6785	-1.28	2.46E-06	-	DNA topoisomerase IV subunit B
7525	-1.27	7.04E-05	-	hypothetical protein
7855	-1.26	2.39E-05	-	site-specific integrase
13485	-1.25	4.73E-07	-	hypothetical protein
4495	-1.25	5.99E-07	-	TIGR01457 family HAD-type hydrolase
2255	-1.25	2.17E-07	-	hypothetical protein
6130	-1.24	1.17E-05	*trmD*	tRNA (guanosine(37)-N1)-methyltransferase TrmD
2750	-1.24	5.30E-08	*gltX*	glutamate--tRNA ligase
1705	-1.23	2.50E-07	-	lipase
13435	-1.23	3.02E-05	-	hypothetical protein
6790	-1.23	2.23E-07	*parC*	DNA topoisomerase 4 subunit A
2590	-1.22	1.61E-07	-	stage V sporulation protein B
8030	-1.22	8.29E-07	*xseB*	exodeoxyribonuclease 7 small subunit
12880	-1.21	3.64E-08	-	transcriptional regulator
12235	-1.21	2.39E-05	-	MFS transporter
4755	-1.21	5.55E-05	-	phosphatidylethanolamine-binding protein
5120	-1.21	1.00E-05	-	acyltransferase
8810	-1.21	2.68E-06	*hemD*	uroporphyrinogen III methyltransferase
3750	-1.21	1.01E-06	-	para-aminobenzoate synthetase subunit I
12455	-1.20	2.32E-07	-	hypothetical protein
8995	-1.20	2.85E-08	-	NAD-dependent malic enzyme 4
12055	-1.20	5.44E-07	*rplO*	50S ribosomal protein L15
3655	-1.20	3.96E-07	-	hypothetical protein
9895	-1.20	1.61E-04	*(glnH)*	ABC transporter permease
1540	-1.20	4.87E-08	-	hypothetical protein
12540	-1.19	2.93E-06	-	sodium transporter
1385	-1.19	1.38E-06	*bglA*	6-phospho-beta-glucosidase
10635	-1.19	2.54E-05	-	phage major capsid protein
2970	-1.19	2.35E-05	-	HAD family hydrolase
2535	-1.19	3.53E-07	*ispE*	4-diphosphocytidyl-2C-methyl-D-erythritol kinase
5935	-1.18	3.82E-06	*carA*	carbamoyl-phosphate synthase small chain
9405	-1.18	2.62E-04	-	RNA polymerase sigma factor SigS
965	-1.18	1.74E-05	*argB*	acetylglutamate kinase
375	-1.17	3.63E-06	*opp-3B*	ABC transporter permease
9775	-1.17	6.42E-08	-	multidrug ABC transporter ATP-binding protein
12515	-1.16	5.16E-06	-	alanine glycine permease
760	-1.16	2.04E-04	-	phosphate/phosphite/phosphonate ABC transporter substrate-binding protein

5100	-1.16	1.37E-05	*sspC*	staphostatin B
2575	-1.16	1.67E-07	-	50S ribosomal protein L25/general stress protein Ctc
2655	-1.15	8.24E-07	-	hypothetical protein
9255	-1.15	8.47E-08	*trmB*	tRNA (guanosine(46)-N7)-methyltransferase TrmB
50	-1.15	1.49E-07	*serS*	serine--tRNA ligase
3835	-1.15	2.02E-06	-	EamA/RhaT family transporter
8725	-1.15	2.48E-07	-	hypothetical protein
11990	-1.15	5.49E-07	*rplM*	50S ribosomal protein L13
10115	-1.14	6.49E-06	-	membrane protein
9150	-1.14	2.50E-07	*sgtA*	transglycosylase
9535	-1.13	3.90E-05	-	hypothetical protein
6570	-1.13	7.63E-06	-	hypothetical protein
3590	-1.12	3.44E-07	-	undecaprenyl-diphosphatase
6430	-1.12	5.41E-06	*glpP*	glycerol-3-phosphate responsive antiterminator
1560	-1.11	2.08E-05	-	hypothetical protein
3745	-1.11	1.22E-05	*pabA*	aminodeoxychorismate/anthranilate synthase component II
2460	-1.11	2.01E-06	*tmk*	thymidylate kinase
6425	-1.11	6.98E-07	*mutL*	DNA mismatch repair protein MutL
8250	-1.10	3.95E-07	*rpmG*	50S ribosomal protein L33
13815	-1.10	4.39E-04	*feoB*	ferrous iron transporter B
2845	-1.10	4.85E-07	*fusA*	elongation factor G
11175	-1.09	1.25E-06	-	hypothetical protein
14325	-1.09	1.02E-06	-	PTS mannose transporter subunit IIA
10435	-1.09	5.52E-08	-	hypothetical protein
4620	-1.08	9.82E-06	-	kinase
3615	-1.08	1.24E-07	-	aldo/keto reductase
6750	-1.08	1.18E-07	*sbcC*	nuclease SbcCD subunit C
12435	-1.07	1.29E-06	-	membrane protein
13000	-1.07	2.63E-06	-	protein disulfide-isomerase
3755	-1.07	1.32E-05	-	aminodeoxychorismate lyase
6400	-1.07	7.37E-06	-	hypothetical protein
12830	-1.07	2.50E-06	-	phage infection protein
5590	-1.06	2.01E-07	*pheT*	phenylalanine--tRNA ligase subunit beta
2085	-1.06	1.26E-04	-	MazF/PemK family toxin
11170	-1.06	4.70E-07	-	hypothetical protein
7375	-1.06	5.33E-06	-	hypothetical protein
5160	-1.06	1.26E-07	*qoxD*	quinol oxidase subunit 4
5500	-1.05	1.40E-04	-	hypothetical protein
14025	-1.05	2.66E-06	-	hypothetical protein
8470	-1.04	2.77E-06	-	RNA-binding protein
2515	-1.04	1.45E-07	-	TatD family deoxyribonuclease
14155	-1.04	3.60E-05	*bccT*	choline transporter BetT
7315	-1.03	5.44E-07	*pbp2*	penicillin-binding protein 2
11160	-1.03	3.06E-06	*acpS*	holo-ACP synthase

1140	-1.03	1.58E-05	-	DNA-binding response regulator
1915	-1.03	1.25E-05	-	chromosome partitioning protein ParB
6005	-1.03	6.94E-07	*fmt*	methionyl-tRNA formyltransferase
500	-1.03	2.70E-05	-	hypothetical protein
3690	-1.02	6.12E-06	-	HlyC/CorC family transporter
3770	-1.02	1.78E-06	-	allophanate hydrolase
2490	-1.02	3.23E-04	-	tRNA1(Val) (adenine(37)-N6)-methyltransferase
14540	-1.02	1.23E-05	-	energy-coupling factor transporter transmembrane protein EcfT
13440	-1.01	7.41E-05	-	hypothetical protein
9380	-1.01	9.06E-05	-	transcriptional regulator
3210	-1.01	9.80E-04	-	endonuclease III domain-containing protein
7215	-1.01	2.69E-04	-	membrane protein

*Locus_tag: identifier corresponding to reference genome sequence, prefix to all numbers listed is "SAUSA300_RS", **logFC: $\log_2$ fold change, ***FDR: false discovery rate.

Supplementary Table 2e. VRE RBWH1 up-regulated genes following 1 h treatment with 1.75 μM PBT2 and 128 μM zinc in CAMHB grown at 37°C.

Locus_tag*	LogFC**	FDR***	Gene	Putative gene product function
1891	7.20	3.48E-10	*copZ3*	copper chaperone CopZ,Activator of copYZAB
1892	7.07	3.48E-10	*copY*	CopY/TcrY family copper transport repressor
1785	6.89	6.08E-11	*budB*	Acetolactate synthase
1269	6.74	1.02E-11	*zosA*	metal transporter
1896	6.50	8.19E-12	*cadA3*	zinc/cadmium/mercury/lead-transporting ATPase
1890	6.19	2.67E-11	*copA*	copper transporter
1784	5.74	2.67E-11	*aldC*	Alpha-acetolactate decarboxylase
987	5.51	4.58E-11	*ziaA*	cadmium ATPase
49	5.14	1.15E-07	*tcpE*	conjugative transposon membrane protein,TcpE family
725	4.84	9.93E-10	*glnR*	MerR family transcriptional regulator,HTH-type transcriptional regulator glnR,zinc-responsive transcriptional regulator
354	4.79	5.49E-11	-	zinc-containing alcohol dehydrogenase
1889	4.55	3.10E-11	*copB*	copper-translocating P-type ATPase
1553	4.43	1.87E-04	-	LPXTG-motif protein cell wall anchor domain protein
616	4.42	4.72E-08	*vanX1*	D-alanyl-D-alanine dipeptidase
2100	4.23	7.27E-08	-	Sulfite exporter TauE/SafE
726	4.22	3.70E-10	*glnA*	glutamine synthetase, type I
615	4.20	1.06E-06	*vanB1*	Vancomycin B-type resistance protein VanB,D-alanine--D-lactate ligase

50	4.16	8.53E-09	-	ATP/GTP-binding protein,Type IV secretory pathway, VirB4 components,type-IV secretion system protein TraC,AAA-like domain
1521	4.12	1.28E-08	*gspA1*	glycosyl transferase family protein,General stress protein A
2747	4.05	6.67E-05	-	transcriptional regulator, LacI family,DNA-binding transcriptional repressor MalI,ABC-type sugar transport system
1520	4.05	9.81E-07	-	hypothetical protein
1268	4.04	5.55E-10	-	integral membrane protein,Protein of unknown function (DUF441)
2759	3.98	9.63E-10	*dps*	Non-specific DNA-binding protein Dps, iron-binding ferritin-like antioxidant protein/ferroxidase
1504	3.96	1.02E-05	*amt*	ammonium transporter family protein
1522	3.94	1.34E-10	*gspA2*	glycosyl transferase family protein,rhamnosyltransferase
1246	3.86	6.52E-08	*suhB*	Inositol-1-monophosphatase
289	3.84	5.55E-09	*mobB*	plasmid recombinase enzyme Pre,Plasmid recombination enzyme
614	3.82	4.84E-06	*vanH1*	oxidoreductase, aldo/keto reductase family protein
1642	3.76	2.52E-09	-	hypothetical protein
1169	3.75	5.16E-10	-	putative lipase/esterase,Arylesterase,esterase,Predicted esterase,Alpha/beta hydrolase family
1245	3.75	4.83E-07	-	UPF0223 family protein,hypothetical protein,hypothetical protein,Uncharacterised protein family (UPF0223)
1519	3.72	7.67E-08	*corA2*	Mg^{2+} cation transporter,Magnesium transport protein CorA,zinc transporter,magnesium and cobalt transport protein CorA
1641	3.63	1.08E-09	-	hypothetical protein
1168	3.58	1.13E-09	-	hypothetical protein
1713	3.57	3.90E-06	*phnW*	2-aminoethylphosphonate--pyruvate transaminase
216	3.53	1.27E-08	-	plasmid stabilization system protein,addiction module toxin, Txe/YoeB family,Plasmid encoded toxin Txe
2099	3.53	4.36E-08	-	GNAT family acetyltransferase,ribosomal-protein-alanine acetyltransferase
1575	3.50	1.08E-09	*clpB*	ATP-dependent Clp protease
2278	3.48	2.37E-09	*lmrA*	Multidrug resistance ABC transporter
215	3.47	4.68E-09	-	prevent-host-death protein Phd,prevent-host-death family protein,Phd_YefM
1524	3.46	9.75E-10	-	isochorismatase,Isochorismatase family
355	3.43	3.48E-10	*hchA*	DJ-1/PfpI family protein,Molecular chaperone Hsp31 and glyoxalase 3
1715	3.32	5.60E-07	*phnX2*	Phosphonoacetaldehyde hydrolase
1286	3.32	5.48E-10	-	hypothetical protein
1523	3.31	9.75E-10	*yidA3*	HAD superfamily hydrolase,Phosphatase YidA
1714	3.29	1.47E-03	*phnX1*	Phosphonoacetaldehyde hydrolase
1434	3.27	1.13E-09	-	hypothetical protein,Uncharacterized protein conserved in bacteria,conserved domain,Domain of unknown function (DUF2382)
611	3.26	2.51E-09	*vanYB*	D-alanyl-D-alanine carboxypeptidase
1048	3.26	1.94E-07	-	hypothetical protein
2279	3.25	1.16E-08	-	TetR family transcriptional regulator,Bacterial regulatory proteins, tetR family
2614	3.22	4.65E-08	-	hypothetical protein

1672	3.19	4.29E-09	*hrcA*	heat-inducible transcription repressor HrcA
513	3.19	3.03E-06	-	peptidyl-prolyl cis-trans isomerase
1518	3.18	3.27E-09	*yghA*	short chain dehydrogenase/reductase family oxidoreductase
507	3.16	2.33E-10	*ydbD*	manganese-containing catalase
689	3.12	1.08E-05	*ydiB*	ATPase,hypothetical protein
1282	3.12	2.70E-07	-	hypothetical protein
2277	3.11	5.50E-09	*yheH2*	Probable multidrug resistance ABC transporter ATP-binding/permease protein YheH
690	3.10	1.40E-05	-	GNAT family acetyltransferase,ribosomal-protein-alanine acetyltransferase
612	3.10	1.69E-09	*vanW*	vancomycin B-type resistance protein VanW
2973	3.10	4.81E-07	*yvqK*	Cob(I)alamin adenosyltransferase PduO
2011	3.00	6.02E-09	-	hypothetical protein
2153	3.00	1.08E-04	-	hypothetical protein
2405	3.00	7.75E-08	-	hypothetical protein
737	2.97	3.72E-05	-	inner integral membrane protein,Protein of unknown function (DUF2512)
1096	2.97	1.38E-09	-	conserved hypothetical protein TIGR01655
1802	2.96	2.51E-09	*xerC7*	site-specific tyrosine recombinase XerC-family
2247	2.96	1.60E-09	-	hypothetical protein
468	2.94	2.14E-06	*yetF*	membrane protein,hypothetical protein,Protein of unknown function (DUF421)
1280	2.94	2.37E-09	*uspA*	universal stress protein UspA
2762	2.94	6.24E-08	*msrA*	Peptide methionine sulfoxide reductase msrA,
1127	2.94	9.75E-10	-	hypothetical protein
542	2.94	2.16E-09	-	superoxide dismutase,Uncharacterized protein conserved in bacteria,Protein of unknown function (DUF1033)
1503	2.93	5.54E-05	*gadC3*	amino acid antiporter,Glutamate/gamma-aminobutyrate antiporter
2174	2.92	6.91E-10	*yfkN*	Ser/Thr protein phosphatase family protein
2974	2.91	8.48E-07	-	Additional lipoprotein component of predicted cobalamin ECF transporter
960	2.91	9.75E-10	*pimB*	glycosyltransferase,GDP-mannose-dependent alpha-(1-6)-phosphatidylinositol monomannoside mannosyltransferase
2164	2.90	1.38E-09	-	hypothetical protein
2972	2.90	2.42E-08	*nrdE*	Ribonucleotide reductase of class II
1121	2.90	1.37E-07	*recQ1*	RecQ familyATP-dependent DNA helicase
1668	2.89	1.15E-09	*artM2*	amino acid ABC transporter ATP-binding protein
508	2.88	8.53E-09	*yesO1*	ABC transporter substrate-binding protein
1670	2.88	5.14E-10	*dnaK*	dnak protein,Heat shock protein 70,molecular chaperone DnaK
1322	2.87	5.06E-08	-	Predicted membrane protein
753	2.87	3.32E-09	*dinB1*	DNA polymerase IV
1671	2.86	1.15E-09	*grpE*	co-chaperone GrpE,HSP-70 cofactor,heat shock protein GrpE,GrpE
649	2.83	4.91E-09	*ndk*	nucleoside diphosphate kinase
1118	2.82	1.21E-06	-	
212	2.80	1.15E-09	-	zinc-containing alcohol dehydrogenase
1184	2.80	2.36E-09	-	hypothetical protein

1281	2.79	4.83E-09	-	hypothetical protein
1669	2.77	1.53E-07	*dnaJ*	Chaperone protein DnaJ
2192	2.77	4.27E-05	-	CsbD-like protein,CsbD-like
1299	2.76	1.37E-07	*yybR*	transcriptional regulator,Uncharacterized HTH-type transcriptional regulator yybR
3114	2.75	1.88E-09	*erm*	rRNA adenine N-6-methyltransferase
2849	2.73	2.65E-08	-	MazF family toxin-antitoxin system,PemK-like protein
1129	2.72	1.68E-08	-	IDEAL domain-containing protein,hypothetical protein,UPF0302 domain
1323	2.72	6.02E-08	*rnz*	Ribonuclease Z
699	2.69	5.44E-09	-	membrane protein
4	2.67	1.97E-09	*recF*	recombination protein F, RecF,DNA replication and repair protein recF
1425	2.67	2.61E-03	*iap1*	peptidase C60,Sortase
2848	2.65	1.23E-08	-	DNA-damage-inducible protein,bifunctional antitoxin/transcriptional repressor RelB,addiction module antitoxin, RelB/DinJ family,RelB antitoxin
585	2.65	6.77E-06	-	cell wall surface anchor family protein,Bacterial protein of unknown function (DUF916)
2303	2.65	3.99E-07	*nadE*	NH(3)-dependent NAD(+) synthetase
2137	2.64	1.38E-08	*radC*	DNA repair protein RadC,hypothetical protein,hypothetical protein,DNA repair protein RadC,Protein of unknown function (DUF2466)
494	2.63	7.10E-07	*lpd*	pyridine nucleotide-disulfide oxidoreductase
543	2.63	1.69E-09	*topB1*	DNA topoisomerase TopB
514	2.61	3.38E-07	-	von Willebrand factor domain-containing protein,Predicted outer membrane protein,Cna protein B-type domain
1120	2.59	2.70E-07	-	hypothetical protein
1744	2.59	4.93E-06	-	DUF2188 family protein,Uncharacterized protein conserved in bacteria,Uncharacterized protein conserved in bacteria (DUF2188)
404	2.58	3.04E-08	-	Urea transporter
2307	2.56	1.80E-07	*ccpB*	LacI family transcriptional regulator,Catabolite control protein B,DNA-binding transcriptional repressor PurR,ABC-type sugar transport system
1325	2.56	1.28E-09	-	Uncharacterized integral membrane protein
193	2.55	2.09E-08	*ytfL*	hemolysin,hypothetical protein,hypothetical protein,Putative Mg2+ and Co2+ transporter CorB,gliding motility-associated protein GldE,Domain of unknown function DUF21
2419	2.55	1.60E-09	*ecsB*	Bacterial ABC transporter protein EcsB,Predicted ABC-type exoprotein transport system, permease component,Bacterial ABC transporter protein EcsB
403	2.54	1.44E-07	*emrB1*	major facilitator superfamily transporter,Multidrug resistance protein B
1567	2.54	8.20E-07	-	alpha crystallin family heat shock protein,T786P28D,Hsp20/alpha crystallin family
1324	2.53	8.93E-09	*fabG3*	short chain dehydrogenase/reductase family oxidoreductase
2507	2.53	2.94E-08	*tetR*	TetR family transcriptional regulator,Tetracycline repressor protein class H
2780	2.53	1.15E-09	-	Protein of unknown function (DUF664).,DinB superfamily
1128	2.52	6.59E-09	-	tetratricopeptide repeat protein,tetratricopeptide repeat protein,Flp pilus assembly protein TadD
1681	2.52	2.37E-09	*ydeA*	DJ-1/PfpI family protein

3	2.52	8.26E-09	*yaaA*	S4 domain protein, YaaA,Uncharacterized conserved protein,S4 domain protein YaaA
2699	2.51	6.66E-05	*iap1*	NlpC/P6 family lipoprotein,Probable endopeptidase p6 precursor
364	2.51	1.76E-08	*frmR*	regulator protein FrmR,Copper-sensitive operon repressor,regulator protein FrmR,Uncharacterised BCR, COG1937
539	2.51	6.54E-07	*ydhC*	multidrug resistance protein,Inner membrane transport protein ydhC,drug efflux system protein MdtG
1586	2.51	1.21E-06	*erpQ*	ErpQ protein
267	2.51	2.77E-05	-	small secreted protein,Predicted small secreted protein
399	2.50	5.43E-09	*paiA*	GNAT family acetyltransferase
320	2.49	1.15E-09	*sph*	myosin-cross-reactive antigen-like protein
1502	2.48	1.50E-04	*glsA*	glutaminase
1605	2.48	1.27E-08	-	hypothetical protein
1063	2.46	1.60E-09	*luxA*	alkanal monooxygenase (FMN-linked)
775	2.46	2.11E-08	-	uracil-DNA glycosylase, family 4
2700	2.46	1.93E-03	-	conjugative transposon protein,Conjugative transposon protein TcpC
2401	2.45	5.41E-08	*rarA*	crossover junction endodeoxyribonuclease
272	2.45	1.08E-09	*htrA*	Serine protease do-like htrA
363	2.44	2.97E-07	-	DsrE/DsrF-like family protein
5	2.43	9.99E-10	*gyrB*	DNA gyrase subunit B
584	2.42	2.04E-08	-	WxL domain surface protein
509	2.42	8.39E-09	*ugpA1*	ABC transporter ATP-binding protein
2305	2.41	4.36E-08	-	Domain of unknown function (DUF1827)
321	2.40	2.20E-08	-	hypothetical protein
3126	2.38	5.36E-08	*rep1*	replication initiation factor,Replication initiation factor
204	2.38	7.17E-09	*ubiB*	ubiquinone biosynthesis protein UbiB
2035	2.38	2.05E-09	*est*	carboxylesterase
1469	2.37	1.12E-08	-	lipoprotein,Peptidase propeptide and YPEB domain
1515	2.37	1.32E-08	*gls33*	general stress protein Gls33,Stress response regulator gls24 homolog,Protein of unknown function (DUF322)
398	2.36	1.90E-08	-	sortase family protein,Sortase (surface protein transpeptidase),sortase,Sortase family
2126	2.36	1.31E-08	*miaA*	tRNA dimethylallyltransferase
832	2.36	1.14E-06	-	hypothetical protein
495	2.36	2.24E-08	*yfnB1*	isochorismatase hydrolase
1565	2.35	7.25E-07	-	DUF378-containing protein,Domain of unknown function (DUF378)
1886	2.34	1.72E-08	-	ATPase family protein,Uncharacterized conserved protein
501	2.33	1.57E-07	-	AbrB family transcriptional regulator
445	2.33	7.12E-08	*spxA1*	transcriptional regulator Spx-family,Regulatory protein spx
51	2.33	2.59E-06	-	amino acid transporter,Type IV secretory pathway, VirB10 components
2425	2.33	9.19E-08	-	DNA double-strand break repair ATPase,Uncharacterized conserved protein
1516	2.33	9.82E-07	*gapB*	stress response protein,Small integral membrane protein (DUF2273)
840	2.33	1.65E-03	-	prophage pi1 protein 09,Uncharacterized protein conserved in bacteria,Domain of unknown function (DUF771)

2327	2.33	1.15E-08	*proW*	Substrate binding domain of ABC-type glycine betaine transport system
2420	2.31	2.25E-08	*ecsA5*	ABC transporter ATP-binding protein,ABC-type transporter ATP-binding protein EcsA
1285	2.30	7.77E-09	-	hypothetical protein
3049	2.30	2.53E-06	*tee6*	cell wall surface anchor family protein,T6 antigen,Predicted outer membrane protein,fimbrial isopeptide formation D2 domain,Cna protein B-type domain
2891	2.30	2.67E-08	-	transposase,transposase, IS605 OrfB family,Probable transposase
1887	2.30	5.05E-09	-	Protein of unknown function DUF58,Uncharacterized conserved protein
156	2.29	7.17E-08	*metG1*	methionine-tRNA ligase
218	2.29	3.07E-05	-	mannosyl-glycoprotein endo-beta-N-acetylglucosaminidase,Endo-beta-N-acetylglucosaminidase D
961	2.28	2.51E-09	*mfpsA*	glycosyl transferase family protein
68	2.27	6.81E-07	*pdp*	pyrimidine-nucleoside phosphorylase
583	2.27	1.73E-05	-	diacylglycerol kinase catalytic subunit,Putative lipid kinase SP_1045
1470	2.27	8.57E-09	*cfa*	cyclopropane-fatty-acyl-phospholipid synthase
957	2.27	4.26E-08	*clpE*	ATP-dependent Clp protease
2696	2.26	1.87E-04	-	conjugative transposon membrane protein,TcpE family
502	2.26	1.05E-08	*ndoA*	toxin-antitoxin system
1065	2.26	1.41E-06	-	alpha/beta fold family hydrolase
988	2.26	1.64E-07	-	4-oxalocrotonate tautomerase
586	2.26	4.65E-05	-	cell surface protein,Protein of unknown function C-terminal (DUF3324)
800	2.25	4.40E-09	*corA2*	MIT family metal ion transporter CorA,magnesium and cobalt transport protein CorA,CorA-like Mg2+ transporter protein
1667	2.25	5.83E-09	*tcyB*	amino acid ABC transporter amino acid-binding/permease
1514	2.25	5.04E-09	-	transglycosylase,Transglycosylase associated protein
1284	2.24	8.46E-09	-	hypothetical protein
530	2.23	9.75E-10	-	hypothetical protein
956	2.23	2.80E-09	-	DUF1827 domain protein,Domain of unknown function (DUF1827)
353	2.22	2.81E-06	-	MarR family transcriptional regulator,Uncharacterized protein conserved in archaea,homoprotocatechuate degradation operon regulator, HpaR,MarR family
2034	2.22	9.99E-10	*rnr*	ribonuclease R
2631	2.21	1.64E-04	-	transcriptional regulator effector binding subunit,Bacterial transcription activator, effector binding domain
57	2.19	3.72E-08	*dhfr2*	dihydrofolate reductase
1984	2.19	6.79E-07	-	general stress protein,Gas vesicle protein,YtxH-like protein
141	2.19	6.71E-05	-	hypothetical protein
3069	2.18	1.33E-09	-	hypothetical protein
2555	2.18	2.88E-08	-	conserved hypothetical protein TIGR01033,Probable transcriptional regulatory protein HI_0315,hypothetical protein,DNA-binding regulatory protein, YebC/PmpR family,Domain of unknown function DUF28
1517	2.18	4.97E-07	*gapA3*	stress response protein

2125	2.18	2.05E-09	*hflX*	GTP-binding protein HflX,GTP-binding protein HflX,GTPase HflX,Predicted GTPase,GTP-binding protein HflX,GTPase of unknown function
1732	2.17	6.47E-08	-	putative uroporphyrin-III c-methyltransferase,Uncharacterized conserved protein,Protein of unknown function, DUF488
1604	2.17	6.02E-09	*gpmA1*	phosphoglycerate mutase
157	2.17	5.67E-09	*fabG1*	aldose dehydrogenase
2378	2.17	1.84E-07	-	competence protein CoiA-like family protein,Competence protein,Competence protein CoiA-like family
680	2.15	1.51E-06	*pnuC*	nicotinamide mononucleotide transporter PnuC,Nicotinamide mononucleotide transporter,nicotinamide mononucleotide transporter PnuC,Nicotinamide mononucleotide transporter
1995	2.15	5.64E-08	-	YibE/F-like protein,YibE/F-like protein
2101	2.15	1.60E-09	-	DUF1292 family protein,hypothetical protein,hypothetical protein,Uncharacterized protein conserved in bacteria,Protein of unknown function (DUF1292)
1360	2.14	1.97E-06	*ypsA*	YpsA like protein,hypothetical protein,hypothetical protein,Uncharacterized protein conserved in bacteria,Protein of unknown function (DUF1273)
743	2.13	4.50E-09	-	Putative virion core protein
2127	2.12	2.53E-08	*ugpQ*	glycerophosphodiester phosphodiesterase family protein
1159	2.12	9.12E-08	*yccU*	CoA-binding protein,hypothetical protein,acetyl coenzyme A synthetase (ADP forming), alpha domain,CoA binding domain
2186	2.11	2.14E-06	-	DRTGG domain-containing protein,putative manganese-dependent inorganic pyrophosphatase,Predicted transcriptional regulator containing CBS domains,inosine-5'-monophosphate dehydrogenase,DRTGG domain
3070	2.11	1.30E-07	*nrnA1*	Relaxase/mobilization nuclease domain protein
2130	2.11	8.46E-09	-	GNAT family acetyltransferase,ribosomal-protein-alanine acetyltransferase,Acetyltransferase (GNAT) family
1047	2.10	2.48E-07	-	hypothetical protein
1247	2.10	7.92E-09	*tufA*	elongation factor EF1A
1267	2.09	8.53E-09	-	hypothetical protein
684	2.09	5.91E-09	-	GNAT family acetyltransferase,ribosomal-protein-alanine acetyltransferase,Acetyltransferase (GNAT) family
2328	2.08	4.91E-08	*proV2*	glycine betaine transporter ATP-binding subunit
270	2.08	1.27E-08	-	universal stress family protein,Putative universal stress protein SAV1710
531	2.08	9.75E-10	*prs2*	ribose-phosphate pyrophosphokinase
1665	2.07	2.27E-09	*uvrA*	excinuclease ABC subunit A,Excinuclease ABC subunit A,excinuclease ABC subunit A,ABC-type transport system involved in cytochrome bd biosynthesis, fused ATPase and permease components,excinuclease ABC subunit A,ABC transporter
2363	2.07	4.81E-04	-	hypothetical protein
2893	2.06	4.05E-05	*treB*	PTS system mannose/fructose/sorbose transporter subunit IIA
1140	2.06	3.24E-08	-	DegV family protein,DegV domain-containing protein SPy_1493/M5005_Spy1226,EDD domain protein
2426	2.06	8.93E-09	*yfkN*	Ser/Thr protein phosphatase family protein

648	2.05	5.90E-05	-	transcriptional regulator,transcriptional activator, Rgg/GadR/MutR family, C-terminal domain,Helix-turn-helix domain
245	2.05	2.11E-05	-	deoxynucleoside kinase,Deoxyguanosine kinase,thymidylate kinase,Deoxynucleoside kinase
1858	2.05	2.24E-08	-	hypothetical protein
1720	2.04	1.60E-09	*arcA*	arginine deiminase
2179	2.04	2.69E-07	-	hypothetical protein
176	2.04	5.52E-08	-	hypothetical protein
1201	2.04	5.74E-08	-	transposase,transposase, IS605 OrfB family,Probable transposase
3071	2.04	8.91E-07	*mobC*	Bacterial mobilization protein C,Bacterial mobilisation protein (MobC)
1908	2.03	3.41E-09	-	hypothetical protein
2193	2.03	3.45E-07	-	transposase,transposase, IS605 OrfB family,Probable transposase
3127	2.03	3.24E-08	*rep2*	replication initiation factor,Replication initiation factor
517	2.02	8.03E-04	*iap1*	peptidase C60,Sortase
10	2.02	7.14E-09	*nrnA1*	DHH family protein
3066	2.02	3.60E-07	-	transposase like protein,Transposase and inactivated derivatives,Transposase IS200 like
2983	2.02	2.34E-06	*hin2*	Site-specific recombinases, DNA invertase Pin homologs,DNA-invertase hin,multiple promoter invertase,Resolvase, N terminal domain
155	2.02	2.32E-08	-	M protein trans-acting positive regulator,Mga helix-turn-helix domain
3122	2.01	6.09E-07	-	transposase like protein,Transposase and inactivated derivatives,Transposase IS200 like
446	2.01	1.18E-07	*spxA2*	Spx/MgsR family transcriptional regulator
2692	2.01	4.83E-09	-	hypothetical protein
2037	2.01	3.04E-09	-	GNAT family acetyltransferase,ribosomal-protein-alanine acetyltransferase
1478	2.01	2.22E-07	-	peroxiredoxin, Ohr family protein,Organic hydroperoxide resistance protein-like,peroxiredoxin, Ohr subfamily,OsmC-like protein
693	2.00	1.03E-08	-	zinc-containing alcohol dehydrogenase,Zinc-type alcohol dehydrogenase-like protein SA1988
1190	2.00	5.30E-06	-	hypothetical protein
2167	2.00	9.52E-09	*prsA1*	peptidyl-prolyl isomerase
512	2.00	8.94E-08	*ybjG*	undecaprenyl-diphosphatase
2187	1.99	3.24E-07	-	metallo-beta-lactamase domain-containing protein,hypothetical protein,metal-dependent hydrolase,Beta-lactamase superfamily domain
3120	1.99	4.08E-07	-	transposase like protein,Transposase and inactivated derivatives,Transposase IS200 like
3112	1.99	3.81E-09	-	Bacterial epsilon antitoxin.,Antitoxin epsilon,Bacterial epsilon antitoxin
1200	1.99	2.97E-07	-	transposase like protein,Transposase and inactivated derivatives,Transposase IS200 like
3119	1.99	1.27E-07	-	transposase,transposase, IS605 OrfB family,Probable transposase
217	1.99	3.13E-05	-	mannosyl-glycoprotein endo-beta-N-acetylglucosaminidase,Endo-beta-N-acetylglucosaminidase D

2890	1.99	5.81E-07	-	transposase like protein,Transposase and inactivated derivatives,Transposase IS200 like
261	1.98	1.19E-08	*nnrD*	YjeF protein,ADP-dependent (S)-NAD(P)H-hydrate dehydratase
2986	1.98	6.29E-08	-	hypothetical protein
1334	1.98	5.24E-08	*cysK*	cysteine synthase A
223	1.98	8.22E-08	-	transposase like protein,Transposase and inactivated derivatives,Transposase IS200 like
224	1.97	3.86E-07	-	transposase,transposase, IS605 OrfB family,Probable transposase
3123	1.96	1.60E-07	-	transposase,transposase, IS605 OrfB family,Probable transposase
2578	1.96	1.79E-07	*xylF*	phospholipase
1888	1.95	1.46E-08	-	Transglutaminase-like superfamily,Uncharacterized protein conserved in bacteria,Transglutaminase-like superfamily
1442	1.95	2.33E-07	-	transposase,transposase, IS605 OrfB family,Probable transposase
2085	1.95	4.98E-08	*gsiA1*	ABC transporter ATP-binding protein
395	1.94	6.59E-09	*treB*	PTS system glucose-specific transporter subunit IIABC
938	1.94	5.75E-09	*ghrB*	4-phosphoerythronate dehydrogenase
604	1.94	3.02E-05	-	hypothetical protein
2842	1.93	2.89E-03	-	hypothetical protein
2194	1.93	4.98E-07	-	transposase like protein,Transposase and inactivated derivatives,Transposase IS200 like
683	1.93	4.45E-07	-	primosomal protein N
3067	1.93	2.80E-07	-	transposase,transposase, IS605 OrfB family,Probable transposase
1666	1.92	2.06E-09	*ltrA1*	excinuclease ABC subunit B
62	1.92	2.99E-06	-	Protein of unknown function (DUF3173).,Domain of unknown function (DUF3173)
2310	1.91	1.06E-07	*groL*	chaperonin,hypothetical protein,chaperonin GroEL,chaperonin GroL,TCP-1/cpn60 chaperonin family
2059	1.91	3.53E-08	*gloA*	lactoylglutathione lyase
605	1.91	4.08E-07	-	relaxase,Relaxase/Mobilisation nuclease domain
11	1.90	7.67E-08	*rplI*	50S ribosomal protein L9,BL17
1994	1.90	5.78E-06	-	YibE/F-like protein,YibE/F-like protein
2304	1.90	1.39E-05	-	Nicotinate phosphoribosyltransferase
63	1.90	1.51E-08	*xerC1*	site-specific tyrosine recombinase XerC-family
685	1.89	2.11E-07	-	hypothetical protein
2242	1.89	2.77E-06	*acm*	Collagen-binding protein,Collagen adhesin precursor,Predicted outer membrane protein,Collagen binding domain
661	1.88	2.27E-07	-	Uncharacterized protein conserved in bacteria,Domain of unknown function (DUF305)
486	1.88	2.00E-09	-	phosphoenolpyruvate carboxykinase
366	1.88	6.87E-08	*cdr1*	coenzyme A disulfide reductase
1731	1.87	4.95E-07	-	zinc-containing alcohol dehydrogenase
2987	1.87	3.55E-08	-	hypothetical protein
211	1.87	2.67E-07	*rpsN1*	30S ribosomal protein S14
3111	1.86	7.27E-08	-	Omega Transcriptional Repressor
510	1.85	8.31E-08	*ycjP1*	ABC transporter permease,Inner membrane

269	1.85	1.85E-08	*trxA1*	thioredoxin
1985	1.84	6.60E-06	-	extracellular protein
2619	1.83	4.04E-07	-	ABC transporter ATP-binding protein
2157	1.83	4.82E-08	-	hypothetical protein
2373	1.82	4.50E-09	*relA1*	GTP diphosphokinase
1659	1.82	2.04E-05	-	hypothetical protein
2183	1.82	2.06E-09	-	ATP-dependent Zn protease
2054	1.82	4.20E-05	*ywiB*	DUF1934-containing protein,Uncharacterized beta-barrel protein ywiB,Uncharacterized protein conserved in bacteria,Domain of unknown function (DUF1934)
1338	1.81	1.09E-07	-	hypothetical protein
402	1.80	7.65E-07	-	MerR family transcriptional regulator,Hg(II)-responsive transcriptional regulator,MerR family regulatory protein
869	1.80	2.12E-05	-	Major phage capsid protein,Predicted phage phi-C31 gp36 major capsid-like protein,phage major capsid protein, HK97 family,Phage capsid family
698	1.80	3.06E-08	-	GNAT family acetyltransferase,ribosomal-protein-alanine acetyltransferase,Acetyltransferase (GNAT) family
2697	1.79	6.20E-05	-	ATP/GTP-binding protein,Type IV secretory pathway, VirB4 components,type-IV secretion system protein TraC,AAA-like domain
1782	1.79	9.61E-09	-	hypothetical protein
3047	1.78	2.34E-04	-	cell wall surface anchor family protein
540	1.78	2.06E-04	-	multidrug resistance protein,drug efflux system protein MdtG,sugar efflux transporter,Major Facilitator Superfamily
516	1.77	1.77E-04	-	LPXTG-motif protein cell wall anchor domain protein,Predicted outer membrane protein,fimbrial isopeptide formation D2 domain,Cna protein B-type domain
742	1.77	7.77E-09	-	Primosomal protein N' (replication factor Y) - superfamily II helicase
2793	1.77	9.52E-09	*clpC*	negative regulator of genetic competence ClpC/MecB
1409	1.76	1.14E-08	-	3-demethylubiquinone-9 3-methyltransferase,hypothetical protein,3-demethylubiquinone-9 3-methyltransferase
595	1.76	6.36E-02	-	Type IV secretory pathway, VirB4 components
2046	1.76	9.85E-08	*gapA1*	glyceraldehyde-3-phosphate dehydrogenase
515	1.75	4.02E-05	-	LPXTG-motif protein cell wall anchor domain protein,Predicted outer membrane protein
2313	1.74	1.97E-08	*treB*	,PTS system oligo-beta-mannoside-specific EIIC component
606	1.74	7.33E-05	-	Bacterial mobilisation protein (MobC).,Bacterial mobilisation protein (MobC)
1719	1.74	4.24E-09	*arcB*	ornithine carbamoyltransferase
3053	1.74	3.54E-08	*nrnA1*	PemK family protein
136	1.73	1.29E-07	*yueI*	hypothetical protein,Uncharacterized conserved protein,Protein of unknown function (DUF1694)
2995	1.73	1.51E-05	-	IS1216 transposase
1764	1.72	2.45E-06	-	HI0933-like protein,hypothetical protein,tricarballylate dehydrogenase,Aspartate oxidase,flavoprotein,
2693	1.72	5.18E-06	*thiF*	thiamine biosynthesis protein ThiF
71	1.72	1.84E-05	*tmpC*	ABC superfamily ATP binding cassette transporter
2615	1.71	1.60E-04	-	major facilitator superfamily transporter,Arabinose efflux permease,multidrug resistance protein,Major Facilitator Superfamily
1206	1.71	3.71E-05	-	hypothetical protein

2033	1.71	8.33E-09	*smpB*	SsrA-binding protein,SsrA-binding protein,SsrA-binding protein,SsrA-binding protein,SmpB protein
2092	1.70	1.48E-07	-	WxL domain surface protein
187	1.70	1.07E-08	*nadR*	transcriptional regulator,hypothetical protein,Predicted small molecule binding protein (contains 3H domain)
3113	1.70	2.94E-08	*fruA1*	PTS system fructose-specific transporter subunit IIA
6	1.70	2.02E-09	*gyrB*	DNA gyrase subunit A
1721	1.69	1.44E-07	*ntcA*	cyclic nucleotide-binding domain-containing protein,Global nitrogen regulator
281	1.69	2.28E-03	-	Antirestriction protein,Antirestriction protein,Antirestriction protein (ArdA)
2115	1.68	8.87E-08	*yheS2*	ABC transporter ATP-binding protein,Uncharacterized ABC transporter ATP-binding protein YheS
191	1.68	1.84E-03	-	transglycosylase,Transglycosylase associated protein
2372	1.68	2.02E-08	*ppnK*	inorganic polyphosphate/ATP-NAD kinase
2577	1.68	8.80E-07	-	bacterial seryl-tRNA synthetase related protein,Uncharacterized protein conserved in bacteria
1950	1.68	4.94E-06	*ybeZ*	KH domain-containing protein,PhoH-like protein,hypothetical protein
52	1.67	4.54E-03	*pgdS*	NlpC/P6 family lipoprotein
69	1.67	1.20E-06	*deoC*	deoxyribose-phosphate aldolase
2045	1.67	2.00E-09	*pgk*	phosphoglycerate kinase
2892	1.66	3.24E-06	*licB1*	PTS system mannose/fructose/sorbose transporter subunit IIB
1455	1.66	1.75E-08	-	hypothetical protein
3059	1.66	2.26E-04	-	DNA-directed RNA polymerase beta subunit
2188	1.65	2.35E-07	*ybaK2*	proline--tRNA ligase
2634	1.65	4.38E-04	*lacF2*	D-xylulose kinase
425	1.65	4.20E-08	-	N-acetylmuramoyl-L-alanine amidase
434	1.65	1.86E-07	*yhhT1*	Protein of unknown function UPF0118,hypothetical protein,pheromone autoinducer 2 transporter
2043	1.64	3.55E-09	*eno*	enolase
1853	1.64	5.47E-05	-	LPXTG-motif protein cell wall anchor domain protein,Predicted outer membrane protein
1709	1.64	3.17E-05	*pyrH*	UMP kinase
414	1.64	9.74E-04	-	integral membrane protein,Protein of unknown function (DUF554)
1993	1.64	3.41E-09	-	Hypothetical protein
474	1.63	2.63E-07	*araC1*	AraC-like ligand binding domain protein,Arabinose operon regulatory protein
1202	1.63	5.18E-09	*yuaG*	SPFH domain / Band 7 family protein,Inner membrane protein yqiK
3048	1.63	5.98E-07	-	sortase family protein,Sortase (surface protein transpeptidase),sortase,Sortase family
3058	1.62	4.33E-08	*polC1*	DNA polymerase IV
1352	1.62	1.44E-07	-	transposase (21),Transposase and inactivated derivatives
2794	1.62	2.95E-08	*ctsR*	CtsR family transcriptional regulator,Class three stress gene repressor
3052	1.62	1.73E-07	*mazE*	PemI family protein,Antitoxin MazE
2252	1.61	2.07E-07	*rimN*	Sua5/YciO/YrdC/YwlC family protein,t(6)A37 threonylcarbamoyladenosine biosynthesis protein RimN
2077	1.61	1.32E-07	*ltrA1*	excinuclease ABC subunit C

2138	1.60	1.54E-08	*yidA1*	HAD-superfamily hydrolase
365	1.60	1.80E-04	*moeZ*	rhodanese-like domain-containing protein,Probable adenylyltransferase/sulfurtransferase MoeZ
2007	1.60	4.44E-08	-	ribosomal subunit interface protein,hypothetical protein
695	1.60	6.30E-04	-	M protein trans-acting positive regulator,Mga helix-turn-helix domain
2038	1.59	4.98E-07	-	Alpha/beta hydrolase family protein
260	1.58	3.67E-08	-	thioesterase,hypothetical protein,acyl-CoA thioesterase YbgC,acyl-CoA thioester hydrolase, YbgC/YbaW family,Thioesterase superfamily
2334	1.58	3.38E-09	*nrdE2*	ribonucleoside-diphosphate reductase subunit alpha
663	1.57	2.06E-08	-	Eps11J
1170	1.57	2.13E-07	*galS*	LacI family transcriptional regulator,Mgl repressor and galactose ultrainduction factor
382	1.57	4.71E-07	*ecsA1*	ABC transporter ATP-binding protein,ABC-type transporter ATP-binding protein EcsA
1454	1.57	7.43E-09	-	hypothetical protein
2280	1.57	5.00E-08	-	rhodanese-like domain-containing protein,thiosulfate sulfurtransferase,Uncharacterized conserved protein
2931	1.57	2.57E-07	-	IS431mec transposase,Integrase core domain
2623	1.57	2.73E-08	*ydhF*	short chain dehydrogenase/reductase family oxidoreductase
3099	1.56	2.89E-07	-	toxin-antitoxin system, antitoxin component
686	1.56	7.07E-08	*yidA1*	HAD superfamily hydrolase
949	1.56	6.97E-08	*mutY*	A/G-specific adenine glycosylase
2055	1.56	4.18E-07	*lipL*	lipoate-protein ligase A
2185	1.56	3.20E-07	*nrnA1*	DHHA1 domain protein
511	1.55	8.22E-08	-	integral membrane protein,Predicted integral membrane protein,Protein of unknown function, DUF624
2778	1.54	4.04E-06	*gsiA1*	ABC transporter ATP-binding protein
2698	1.54	1.46E-05	-	membrane protein
675	1.54	1.72E-04	*sugE*	SMR family multidrug resistance protein,Quaternary ammonium compound-resistance protein sugE,multidrug efflux system protein
496	1.53	2.78E-08	*yxaF*	TetR family transcriptional regulator,Uncharacterized HTH-type transcriptional regulator yxaF
2933	1.53	1.05E-06	*xerD3*	phage integrase,Tyrosine recombinase XerD,site-specific tyrosine recombinase XerD
1509	1.53	4.87E-06	-	hypothetical protein
1320	1.52	5.07E-08	*gapA1*	glyceraldehyde-3-phosphate dehydrogenase
1468	1.52	1.20E-07	*pyrDA*	dihydroorotate dehydrogenase 1A
225	1.51	6.53E-08	*gsiA1*	glycine betaine/carnitine/choline transporter ATP-binding protein
1603	1.51	3.67E-08	*gph*	phosphoglycolate phosphatase
70	1.51	6.54E-06	*cdd*	cytidine deaminase
3095	1.50	9.30E-08	-	Reverse transcriptase (RNA-dependent DNA polymerase)
3098	1.50	1.66E-07	*relE2*	addiction module toxin RelE
2165	1.49	4.12E-07	*yugI1*	general stress protein 13,General stress protein 13,hypothetical protein,ribosomal protein S1,S1 RNA binding domain
1340	1.49	2.88E-06	-	MarR family transcriptional regulator,MarR family
1176	1.49	6.59E-09	*gor1*	pyridine nucleotide-disulfide oxidoreductase

227	1.49	4.20E-08	-	calcium-translocating P-type ATPase,Calcium-transporting ATPase lmo0841,magnesium-transporting ATPase
2777	1.49	2.04E-06	*lsrC2*	amino acid or sugar ABC transport system, permease protein,Autoinducer 2 import system permease protein lsrC
666	1.48	1.09E-06	*yheS1*	ABC transporter ATP-binding protein,Uncharacterized ABC transporter ATP-binding protein YheS,ABC transporter ATP-binding protein,ABC-type transport system involved in cytochrome c biogenesis, ATPase component,ATP-binding cassette protein, ChvD family,ABC transporter
1204	1.48	4.18E-07	-	membrane spanning protein,Predicted membrane protein,DUF939 C-terminal domain
2197	1.48	1.08E-02	-	putative phosphosugar isomerase/binding protein,hypothetical protein
2579	1.48	6.21E-08	-	hypothetical protein
2683	1.48	1.06E-04	*hpaIIM*	C-5 cytosine-specific DNA methylase
2253	1.48	3.05E-07	-	methyltransferase,ubiquinone/menaquinone biosynthesis methyltransferase
2044	1.48	6.17E-09	*tpiA*	triosephosphate isomerase
1952	1.47	2.25E-05	*brnQ2*	branched-chain amino acid transport system II carrier protein
2173	1.47	3.32E-08	*yutD*	Hypothetical DUF1027 protein,hypothetical protein,Uncharacterized protein conserved in bacteria,Protein of unknown function (DUF1027)
2311	1.47	1.36E-07	*groS*	chaperonin GroS,hypothetical protein
1272	1.47	7.67E-08	*gloB*	hydroxyacylglutathione hydrolase
2232	1.47	2.71E-06	-	GNAT family acetyltransferase,ribosomal-protein-alanine acetyltransferase,Acetyltransferase (GNAT) family
2093	1.47	2.55E-07	-	Integrase core domain protein,Transposase and inactivated derivatives, IS30 family,Integrase core domain
2302	1.46	1.52E-06	-	ArsR family transcriptional regulator,Uncharacterized protein conserved in archaea,CRISPR locus-related DNA-binding protein,Helix-turn-helix domain
497	1.46	1.38E-07	-	DUF159 family protein,Uncharacterised ACR, COG2135
1186	1.46	6.79E-06	-	hypothetical protein
2686	1.46	2.97E-02	-	Cro/CI family transcriptional regulator,Predicted transcriptional regulator,Replication initiation factor
498	1.46	9.00E-08	-	DUF159 family protein,Uncharacterised ACR, COG2135
284	1.46	1.61E-04	-	amino acid transporter
2627	1.45	7.67E-08	*treB*	PTS system N-acetyl glucosamine specific transporter subunits IIABC
2321	1.45	4.45E-07	-	DegV family protein,Domain of unknown function (DUF1836)
2083	1.45	5.02E-04	*lytA1*	N-acetylmuramoyl-L-alanine amidase
2156	1.45	1.10E-04	*xylF*	alpha/beta hydrolase family protein
186	1.45	1.32E-07	*maa*	maltose O-acetyltransferase
1209	1.45	2.25E-08	-	dihydrodipicolinate synthase/N-acetylneuraminate lyase,keto-hydroxyglutarate-aldolase/keto-deoxy-phosphogluconate aldolase
158	1.45	6.15E-08	*queT1*	membrane protein,Queuosine precursor ECF transporter S component QueT
1174	1.44	2.74E-08	*opuCC2*	glycine betaine/carnitine/choline ABC transporter,Osmoprotectant-binding protein
262	1.44	9.15E-07	*pepV*	dipeptidase PepV
948	1.44	3.04E-07	*recX*	recombination regulator RecX

1708	1.44	2.00E-06	*frr*	ribosome recycling factor
2320	1.44	4.76E-07	-	EDD domain-containing protein, DegV family
2139	1.43	2.09E-08	*folC*	tetrahydrofolate synthase
1837	1.43	8.41E-07	*ltrA2*	group II intron reverse transcriptase maturase,Group II intron-encoded protein ltrA,Retron-type reverse transcriptase,Reverse transcriptase (RNA-dependent DNA polymerase)
48	1.42	1.07E-06	-	hypothetical protein
2936	1.41	1.15E-06	-	ISEnfa3, transposase,Transposase and inactivated derivatives,Transposase
2776	1.41	1.12E-06	-	ABC transporter ATP-binding protein
2172	1.41	1.21E-08	*yfnB1*	HAD superfamily hydrolase
400	1.41	1.12E-04	*padC*	Phenolic acid decarboxylase
2637	1.40	5.06E-07	-	zinc-containing alcohol dehydrogenase
2912	1.40	6.11E-07	*xerS*	tyrosine recombinase
454	1.40	8.87E-06	*cysM*	cysteine synthase A
2616	1.40	1.22E-02	-	multidrug-efflux transporter,drug resistance MFS transporter, drug:H+ antiporter-2 (14 Spanner) (DHA2) family,Major Facilitator Superfamily
2996	1.39	2.31E-07	-	Integrase core domain protein,Integrase core domain
870	1.39	4.43E-03	-	prophage pi2 protein 34
628	1.39	6.19E-06	*ytcD*	HxlR-like helix-turn-helix protein,Uncharacterized HTH-type transcriptional regulator ytcD,HxlR-like helix-turn-helix
1441	1.38	4.28E-07	-	Integrase core domain protein,Integrase core domain
799	1.38	2.45E-07	-	hypothetical protein
3041	1.38	7.37E-07	-	Integrase core domain protein
3008	1.37	5.39E-07	-	Integrase core domain protein
3110	1.37	4.50E-07	-	Integrase core domain protein
3079	1.37	1.18E-06	-	Integrase core domain protein
2301	1.37	5.62E-05	*xsa*	alpha-N-arabinofuranosidase
294	1.37	8.03E-07	-	Integrase core domain protein,Integrase core domain
1813	1.37	2.85E-03	*sacA*	glycosyl hydrolase family protein
1527	1.36	2.33E-06	-	glucose uptake protein
2997	1.36	3.31E-07	-	addiction module toxin, Txe/YoeB family,addiction module toxin, Txe/YoeB family,Plasmid encoded toxin Txe
3060	1.36	2.83E-05	-	hypothetical protein
347	1.36	3.90E-07	*pbuG*	xanthine/uracil permease family protein,Guanine/hypoxanthine permease pbuG,Permeases,Permease family
3050	1.36	7.61E-07	-	Integrase core domain protein,Integrase core domain
16	1.36	1.07E-05	-	membrane protein,acid-resistance membrane protein,Uncharacterized conserved protein
3064	1.36	6.03E-07	-	Integrase core domain protein,Integrase core domain
1817	1.36	3.86E-04	*treB*	PTS system mannose-specific transporter subunit IIC
361	1.36	1.64E-07	*mutS21*	DNA mismatch repair protein MutS2
2935	1.35	3.58E-05	-	transposase,IS66 Orf2 like protein
3093	1.35	5.88E-07	-	Integrase core domain protein,Integrase core domain
1403	1.35	6.53E-07	-	hypothetical protein

2289	1.35	1.48E-03	*hprA*	D-isomer specific 2-hydroxyacid dehydrogenase
465	1.35	6.59E-09	*npr2*	NADH peroxidase
591	1.34	1.42E-02	*traG*	TraG/TraD family protein,Conjugal transfer protein traG
1444	1.34	2.06E-06	-	Integrase core domain protein,Integrase core domain
291	1.34	1.16E-06	-	Integrase core domain protein,Integrase core domain
3118	1.34	1.74E-06	-	Integrase core domain protein,Integrase core domain
2181	1.34	1.10E-02	-	TetR/AcrR family transcriptional regulator
1491	1.33	8.56E-08	*ybaL*	CPA2 family monovalent cation:proton (H+) antiporter-2,Inner membrane protein ybaL,glutathione-regulated potassium-efflux system protein KefC
2047	1.33	2.54E-07	*cggR*	central glycolytic genes regulator,Central glycolytic genes regulator,transcriptional regulator LsrR
940	1.33	1.50E-06	*czcD*	cation diffusion facilitator family transporter,Cadmium, cobalt and zinc/H(+)-K(+) antiporter
247	1.33	7.11E-06	-	pentapeptide repeat-containing protein,Uncharacterized protein conserved in bacteria,Pentapeptide repeats (8 copies)
1427	1.33	1.22E-07	-	TetR/AcrR family transcriptional regulator
3045	1.32	1.38E-07	*fms20*	LPXTG family cell surface protein Fms20,fimbrial isopeptide formation D2 domain
2971	1.31	2.48E-07	-	Integrase core domain protein,Integrase core domain
2592	1.31	1.22E-07	*nrnA1*	DNA recombination protein RecA
2151	1.31	3.38E-04	*fruA1*	Fructosamine kinase
1815	1.31	1.24E-04	*treB*	PTS system mannose/fructose/sorbose transporter subunit IIB
185	1.31	2.13E-07	*cobB*	NAD-dependent deacetylase
2547	1.31	1.19E-08	*yugI2*	S1 RNA binding domain-containing protein,General stress protein 13
1139	1.31	4.45E-07	*yqfA*	hemolysin III family channel protein,hypothetical protein
2546	1.30	8.38E-07	*tilS*	tRNA(Ile)-lysidine synthase
1173	1.30	1.28E-03	-	hypothetical protein
2324	1.30	4.25E-04	*entF*	enterocin induction factor
1208	1.30	8.35E-08	*lytG*	N-acetylmuramoyl-L-alanine amidase
2308	1.30	1.01E-03	-	hypothetical protein
1271	1.30	7.02E-07	*gpmA1*	phosphoglycerate mutase family protein
2985	1.30	7.74E-07	-	Integrase core domain protein,Integrase core domain
951	1.30	5.57E-06	-	GNAT family acetyltransferase,ribosomal-protein-alanine acetyltransferase,Acetyltransferase (GNAT) family
1885	1.29	2.88E-06	-	hypothetical protein
2086	1.29	1.64E-06	-	efflux ABC transporter permease,FtsX-like permease family
522	1.29	2.43E-07	-	tellurite resistance protein,TelA-like protein SA1238,Toxic anion resistance protein (TelA)
1207	1.29	8.25E-07	-	serine-type D-Ala-D-Ala carboxypeptidase,D-alanyl-D-alanine carboxypeptidase,D-alanyl-D-alanine carboxypeptidase
2576	1.29	1.11E-06	*yicC*	YicC protein,hypothetical protein
1160	1.29	2.82E-06	*parE*	DNA topoisomerase (ATP-hydrolyzing) subunit ParE
1175	1.27	3.06E-08	*proV1*	ABC transporter ATP-binding protein,Glycine betaine/L-proline transport ATP-binding protein ProV,component
741	1.27	5.84E-08	-	Beta-propeller domains of methanol dehydrogenase type,Domain of unknown function (DUF477)
2342	1.27	3.17E-06	-	SNARE associated Golgi protein

2976	1.26	1.07E-06	-	Integrase core domain protein,Integrase core domain
493	1.26	5.38E-04	*ohrB*	peroxiredoxin, Ohr family protein,General stress protein
2921	1.26	9.23E-06	*bin31*	resolvase family site-specific recombinase,Putative transposon Tn552 DNA-invertase bin3
2661	1.26	3.70E-05	-	prevent-host-death family protein,prevent-host-death family protein
536	1.26	3.86E-08	*mnmA*	tRNA-specific 2-thiouridylase MnmA
1379	1.26	2.40E-04	*rnhA*	ribonuclease HI
1814	1.25	1.50E-03	*fruA1*	PTS system mannose-specific transporter subunit IIA
2283	1.25	4.66E-07	*treB*	PTS system glucitol/sorbitol transporter subunit IIA
1529	1.25	4.82E-07	-	Pyrimidine dimer DNA glycosylase,conserved hypothetical protein,Pyrimidine dimer DNA glycosylase
2548	1.25	3.32E-08	-	septum formation initiator,Septum formation initiator
2502	1.25	3.75E-05	*xerC9*	site-specific tyrosine recombinase XerC-family
2590	1.24	3.38E-07	*proC*	pyrroline-5-carboxylate reductase
2102	1.24	3.97E-06	*ndoA*	Holliday junction resolvase-like protein
1145	1.24	2.48E-07	*sipS*	signal peptidase I
1205	1.23	5.22E-07	*afr*	dehydrogenase
1172	1.23	6.98E-04	-	YitT family protein,Uncharacterized BCR, YitT family COG1284
523	1.23	6.18E-08	-	5-bromo-4-chloroindolyl phosphate hydrolysis protein
1292	1.23	5.34E-07	-	hypothetical protein
319	1.23	2.47E-08	*fdh*	S-(hydroxymethyl)glutathione dehydrogenase
2618	1.22	9.62E-06	-	putative integral membrane protein,Protein of unknown function (DUF2798)
2371	1.22	3.03E-07	*rluD2*	23S rRNA pseudouridylate synthase RluD
599	1.22	1.11E-01	*topB1*	DNA topoisomerase III
14	1.22	1.20E-05	-	DegV family protein
142	1.22	2.28E-02	*pheS1*	phenylalanyl-tRNA synthetase subunit alpha
2930	1.22	6.36E-07	-	Integrase core domain protein
2129	1.22	1.12E-05	*clpP1*	ATP-dependent Clp protease proteolytic subunit ClpP
3061	1.22	5.42E-05	*prgN1*	replication control protein PrgN
1572	1.22	4.39E-07	-	GNAT family acetyltransferase,ribosomal-protein-alanine acetyltransferase,Acetyltransferase (GNAT) family
2993	1.21	3.14E-06	-	Integrase core domain protein,Integrase core domain
318	1.21	2.05E-07	-	hypothetical protein
1771	1.21	7.65E-04	-	ABC superfamily ATP binding cassette transporter, ABC protein
846	1.21	7.51E-04	-	phage protein,Protein of unknown function (DUF1071)
985	1.20	1.05E-04	-	hypothetical protein
2103	1.20	7.36E-07	-	Hypothetical protein possible functionally linked with Alanyl-tRNA synthetase
1861	1.20	1.19E-06	*azr*	flavin reductase,NADPH azoreductase
323	1.20	3.53E-07	-	membrane protein,Protein of unknown function (DUF1622)
2184	1.20	6.09E-06	*phnA*	putative alkylphosphonate utilization operon protein PhnA
1816	1.20	3.24E-04	*treB*	PTS system mannose-specific transporter subunit IID
1746	1.20	9.89E-05	*cbh1*	choloylglycine hydrolase family protein
2394	1.19	1.47E-07	*cypC*	Cytochrome P45,Fatty-acid peroxygenase

308	1.19	1.61E-03	*treB*	PTS system lactose/cellobiose transporter subunit IIC
1038	1.19	8.83E-04	*glpR*	DeoR family transcriptional regulator,Glycerol-3-phosphate regulon repressor
2041	1.19	7.84E-08	*dapE*	succinyl-diaminopimelate desuccinylase
2370	1.18	1.34E-07	*mgtE*	magnesium transporter,Magnesium transporter mgtE,inosine 5'-monophosphate dehydrogenase
1133	1.18	5.00E-08	-	hypothetical protein
1751	1.18	1.02E-06	-	cupin superfamily protein,Cupin domain
2220	1.18	5.60E-07	-	hypothetical protein
2932	1.17	8.25E-07	-	hypothetical protein
2241	1.17	1.29E-04	*proX2*	YbaK/prolyl-tRNA synthetase-associated domain protein
535	1.17	1.27E-06	-	Predicted phosphoesterase or phosphohydrolase,Calcineurin-like phosphoesterase superfamily domain
1435	1.16	2.14E-06	*xerC5*	site-specific tyrosine recombinase XerC-family
2595	1.16	2.23E-08	-	transcriptional regulator,Uncharacterized protein conserved in bacteria,Helix-turn-helix domain
1052	1.16	2.79E-05	-	ISEnfa3, transposase,Transposase and inactivated derivatives
1131	1.16	3.77E-07	-	NUDIX family hydrolase,NADH pyrophosphatase,NTP pyrophosphohydrolases containing a Zn-finger, probably nucleic-acid-binding,Hydrolase of X-linked nucleoside diphosphate N terminal
2256	1.15	3.28E-05	-	GNAT family acetyltransferase,ribosomal-protein-alanine acetyltransferase
2335	1.15	7.63E-07	*nrdE2*	ribonucleotide-diphosphate reductase subunit beta
412	1.15	5.60E-07	*phoR1*	sensor histidine kinase,Alkaline phosphatase synthesis sensor protein phoR
587	1.15	1.12E-05	-	WxL domain surface protein
461	1.15	4.00E-06	*asd*	aspartate-semialdehyde dehydrogenase
1293	1.14	9.84E-08	-	hypothetical protein
384	1.14	2.13E-07	-	polysaccharide deacetylase
324	1.14	1.74E-03	-	M protein trans-acting positive regulator
2694	1.14	1.72E-04	-	conserved hypothetical protein
2030	1.13	7.74E-08	*atpC*	ATP synthase F subunit B
3080	1.13	7.98E-06	-	ISEnfa3, transposase,Transposase and inactivated derivatives,Transposase
2743	1.13	1.99E-05	-	ISEnfa3, transposase,Transposase and inactivated derivatives,Transposase
1658	1.13	1.13E-01	-	hypothetical protein
1959	1.13	3.44E-07	*czrA*	ArsR family transcriptional regulator,HTH-type transcriptional repressor CzrA
1371	1.12	2.22E-05	-	hypothetical protein
1657	1.12	7.48E-07	-	bifunctional disulfide isomerase/thiol-disulfide oxidase,Protein-disulfide isomerase
1812	1.12	5.29E-04	-	Glyoxalase-like domain protein,methylmalonyl-CoA epimerase,Glyoxalase-like domain
2920	1.12	8.67E-07	*rpiR2*	RpiR family phosphosugar-binding transcriptional regulator,Als operon repressor
1545	1.11	1.43E-05	*murR1*	phosphosugar isomerase transcriptional regulator,MurPQ operon repressor
1752	1.11	1.30E-05	-	carboxymuconolactone decarboxylase family protein
2597	1.11	6.99E-07	*iap1*	Peptidase M16 inactive domain protein

838	1.11	7.31E-04	-	toxin-antitoxin system, toxin component, Bro family,Uncharacterized phage-encoded protein,Phage antirepressor protein KilAC domain
2665	1.10	3.76E-04	*nrnA1*	relaxase/mobilization nuclease domain protein
499	1.10	2.07E-02	-	hypothetical protein
1877	1.10	6.12E-06	-	hypothetical protein
2894	1.09	9.13E-04	*treB*	PTS system mannose/fructose/sorbose transporter subunit IIC
3087	1.09	3.05E-07	*prgO*	Prg family repication protein PrgO
2626	1.09	2.66E-06	*sacT*	BglG family transcriptional antiterminator,Levansucrase and sucrase synthesis operon antiterminator
2988	1.09	4.02E-05	-	Domain of unknown function (DUF1906)
3115	1.09	5.34E-06	*aphA*	kanamycin kinase
457	1.09	2.92E-06	*dapF*	diaminopimelate epimerase
2084	1.09	7.61E-07	*icaR*	TetR family transcriptional regulator,Intercellular adhesion protein R
613	1.09	2.90E-05	-	ISEnfa3, transposase,Transposase and inactivated derivatives,Transposase
1917	1.08	1.94E-04	*acyP*	acylphosphatase
721	1.08	1.41E-06	*xerC3*	site-specific tyrosine recombinase XerC-family,Integrase,site-specific tyrosine recombinase XerC,Site-specific recombinase XerD,tyrosine recombinase XerC,Phage integrase family
2191	1.08	1.19E-03	*gsiA1*	ABC transporter ATP-binding protein
396	1.08	6.97E-06	-	endonuclease/exonuclease/phosphatase family protein,Uncharacterized protein conserved in bacteria
2596	1.08	1.74E-07	-	M16C subfamily protease,protease3,coenzyme PQQ biosynthesis protein PqqF,Insulinase (Peptidase family M16)
118	1.08	3.64E-06	*truA1*	tRNA pseudouridine synthase A
1171	1.08	4.30E-07	-	hypothetical protein
431	1.08	8.86E-06	*gsiA1*	ABC superfamily ATP binding cassette transporter
937	1.07	3.96E-07	-	HesB-like domain-containing protein,Iron-sulphur cluster biosynthesis
1694	1.07	1.80E-07	*xpkA*	Xylulose-5-phosphate phosphoketolase
1600	1.06	1.36E-04	-	ISEnfa3, transposase,Transposase and inactivated derivatives,Transposase
2237	1.06	8.34E-05	*fliY*	family 3 extracellular solute-binding protein,Sulfate starvation-induced protein 7
1982	1.06	2.43E-07	*ccpA3*	catabolite control protein A,Catabolite control protein,DNA-binding transcriptional regulator CytR,ABC-type sugar transport system
1311	1.05	3.01E-06	*ykuL*	CBS domain pair protein
450	1.05	9.30E-06	*bsaA*	glutathione peroxidase
2827	1.05	1.36E-06	-	Integrase core domain protein,Integrase core domain
17	1.05	1.05E-06	*pox5*	pyruvate oxidase
3089	1.05	1.63E-06	*repR*	plasmid replication protein RepR,Primase C terminal 1 (PriCT-1)
651	1.04	7.90E-07	*mgtA*	magnesium-translocating P-type ATPase
53	1.04	5.72E-03	-	Conjugative transposon protein,Conjugative transposon protein TcpC
1606	1.04	4.25E-06	-	transcriptional regulator
462	1.04	9.01E-06	*lysC*	aspartate kinase

2717	1.03	4.93E-02	-	Integrase,Transposase and inactivated derivatives, IS30 family
2098	1.03	2.79E-04	*mleP*	CCS family citrate:sodium (Na+) symporter,Citrate transporter,citrate carrier protein, CCS family,2-hydroxycarboxylate transporter family
655	1.03	7.91E-03	*cbiO*	ABC transporter ATP-binding protein
2575	1.03	1.80E-06	-	hypothetical protein
2599	1.03	3.44E-07	-	acetyltransferase,hypothetical protein,putative acyltransferase
1867	1.03	2.10E-06	*phoP*	response regulator receiver domain-containing protein
3125	1.03	2.47E-04	*bin34*	Resolvase protein,Putative transposon Tn552 DNA-invertase bin3,multiple promoter invertase,Resolvase, N terminal domain
1992	1.03	1.79E-07	-	PspC domain-containing protein,phage shock protein C,PspC domain
2282	1.03	1.76E-07	*yhhT3*	permease,hypothetical protein,pheromone autoinducer 2 transporter,sporulation integral membrane protein YtvI,Domain of unknown function DUF20
3000	1.02	1.81E-06	*repA*	replication initiator protein A,Replication initiator protein A (RepA) N-terminus
189	1.02	1.10E-06	*rpsO*	30S ribosomal protein S15,BS18,30S ribosomal protein S15,ribosomal protein S15,Ribosomal protein S15
458	1.02	2.56E-06	*dapA*	dihydrodipicolinate synthase
600	1.02	1.59E-01	-	Antirestriction protein (ArdA)
720	1.02	5.85E-04	-	Domain of unknown function (DUF3173)
1559	1.02	1.67E-04	-	ISEnfa3, transposase,Transposase and inactivated derivatives,Transposase
2213	1.02	4.96E-06	*mgsA*	methylglyoxal synthase
1766	1.02	2.53E-06	*aroD*	3-dehydroquinate dehydratase
1585	1.01	1.70E-06	*nrdR*	transcriptional repressor NrdR
879	1.01	6.04E-03	-	phage minor structural protein, region,Phage-related protein,phage minor structural protein, N-terminal region,Prophage endopeptidase tail
1876	1.00	9.56E-05	-	ISEnfa3, transposase,Transposase and inactivated derivatives,Transposase

*Locus_tag: identifier corresponding to reference genome sequence, prefix to all numbers listed is “RBWH1_”, **logFC: $\log_2$ fold change, ***FDR: false discovery rate.

Supplementary Table 2f. VRE RBWH1 down-regulated genes following 1 h treatment with 1.75 µM PBT2 and 128 µM zinc in CAMHB grown at 37°C.

Locus_tag*	LogFC**	FDR***	Gene	Putative gene product function
2731	-8.04	3.38E-07	-	hypothetical protein
2734	-7.46	9.92E-09	*mntB2*	cation ABC transporter permease,Manganese transport system membrane protein mntB,high-affinity zinc transporter membrane component

2732	-7.32	1.19E-08	-	iron-dependent repressor family protein,manganese transport transcriptional regulator
479	-6.94	2.26E-08	*fhuC*	ABC transporter ATP-binding protein,Iron(3+)-hydroxamate import ATP-binding protein FhuC,manganese/iron transporter
31	-6.69	1.98E-11	*lacG1*	6-phospho-beta-galactosidase
480	-6.68	1.34E-07	*mntB1*	metal ion ABC transporter permease,Manganese transport system membrane protein mntB,high-affinity zinc transporter membrane component
32	-6.68	1.34E-10	*lacF1*	PTS system lactose-diacetylchitobiose-beta-glucoside transporter subunit IIA
2733	-6.51	9.63E-10	*ssaB2*	adhesion lipoprotein,Saliva-binding protein,high-affinity zinc transporter periplasmic component,ABC-type Zn2+ transport system
2735	-6.48	2.00E-09	*hmuV*	ABC transporter ATP-binding protein,Hemin import ATP-binding protein HmuV,manganese/iron transporter
481	-6.34	4.16E-09	*ssaB1*	periplasmic solute binding protein,Saliva-binding protein,high-affinity zinc transporter periplasmic component
137	-6.31	2.06E-09	*fecD*	iron ABC transporter system permease,Iron(III) dicitrate transport system permease protein fecD
33	-6.24	1.13E-09	*lacE1*	PTS system, lactose-specific IIC component
138	-5.84	5.16E-10	*feuC1*	iron ABC transporter system permease,Iron-uptake system permease protein FeuC
1072	-5.74	2.07E-05	-	hypothetical protein
1475	-5.70	1.18E-05	*bbmA*	alpha amylase
1070	-5.66	3.35E-06	*gatB1*	PTS system galactitol-specific transporter subunit IIB,
1236	-5.58	4.53E-08	*chvE*	multiple sugar-binding periplasmic receptor ChvE family protein,Multiple sugar-binding periplasmic protein sbpA precursor,D-xylose transporter subunit XylF,ABC-type xylose transport system, periplasmic component,D-xylose ABC transporter, D-xylose-binding protein,Periplasmic binding proteins and sugar binding domain of LacI family
30	-5.43	2.09E-09	*lacR1*	DeoR family transcriptional regulator, lactose phosphotransferase system repressor,Lactose phosphotransferase system repressor,DNA-bindng transcriptional repressor SrlR,Phosphotransferase system, mannose/fructose/N-acetylgalactosamine-specific component IIB,Bacterial regulatory proteins, deoR family
1071	-5.40	7.53E-07	*gatC1*	PTS system galactitol-specific transporter subunit IIC
139	-5.26	9.75E-10	*yclP*	iron ABC transporter ATP-binding protein
1370	-5.22	1.55E-09	*steT1*	amino acid permease,Serine/threonine exchanger SteT,putative fructoselysine transporter
29	-5.08	9.75E-10	*yvdM1*	beta-phosphoglucomutase
1369	-4.96	1.28E-08	-	NAD-dependent nucleoside-diphosphate-sugar epimerase
1473	-4.88	7.00E-07	*malF*	ABC transporter permease,Maltose transport system permease protein malF
1237	-4.87	2.51E-07	*araG*	ABC transporter ATP-binding protein,Xylose import ATP-binding protein
387	-4.84	1.23E-09	*ddc2*	tyrosine decarboxylase
1368	-4.80	5.50E-09	-	Putative 8-amino-7-oxononanoate synthase/2-amino-3-ketobutyrate coenzyme A ligase
140	-4.76	5.48E-10	*fetB*	ferric (Fe+3) ABC superfamily ATP binding cassette transporter, binding protein
1513	-4.74	3.89E-06	-	hypothetical protein
1068	-4.69	5.34E-06	*fruB*	PTS system galactitol transporter subunit IIA

1476	-4.61	3.14E-07	-	integral membrane protein,Uncharacterized BCR, YitT family COG1284
2267	-4.59	1.45E-05	*mtlF1*	PTS system mannitol-specific transporter subunit IIA
1486	-4.57	2.92E-09	*ahpC*	alkyl hydroperoxide reductase subunit C
2729	-4.55	1.37E-07	*cbiQ*	Cobalt transport protein,ABC-type cobalt transport system, permease component CbiQ and related transporters,Cobalt transport protein
473	-4.53	3.36E-10	*melB*	sodium/galactoside symporter,Thiomethylgalactoside permease II,melibiose:sodium symporter,sugar (Glycoside-Pentoside-Hexuronide) transporter,Major Facilitator Superfamily
1073	-4.50	1.28E-07	-	Class II Aldolase and Adducin N-terminal domain protein,hypothetical protein,Ribulose-5-phosphate 4-epimerase and related epimerases and aldolases,methylthioribulose-1-phosphate dehydratase,Class II Aldolase and Adducin N-terminal domain
388	-4.44	2.38E-09	*gadC2*	amino acid permease,Extreme acid sensitivity protein,inner membrane transporter
1389	-4.42	4.00E-06	*pyrP*	uracil permease,Uracil transporter,uracil transporter,uracil-xanthine permease,Permease family
2266	-4.32	4.22E-08	*mtlD*	Mannitol-1-phosphate 5-dehydrogenase
538	-4.32	3.94E-09	*aspC*	aspartate aminotransferase
537	-4.31	4.22E-08	*arcD*	arginine/ornithine APC family amino acid-polyamine-organocation transporter, antiporter
1471	-4.24	2.16E-06	-	integral membrane protein,Predicted integral membrane protein
968	-4.23	8.17E-10	*fabD*	Malonyl CoA-acyl carrier protein transacylase
472	-4.23	3.90E-10	*galM*	aldose 1-epimerase
1716	-4.23	6.91E-10	*argS*	arginyl-tRNA synthetase
1872	-4.21	5.70E-08	-	hypothetical protein
471	-4.20	1.34E-10	*galK*	galactokinase
1404	-4.17	5.15E-07	-	ABC transporter substrate binding protein
2728	-4.17	1.07E-08	*ykoD*	ABC transporter ATP-binding protein,Putative HMP/thiamine import ATP-binding protein YkoD,cobalt transporter ATP-binding subunit,ABC-type Na+ transport system
969	-4.15	1.13E-09	*fabG2*	3-oxoacyl-ACP reductase,3-oxoacyl-[acyl-carrier-protein] reductase FabG
1472	-4.14	2.90E-07	*mdxG*	ABC transporter permease,Maltose transport system permease protein malG
1530	-4.09	1.79E-04	-	prevent-host-death family antitoxin
1390	-4.05	6.79E-07	*pyrB*	aspartate carbamoyltransferase
965	-4.04	5.46E-10	*marR1*	MarR family transcriptional regulator,Multiple antibiotic resistance protein marR
1407	-4.04	3.96E-07	*aroE*	shikimate 5-dehydrogenase
967	-4.03	6.91E-10	*acpA*	acyl carrier protein,Acyl carrier protein,acyl carrier protein,Acyl carrier protein,acyl carrier protein,Phosphopantetheine attachment site
3055	-3.99	7.02E-09	-	hypothetical protein
1487	-3.98	5.16E-10	*ahpF*	peroxiredoxin subunit F,NADH dehydrogenase
1826	-3.98	1.36E-05	-	MarR family transcriptional regulator,homoprotocatechuate degradation operon regulator, HpaR,MarR family
971	-3.97	9.99E-10	*accB*	acetyl-CoA carboxylase biotin carboxyl carrier subunit

389	-3.96	5.96E-09	*nhaC1*	Na+/H+ antiporter,Sodium/proton antiporter,Na+/H+ antiporter NhaC,Na+/H+ antiporter family
970	-3.96	2.37E-09	*fabF*	beta-ketoacyl-acyl-carrier-protein synthase II
1388	-3.95	2.13E-05	*pyrR*	pyrimidine operon attenuation protein
3056	-3.93	1.12E-07	-	hypothetical protein
2854	-3.91	2.65E-08	*ugpA6*	carbohydrate ABC superfamily ATP binding cassette transporter, membrane protein transport system, permease component,NifC-like ABC-type porter,Binding-protein-dependent transport system inner membrane component
966	-3.88	5.16E-10	*fabH*	3-oxoacyl-ACP synthase
972	-3.86	2.88E-09	*fabZ2*	(3R)-hydroxymyristoyl-[acyl carrier protein] dehydratase
1382	-3.85	1.45E-07	*fhs*	formate-tetrahydrofolate ligase
1424	-3.82	3.50E-06	*ybbH2*	RpiR family transcriptional regulator,Uncharacterized HTH-type transcriptional regulator ybbH
2475	-3.80	6.52E-08	*licB4*	PTS system cellobiose-specific transporter subunit IIB,Lichenan-specific phosphotransferase enzyme IIB component
1391	-3.80	5.65E-07	*pyrC*	Dihydroorotase,Dihydroorotase
930	-3.79	1.15E-07	*fmtA*	Beta-lactamase,hypothetical protein,beta-lactamase/D-alanine carboxypeptidase
1622	-3.76	5.71E-07	*aroF*	phospho-2-dehydro-3-deoxyheptonate aldolase
1555	-3.75	2.32E-07	*prsA2*	foldase protein PrsA
1315	-3.71	8.46E-09	*fabZ1*	(3R)-hydroxymyristoyl-[acyl carrier protein] dehydratase
973	-3.71	1.97E-09	*cfiB1*	acetyl-CoA carboxylase biotin carboxylase subunit
2856	-3.70	7.14E-09	*lipO2*	carbohydrate ABC superfamily ATP binding cassette transporter, binding protein,Lipoprotein lplA,Maltose-binding periplasmic proteins/domains
1483	-3.69	1.66E-07	*uxuA*	mannonate dehydratase
1303	-3.68	2.78E-08	*trpS*	tryptophan-tRNA ligase
2855	-3.67	1.27E-08	*ycjP9*	carbohydrate ABC superfamily ATP binding cassette transporter, membrane protein
1405	-3.66	5.99E-07	*lsrC1*	amino acid or sugar ABC transport system, permease protein
1741	-3.60	8.33E-09	*feuA*	iron (Fe3+) ABC superfamily ATP binding cassette transporter
2730	-3.59	3.50E-09	-	transcriptional regulator,Bacterial regulatory proteins, tetR family
892	-3.59	1.89E-08	*acrR*	TetR/AcrR family transcriptional regulato
2727	-3.58	1.85E-06	-	conserved hypothetical integral membrane protein
1623	-3.57	4.05E-09	*lacM*	Beta-galactosidase small subunit
1406	-3.57	5.77E-08	*cmpD*	ABC transporter ATP-binding protein,Bicarbonate transport ATP-binding protein CmpD
2857	-3.54	8.52E-08	-	Predicted integral membrane protein
2207	-3.53	3.62E-09	*luxO3*	sigma-54 dependent DNA-binding transcriptional regulator,Regulatory protein luxO,anaerobic nitric oxide reductase transcription regulator
974	-3.48	2.00E-09	*accD*	Acetyl-coenzyme A carboxylase carboxyl transferase subunit beta
2332	-3.48	5.79E-07	*feoA*	ferrous iron (Fe2+) uptake protein FeoA,ferrous iron transport protein A,FeoA domain
1411	-3.45	4.04E-05	-	hypothetical protein
1355	-3.44	2.55E-05	-	OsmC-like protein
2651	-3.44	1.23E-08	-	HAD superfamily hydrolase

1484	-3.41	1.10E-07	*por*	mannitol dehydrogenase protein
2268	-3.40	2.83E-08	*mtlA1*	PTS system mannitol-specific transporter subunit IICBA
2737	-3.39	2.38E-09	*yidK*	Sodium/myo-inositol cotransporter,Uncharacterized symporter yidK
1474	-3.38	1.94E-08	*malX*	maltose/maltodextrin ABC superfamily ATP binding cassette transporter, binding protein
975	-3.38	2.02E-09	*accA*	cetyl-coenzyme A carboxylase carboxyl transferase subunit alpha
1393	-3.38	1.32E-08	*carB*	carbamoyl-phosphate synthase large subunit
1392	-3.37	4.04E-07	*carA*	carbamoyl-phosphate synthase small subunit
1408	-3.36	1.70E-08	*msrR*	transcriptional regulator, PSR protein,Regulatory protein msrR
2133	-3.33	3.46E-09	-	ABC transporter ATP-binding protein/permease,Putative multidrug export ATP-binding/permease protein SAV1866
423	-3.24	2.02E-08	*galM2*	aldose 1-epimerase
1265	-3.23	1.97E-09	*rpsT*	30S ribosomal protein S20
2788	-3.22	1.57E-09	*apbE*	thiamine biosynthesis lipoprotein,Thiamine biosynthesis lipoprotein ApbE precursor,thiamine biosynthesis lipoprotein ApbE,ApbE family
2736	-3.21	7.13E-08	-	cellobiose phosphotransferase system IIB component
2469	-3.21	1.89E-08	-	integral membrane protein,Uncharacterized BCR, YitT family COG1284
2652	-3.21	4.49E-09	*manZ5*	PTS system mannose/fructose/sorbose-specific IID component
2726	-3.20	1.42E-08	*irtA*	ABC transporter ATP-binding protein,Iron import ATP-binding/permease protein IrtA
1497	-3.19	1.62E-08	-	flavin reductase,short chain dehydrogenase
1540	-3.17	2.85E-05	*agaC1*	PTS system mannose/fructose/sorbose-specific IIC component
2787	-3.14	5.96E-08	-	FMN-binding domain-containing protein,Major membrane immunogen, membrane-anchored lipoprotein
2858	-3.13	4.29E-09	-	family 20 glycoside hydrolase,N-acetyl-beta-hexosaminidase
2653	-3.12	6.85E-09	*manY2*	PTS system mannose/fructose/sorbose-specific IIC component
2214	-3.11	3.64E-09	*msmK*	ABC transporter ATP-binding protein,sn-glycerol-3-phosphate import ATP-binding protein UgpC
2446	-3.07	2.38E-09	*purR1*	LacI family transcriptional regulator,Purine nucleotide synthesis repressor
440	-3.06	1.77E-06	-	hypothetical protein
2377	-3.05	1.08E-09	*pepF*	M03 family oligopeptidase F
1270	-3.03	1.37E-08	*uppP*	undecaprenyl-diphosphatase,Undecaprenyl-diphosphatase
1801	-3.03	1.72E-08	-	transcriptional regulator,Uncharacterized protein conserved in bacteria (DUF2087)
2786	-3.02	9.63E-10	*yjlD*	pyridine nucleotide-disulfide family oxidoreductase
1260	-3.01	1.98E-06	-	esterase
292	-3.01	3.00E-03	-	hypothetical protein
1496	-3.01	6.69E-07	-	hypothetical protein
893	-3.01	8.41E-09	*mioC*	flavodoxin,hypothetical protein
1740	-2.99	1.68E-06	*feuC2*	iron (Fe^{3+}) ABC superfamily ATP binding cassette transporte
2739	-2.99	5.11E-08	*idhA2*	oxidoreductase family NAD-binding Rossmann fold protein
2222	-2.99	2.52E-09	*metG2*	methionyl-tRNA synthetase

252	-2.99	6.38E-05	-	CitMHS family citrate-magnesium (Mg2+):proton (H+) citrate-calcium (Ca2+): proton (H+) symporter
1611	-2.98	1.91E-04	*ycjO2*	ABC transporter permease
2458	-2.97	7.58E-08	-	DUF1237 family protein,Uncharacterized conserved protein
1283	-2.97	1.26E-02	-	glucose uptake protein
1163	-2.96	3.31E-09	*pflA*	pyruvate formate-lyase activating enzyme
2654	-2.96	4.45E-08	*levE1*	PTS system mannose/fructose/sorbose-specific IIB component
1500	-2.96	9.76E-08	*dxs*	1-deoxy-D-xylulose-5-phosphate synthase
2738	-2.96	4.13E-09	*iolE*	inosose dehydratase
1620	-2.96	4.97E-07	*aroC*	chorismate synthase
2820	-2.96	2.95E-08	-	hypothetical protein
760	-2.94	3.26E-08	*dltB*	GNAT family acetyltransferase,ribosomal-protein-alanine acetyltransferase
184	-2.94	2.65E-08	-	hypothetical protein
1439	-2.90	2.09E-08	-	Hypothetical protein ywlG,hypothetical protein,hypothetical protein,Uncharacterized protein conserved in bacteria,conserved hypothetical protein,Protein of unknown function (DUF436)
2482	-2.90	8.46E-09	*msbA*	ABC transporter ATP-binding protein,Lipid A export ATP-binding/permease protein MsbA,cysteine/glutathione ABC transporter membrane/ATP-binding component
734	-2.90	1.16E-06	*fruA2*	PTS system fructose-specific transporter subunit IIBC
1934	-2.90	2.65E-06	*wcaJ2*	glycosyl transferase family protein,rhamnosyltransferase
86	-2.89	2.02E-08	*rplC*	LSU ribosomal protein L3P,50S ribosomal protein L3
762	-2.87	1.51E-08	*dltD*	D-alanyl-lipoteichoic acid synthetase
1357	-2.86	1.06E-04	-	transposase
761	-2.86	7.68E-07	*dltC*	D-alanine-poly(phosphoribitol) ligase subunit 2
1619	-2.86	1.56E-07	*tyrC*	prephenate dehydrogenase,Arogenate dehydrogenase
2740	-2.85	6.48E-08	*iolG*	oxidoreductase family NAD-binding Rossmann fold protein
85	-2.85	4.52E-07	*rpsJ*	30S ribosomal protein S10
891	-2.85	9.52E-09	*pepS*	aminopeptidase PepS
2039	-2.84	3.16E-09	*rihB*	inosine-uridine preferring nucleoside hydrolase
733	-2.83	2.29E-05	*fruA1*	PTS system fructose-specific transporter subunit IIA
1971	-2.83	4.00E-08	*mntH*	metal ion transporter,Probable manganese transport protein MntH
34	-2.83	1.95E-05	*ganB*	arabinogalactan endo-beta-galactosidase
2466	-2.81	3.89E-06	*ugpA4*	ABC transporter permease
1482	-2.81	4.41E-06	-	M protein trans-acting positive regulator,Mga helix-turn-helix domain
1162	-2.80	4.06E-09	*pflB*	formate acetyltransferase
1438	-2.80	3.31E-08	*hmpT*	Substrate-specific component PdxU2 of predicted pyridoxin-related ECF transporter
1077	-2.80	1.15E-08	*gmuC4*	PTS system lactose/cellobiose-specific transporter subunit IIC
915	-2.79	1.18E-08	*tagF*	utative glycosyl/glycerophosphate transferases involved in teichoic acid biosynthesis TagF/TagB/EpsJ/RodC
1078	-2.79	1.61E-08	*bglB*	6-phospho-beta-glucosidase
2859	-2.79	1.86E-08	*ebgA*	beta-galactosidase subunit alpha
759	-2.78	4.32E-08	*dltA*	D-alanine-poly(phosphoribitol) ligase subunit 1

2741	-2.78	2.82E-08	*fucO*	aldehyde-alcohol dehydrogenase
2244	-2.78	1.16E-05	-	quinolone resistance protein,secreted effector protein PipB
1932	-2.77	2.32E-07	-	Oligosaccharide biosynthesis protein Alg14 like
2330	-2.77	1.60E-04	-	hypothetical protein
1437	-2.77	1.28E-08	*thiD2*	phosphomethylpyrimidine kinase,Pyridoxine kinase
1612	-2.77	3.58E-06	*lacE2*	lactose ABC superfamily ATP binding cassette transporter
1394	-2.76	4.58E-07	*pyrK*	dihydroorotate dehydrogenase electron transfer subunit
2169	-2.76	1.27E-08	*yumC*	ferredoxin-NADP reductase
2040	-2.75	8.93E-09	-	integral membrane protein,Protein of unknown function (DUF3816)
1448	-2.75	7.67E-08	*ytgP1*	polysaccharide biosynthesis family protein,Probable cell division protein ytgP
437	-2.74	5.23E-09	-	sensor histidine kinase,sensory histidine kinase DcuS
1599	-2.73	2.24E-07	-	glyoxalase family protein,Glyoxalase-like domain
87	-2.70	1.18E-08	*rplD*	50S ribosomal protein L4
88	-2.70	1.31E-08	*rplW*	50S ribosomal protein L23
2900	-2.70	7.52E-06	-	hypothetical protein
2860	-2.68	1.79E-07	*hyl*	hyaluronate lyase
1624	-2.64	7.14E-09	*lacL*	beta-galactosidase large subunit
1533	-2.64	1.68E-05	-	
758	-2.63	7.95E-06	-	D-Ala-teichoic acid biosynthesis protein.,D-Ala-teichoic acid biosynthesis protein
422	-2.62	1.31E-08	-	peptide ABC transporter permease/transmembrane protein,FtsX-like permease family
3107	-2.61	3.36E-08	*walK2*	sensor histidine kinase,sensory histidine kinase DcuS
1261	-2.60	1.01E-05	-	lipase/acylhydrolase domain-containing protein
90	-2.60	2.50E-09	*rpsS*	SSU ribosomal protein S19P,30S ribosomal protein S19,30S ribosomal protein S19,Ribosomal protein S19,ribosomal protein S19,Ribosomal protein S19
1616	-2.60	2.23E-06	*pheA*	prephenate dehydratase
1615	-2.59	4.32E-08	*yehU2*	sensor histidine kinase
1450	-2.58	4.57E-08	*ygaZ*	putative azaleucine resistance protein AzlC
89	-2.57	2.38E-09	*rplB*	50S ribosomal protein L2
1541	-2.57	6.38E-05	*gldA1*	Glycerol dehydrogenase
438	-2.56	2.24E-08	-	hypothetical protein
2483	-2.56	9.04E-09	-	ABC transporter ATP-binding protein
253	-2.55	1.59E-04	-	CitMHS family citrate-magnesium (Mg2+):proton (H+) citrate-calcium (Ca2+): proton (H+) symporter
1264	-2.55	4.78E-08	-	DNA polymerase III subunit delta
2376	-2.55	6.03E-07	-	transposase
754	-2.55	2.49E-08	-	anaerobic ribonucleoside-triphosphate reductase activating protein
2381	-2.55	3.89E-08	*yfnB2*	HAD superfamily hydrolase
1410	-2.55	5.96E-08	*topA2*	DNA topoisomerase TopA
1739	-2.54	4.92E-06	*fhuB*	ferrichrome transport system permease FhuB
939	-2.54	2.53E-08	-	Protein of unknown function (DUF1189)
2474	-2.53	8.49E-09	*licR5*	BglG family transcriptional antiterminator
2774	-2.53	2.88E-09	*rpoC*	DNA-directed RNA polymerase

2473	-2.53	8.20E-07	*lacF4*	PTS system cellobiose-specific IIA componenent
8	-2.52	1.73E-08	*ssb1*	single-stranded DNA-binding protein,Helix-destabilizing protein
1238	-2.52	9.72E-08	*xylH*	amino acid or sugar ABC transport system, permease protein
1358	-2.51	4.56E-09	*ponA1*	penicillin-binding protein 1A
1039	-2.50	4.87E-04	*ulaG*	L-ascorbate 6-phosphate lactonase
1627	-2.50	4.12E-07	*aes2*	Acetyl esterase
84	-2.50	2.91E-09	*tufA*	protein-synthesizing GTPase,Elongation factor Tu
7	-2.49	3.84E-08	*rpsF*	30S ribosomal protein S6
337	-2.49	1.67E-05	*ulaA1*	PTS system ascorbate-specific transporter subunit IIC
1395	-2.49	7.57E-07	*pyrDB*	Dihydroorotate dehydrogenase 1B
581	-2.49	1.46E-08	*prsA1*	putative foldase protein PrsA
1447	-2.49	1.22E-07	*degV*	EDD domain-containing protein, DegV family
1614	-2.48	3.04E-07	*ypdC1*	Two-component response regulator TrxR
2451	-2.48	8.50E-06	-	sugar isomerase,L-xylulose 5-phosphate 3-epimerase
755	-2.47	2.32E-09	*nrdD*	anaerobic ribonucleoside triphosphate reductase
1621	-2.47	4.57E-07	*aroB*	3-dehydroquinate synthase
2457	-2.47	3.57E-08	*mngB*	glycosyl hydrolase family 38 protein
916	-2.47	3.67E-08	*rfbX1*	Membrane protein involved in the export of O-antigen and teichoic acid
2819	-2.46	2.11E-08	*trmE*	tRNA modification GTP-binding protein TrmE
2792	-2.46	1.94E-08	*yhbU2*	peptidase U32 family,Uncharacterized protease yhbU precursor
1314	-2.46	2.28E-08	*fabI*	Enoyl-[acyl-carrier-protein] reductase
235	-2.46	1.24E-03	-	TRAP dicarboxylate transporter subunit DctQ,2,3-diketo-L-gulonate TRAP transporter small permease protein YiaM
92	-2.45	4.68E-09	*rpsC*	30S ribosomal protein S3
2331	-2.45	7.54E-08	*feoB*	ferrous iron transport protein B,Ferrous iron transport protein B,ferrous iron transport protein B,Fe2+ transport system protein B,ferrous iron transport protein B,Ferrous iron transport protein B
1804	-2.43	8.53E-03	-	hypothetical protein
2472	-2.43	2.36E-07	-	hypothetical protein
1711	-2.43	5.59E-09	*rpsB*	30S ribosomal protein S2
735	-2.43	5.28E-07	*kbaY*	fructose-bisphosphate aldolase
1537	-2.43	1.33E-04	-	hypothetical protein
1927	-2.43	1.56E-06	-	hypothetical protein
1618	-2.42	1.04E-07	*aroA*	3-phosphoshikimate 1-carboxyvinyltransferase
3108	-2.42	1.16E-07	*walR*	Response regulators consisting of a CheY-like receiver domain and a winged-helix DNA-binding domain
1954	-2.42	4.58E-06	-	transposase
2465	-2.41	2.92E-06	*ycjP6*	ABC transporter permease,Inner membrane ABC transporter permease protein ycjP
2789	-2.41	1.74E-07	-	prenyltransferase, UbiA family,1,4-dihydroxy-2-naphthoate octaprenyltransferase
911	-2.41	4.68E-09	*wcaJ1*	Undecaprenyl-phosphate galactosephosphotransferase,Putative colanic biosynthesis UDP-glucose lipid carrier transferase
2447	-2.41	3.95E-06	*uxaB*	mannitol dehydrogenase protein,Altronate oxidoreductase

922	-2.40	1.60E-08	-	O-Antigen ligase
205	-2.40	3.25E-09	*ddl*	D-alanine-D-alanine ligase
2885	-2.40	1.66E-05	-	hypothetical protein
300	-2.39	9.95E-08	*sstT*	transporter, dicarboxylate/amino acid:cation symporter family protein,Na(+)/serine-threonine symporter
9	-2.39	2.09E-08	*rpsR*	SSU ribosomal protein S18P
1793	-2.38	2.75E-07	-	hypothetical protein
2824	-2.38	1.49E-07	*rpmH*	LSU ribosomal protein L34P
79	-2.37	8.33E-09	*hin1*	resolvase family site-specific recombinase,DNA-invertase hin,multiple promoter invertase,Resolvase, N terminal domain
91	-2.37	1.27E-08	*rplV*	50S ribosomal protein L22
2818	-2.37	1.38E-09	*gidA2*	tRNA uridine 5-carboxymethylaminomethyl modification protein GidA
421	-2.37	1.07E-06	*macB2*	ABC transporter ATP-binding protein,Macrolide export ATP-binding/permease protein MacB
299	-2.37	1.58E-06	-	lipoprotein
1656	-2.36	1.05E-08	*porA*	pyruvate flavodoxin oxidoreductase subunit alpha
1675	-2.36	2.59E-05	-	transposase
1534	-2.34	3.39E-05	*uxuA2*	mannonate dehydratase
2344	-2.34	2.08E-08	*rplJ*	50S ribosomal protein L10
1906	-2.34	2.00E-06	-	hypothetical protein
439	-2.34	1.23E-07	*agrB*	accessory regulator agrBfs protein
1449	-2.34	1.35E-06	-	branched-chain amino acid transport protein,Predicted membrane protein
1290	-2.32	4.04E-07	-	hypothetical protein
2343	-2.31	5.36E-08	*rplL*	50S ribosomal protein L7/L12
83	-2.31	1.86E-08	*fusA*	translation elongation factor G
2408	-2.30	2.38E-09	-	adenine-specific methyltransferase
109	-2.29	2.66E-09	*rpmJ*	LSU ribosomal protein L36P,Ribosomal protein II,50S ribosomal protein L36,ribosomal protein L36,Ribosomal protein L36
1041	-2.29	1.29E-05	*ulaA2*	PTS system ascorbate-specific transporter subunit IIC
1033	-2.28	1.17E-05	*gmuC3*	PTS system transporter subunit IIC
335	-2.28	2.23E-06	*ulaC1*	PTS system ascorbate-specific transporter subunit IIA
625	-2.28	1.94E-06	-	Mobile element protein,Transposase and inactivated derivatives
560	-2.28	1.54E-09	-	FeS assembly protein SufB,hypothetical protein
107	-2.28	5.67E-09	*adk*	adenylate kinase
557	-2.28	8.46E-09	*sufD*	FeS assembly protein SufD,hypothetical protein
2450	-2.28	3.87E-06	*rhgT*	GDSL-like Lipase/Acylhydrolase
120	-2.27	7.92E-08	*rpmB*	50S ribosomal protein L28
1613	-2.25	1.46E-06	-	response regulator receiver domain-containing protein
913	-2.25	2.39E-08	*hyaD1*	glycosyltransferase,Hyaluronan synthase
81	-2.25	5.18E-09	*rpsL*	30S ribosomal protein S12
2407	-2.25	9.75E-10	*ackA*	acetate kinase
991	-2.25	2.25E-07	*ybbH1*	bifunctional RpiR family transcriptional regulator/sugar isomerase
229	-2.25	1.97E-08	*tgt*	tRNA-guanine transglycosylase

914	-2.25	7.70E-08	*hyaD2*	Glycosyltransferases involved in cell wall biogenesis
2	-2.25	8.46E-09	*dnaB1*	DNA polymerase III subunit beta
2744	-2.25	1.69E-08	*iolB*	5-deoxy-glucuronate isomerase
2861	-2.25	2.82E-06	-	PEP phosphonomutase family protein
631	-2.25	5.19E-05	-	transposase
234	-2.25	6.78E-05	-	TRAP dicarboxylate transporter subunit DctP
644	-2.24	2.23E-08	*rbsR2*	LacI family transcriptional regulator,Ribose operon repressor
1617	-2.24	2.47E-05	*aroK*	shikimate kinase
2353	-2.23	2.29E-07	*secG*	Preprotein translocase subunit SecE
1477	-2.23	1.02E-06	-	MerR family transcriptional regulator,Glyoxalase-like domain
420	-2.22	3.47E-06	-	conserved hypothetical protein TIGR01655
1443	-2.22	6.36E-05	*aacA-aphD*	6'-aminoglycoside N-acetyltransferase,Predicted phosphotransferase related to Ser/Thr protein kinases
1146	-2.22	1.00E-05	*folT1*	membrane protein,Folate ECF transporter S component FolT
1210	-2.21	3.38E-04	*yesO2*	ABC superfamily ATP binding cassette transporter
339	-2.21	5.87E-05	*fba1*	Fructose-bisphosphate aldolase
108	-2.21	1.01E-08	*infA*	initiation factor IF-1
1905	-2.21	1.18E-06	-	hypothetical protein
1561	-2.21	9.35E-04	*srlA*	PTS system glucitol/sorbitol-specific EIIC component
96	-2.21	2.37E-09	*rplN*	50S ribosomal protein L14
93	-2.20	4.68E-09	*rplP*	LSU ribosomal protein L16P
2771	-2.19	1.02E-03	-	extracellular solute-binding protein,glycerol-3-phosphate transporter periplasmic binding protein,Maltose-binding periplasmic proteins/domains,carbohydrate ABC transporter substrate-binding protein, CPR_0540 family,Bacterial extracellular solute-binding protein
2775	-2.18	9.75E-10	*rpoB*	DNA-directed RNA polymerase subunit beta
558	-2.18	4.65E-09	*csd*	cysteine desulfurase
3105	-2.18	2.12E-07	*vanH2*	oxidoreductase, aldo/keto reductase family protein
94	-2.18	2.46E-08	*rpmC*	LSU ribosomal protein L29P
559	-2.18	7.79E-09	*nifU*	NifU family SUF system FeS assembly protein
1532	-2.18	2.38E-06	-	hypothetical protein
1592	-2.18	5.64E-09	*murC*	UDP-N-acetylmuramate--L-alanine ligase
110	-2.18	4.29E-09	*rpsM*	30S ribosomal protein S13,BS14
1359	-2.17	1.03E-07	*recU*	recombination protein U,Holliday junction resolvase recU
2781	-2.17	1.07E-08	-	cell wall surface adhesion protein,choice-of-anchor A domain
1	-2.17	1.03E-08	*dnaA1*	chromosomal replication initiation protein, DnaA
97	-2.17	4.81E-09	*rplX*	50S ribosomal protein L24
1933	-2.17	1.71E-07	*ugd*	UDP-glucose 6-dehydrogenase
2196	-2.17	1.07E-08	-	C4-dicarboxylate anaerobic carrier
232	-2.17	8.45E-05	*yesR*	Glycosyl Hydrolase Family 88,Unsaturated rhamnogalacturonyl hydrolase YesR
1539	-2.17	1.80E-04	*manZ2*	PTS system mannose-specific EIID component
2448	-2.16	4.23E-06	*garD*	altronate hydrolase,D-galactarate dehydratase
1538	-2.16	3.44E-04	*sorB*	Sorbose-specific phosphotransferase enzyme IIB component

1710	-2.16	1.73E-09	*tsf*	elongation factor EF1B
1931	-2.16	9.49E-06	-	Glycosyltransferase family 28 C-terminal domain
2742	-2.15	5.47E-07	*iolA2*	methylmalonate-semialdehyde dehydrogenase
2754	-2.15	3.23E-03	-	Transposase
82	-2.14	2.92E-07	*rpsG*	SSU ribosomal protein S7P
336	-2.14	8.56E-04	*ulaB1*	PTS system ascorbate-specific transporter subunits IICB
98	-2.14	6.31E-09	*rplE*	LSU ribosomal protein L5P
1262	-2.14	6.75E-07	-	ComE operon protein 2,tRNA-specific adenosine deaminase,Pyrimidine deaminase
2118	-2.14	2.12E-06	*ugl2*	glycosyl hydrolase family protein
1563	-2.14	1.56E-05	*licR3*	PRD domain-containing protein,Probable licABCH operon regulator
1930	-2.13	8.63E-06	-	glycosyl transferase family protein,Glycogen synthase
646	-2.12	2.91E-08	-	hypothetical protein
2655	-2.12	1.86E-08	*manX6*	PTS system mannose-specific transporter subunits IIAB
1929	-2.12	8.91E-07	*kfoC3*	glycosyl transferase family protein,rhamnosyltransferase
374	-2.12	1.86E-06	*dhaM*	PTS system glycerone kinase transporter subunit IIA
2791	-2.11	2.09E-08	*yhbU1*	U32 family peptidase,Uncharacterized protease yhbU precursor
732	-2.11	1.50E-08	*rpmE2*	50S ribosomal protein L31
556	-2.10	4.78E-07	*sufC*	ABC superfamily ATP binding cassette transporter
1803	-2.10	5.19E-04	-	hypothetical protein
1727	-2.10	7.67E-08	*degA2*	LacI family transcriptional regulator,Degradation activator
1820	-2.09	1.32E-04	*crr1*	Glucose-specific phosphotransferase enzyme IIA component,PTS system
912	-2.08	2.18E-08	*pgaC2*	glycosyl transferase family protein,rhamnosyltransferase
1003	-2.08	4.98E-07	*rpmF1*	LSU ribosomal protein L32P
2630	-2.08	6.93E-06	*ywnA*	Rrf2 family protein,Putative HTH-type transcriptional regulator ywnA
95	-2.08	1.07E-08	*rpsQ*	30S ribosomal protein S17,BS16
1990	-2.08	7.60E-07	-	Mobile element protein,Transposase and inactivated derivatives
111	-2.08	4.76E-08	*rpsK*	SSU ribosomal protein S11P
375	-2.08	1.63E-06	*dhaK*	dihydroxyacetone kinase subunit DhaK
2117	-2.08	2.15E-05	-	Uncharacterized protein conserved in bacteria
2468	-2.08	7.79E-09	*ytgP2*	polysaccharide biosynthesis family protein,Probable cell division protein ytgP
2471	-2.07	2.02E-08	*gmuC7*	PTS system cellobiose-specific transporter subunit IIC
1026	-2.07	1.79E-03	-	BglG family transcriptional antiterminator
1263	-2.07	1.71E-06	-	DNA internalization-related competence protein ComEC/Rec2
106	-2.06	1.66E-09	*secY*	preprotein translocase subunit SecY
1040	-2.06	1.69E-03	*cmtB*	PTS system ascorbate-specific transporter subunit IIA
1928	-2.06	4.80E-07	*cysE1*	Serine O-acetyltransferase
534	-2.06	4.96E-07	-	MFS family major facilitator transporter,enterobactin exporter EntS,H+ Antiporter protein,Major Facilitator Superfamily
1459	-2.06	9.55E-04	*nupC1*	Na dependent nucleoside transporter, NupC family
1034	-2.05	2.28E-05	*dgk*	diacylglycerol kinase

1499	-2.05	2.20E-05	-	ASCH protein,ASCH domain
1044	-2.05	2.13E-05	*sgbU*	hexulose-6-phosphate isomerase
102	-2.05	3.32E-09	*rplR*	50S ribosomal protein L18
1743	-2.05	4.80E-07	*rpmGA*	LSU ribosomal protein L33P
767	-2.05	1.93E-07	-	sensor histidine kinase
1878	-2.04	4.10E-07	-	hypothetical protein
99	-2.04	9.61E-09	*rpsZ*	30S ribosomal protein S14 type Z,BS21
112	-2.04	6.59E-09	*rpoA*	DNA-directed RNA polymerase subunit alpha
2391	-2.04	5.44E-08	*hisS*	histidyl-tRNA synthetase
3104	-2.03	1.80E-07	*vanB2*	D-alanine-D-lactate ligase,Vancomycin/teicoplanin A-type resistance protein VanA
1415	-2.03	1.83E-05	*chbR*	AraC family transcriptional regulator,Chb operon repressor
113	-2.03	4.05E-08	*rplQ*	50S ribosomal protein L17,BL21
1376	-2.03	2.25E-08	*alaS*	alanyl-tRNA synthetase
645	-2.02	8.82E-09	*gmuD2*	putative 6-phospho-beta-glucosidase
2656	-2.02	1.12E-08	*frlB2*	sugar isomerase,Fructosamine deglycase frlB
3024	-2.02	5.19E-03	*cca2*	CCA-adding enzyme
2354	-2.02	2.66E-05	*rpmG2*	LSU ribosomal protein L33P
1105	-2.02	8.57E-09	-	hypothetical protein
105	-2.01	2.66E-09	*rplO*	LSU ribosomal protein L15P
268	-2.01	6.59E-09	*pepA*	glutamyl aminopeptidase,Glutamyl aminopeptidase,exoaminopeptidase
1106	-2.00	1.56E-07	*yitL*	S1 domain RNA-binding protein,Conserved virulence factor B
529	-2.00	5.91E-09	*pepO*	Peptidase family M13,Neutral endopeptidase
1821	-2.00	3.49E-06	*sacX*	PTS system trehalose(maltose)-specific transporter subunits IIBC
376	-1.98	4.37E-06	*dhaL*	dihydroxyacetone kinase DhaL subunit
104	-1.97	3.86E-08	*rpmD*	50S ribosomal protein L30
338	-1.97	4.17E-04	*inference=ab initio prediction:Prodigal:2.60*	hypothetical protein
1536	-1.97	4.79E-04	*inference=ab initio prediction:Prodigal:2.60*	hypothetical protein
2543	-1.97	2.66E-08	*hslO*	chaperonin HslO,Heat shock protein 33 homolog
206	-1.96	3.48E-08	*murF*	UDP-N-acetylmuramoyl-tripeptide-D-alanyl-D- alanine ligase
1825	-1.96	7.50E-06	-	ISEnfa3, transposase
3068	-1.96	2.54E-05	*repB2*	plasmid replication initiation protein
1625	-1.96	4.43E-09	*galA2*	alpha-galactosidase
2542	-1.95	7.02E-09	*dus*	tRNA-dihydrouridine synthase

2140	-1.95	7.95E-09	*valS*	valyl-tRNA synthetase
2745	-1.95	1.81E-07	*iolD*	acetolactate synthase
1428	-1.94	6.97E-04	*lolD2*	ABC transporter ATP-binding protein
1757	-1.94	1.22E-04	-	hypothetical protein
2075	-1.94	9.61E-09	*traL*	putative type IV conjugative transfer system protein TraL
1138	-1.93	5.28E-08	-	dihydrofolate reductase
1494	-1.93	2.56E-08	*ftsW2*	FtsW/RodA/SpoVE family cell cycle protein
3054	-1.93	9.59E-06	*bin33*	resolvase,Putative transposon Tn552 DNA-invertase bin3
161	-1.93	1.29E-07	*nirC*	FNT family formate-nitrite transporter
100	-1.92	1.72E-08	*rpsH*	SSU ribosomal protein S8P
923	-1.92	1.54E-08	*galE*	UDP-glucose 4-epimerase
2452	-1.92	1.98E-06	-	Putative multidrug export ATP-binding/permease protein SAV1866
2010	-1.92	1.30E-08	*licB3*	PTS system cellobiose-specific transporter subunit IIB
103	-1.92	4.29E-09	*rpsE*	SSU ribosomal protein S5P
1535	-1.91	5.68E-04	*manX3*	PTS system mannose-specific transporter subunits IIAB
2772	-1.91	2.03E-04	-	alpha amylase,Sucrose phosphorylase
909	-1.91	2.81E-08	*kfoC2*	glycosyl transferase family protein,Chondroitin polymerase
1775	-1.90	6.91E-07	-	HAD superfamily hydrolase,sugar phosphate phosphatase
46	-1.90	5.48E-07	-	Protein of unknown function (DUF3644)
1234	-1.90	2.19E-08	*yehU1*	Probable sensor-like histidine kinase YehU
2223	-1.89	1.18E-04	-	hypothetical protein
1423	-1.88	8.60E-04	-	RpiR family transcriptional regulator,Helix-turn-helix domain
1136	-1.88	4.19E-07	*yjjK1*	ABC transporter ATP-binding protein
712	-1.88	5.18E-09	*luxO1*	transcriptional antiterminator,Regulatory protein luxO
1090	-1.87	1.62E-08	*pksG*	hydroxymethylglutaryl-CoA synthase,Polyketide biosynthesis 3-hydroxy-3-methylglutaryl-ACP synthase pksG
2622	-1.87	7.14E-09	-	mannosyl-glycoprotein endo-beta-N-acetylglucosaminidase
906	-1.86	7.13E-08	-	ABC-2 type transporter
2234	-1.85	1.17E-07	*rlmN*	ribosomal RNA large subunit methyltransferase N
1137	-1.85	2.04E-06	*thyB*	thymidylate synthase
2121	-1.85	9.42E-04	-	Bacterial extracellular solute-binding protein,Maltose-binding periplasmic proteins/domains
2779	-1.85	4.48E-08	*birA*	bifunctional biotin--[acetyl-CoA-carboxylase] ligase/biotin operon transcriptional regulator
1926	-1.84	1.10E-05	-	polysaccharide biosynthesis protein CpsM,Mannosyltransferase OCH1 and related enzymes
1865	-1.84	2.08E-06	-	putative DNA-binding protein
1728	-1.84	5.75E-08	-	galactose-1-phosphate uridylyltransferase
2209	-1.84	1.34E-07	*yheI2*	ABC transporter ATP-binding protein/permease,Probable multidrug resistance ABC transporter ATP-binding/permease protein YheI
344	-1.84	3.13E-08	-	B3/4 domain-containing protein,phenylalanine-tRNA ligase
2755	-1.84	2.99E-05	-	Transposase IS116/IS110/IS902 family
2701	-1.83	3.26E-06	-	hypothetical protein
1686	-1.83	6.83E-07	*proX1*	YbaK/prolyl-tRNA synthetase-associated domain protein

2910	-1.83	1.71E-08	*gmuC8*	PTS system oligo-beta-mannoside-specific EIIC component
1213	-1.83	2.31E-04	-	YkoY family integral membrane protein
3025	-1.83	1.11E-03	-	hypothetical protein
2390	-1.82	4.90E-08	*aspS*	aspartyl-tRNA synthetase
1214	-1.82	4.75E-05	*idhA1*	putative oxidoreductase
1560	-1.81	4.75E-05	*srlE*	PTS system glucitol/sorbitol-specific transporter subunit IIBC
1087	-1.81	1.99E-08	-	calcium-translocating P-type ATPase
1729	-1.81	2.81E-08	*galE3*	UDP-glucose 4-epimerase
1943	-1.81	2.14E-07	*glyQ*	glycyl-tRNA synthetase subunit alpha
1637	-1.81	3.68E-07	*clcA1*	voltage gated cchloride transporter, chloride channel family protein,H(+)/Cl(-) exchange transporter ClcA
3106	-1.81	3.90E-06	-	Mobile element protein,Transposase
101	-1.81	1.94E-08	*rplF*	50S ribosomal protein L6,BL10
2757	-1.80	2.39E-08	*rpsI2*	30S ribosomal protein S9
505	-1.80	1.12E-01	-	hypothetical protein
2064	-1.80	8.41E-09	*ntpD*	V-type ATP synthase subunit D
1860	-1.80	6.52E-08	*rpsP*	30S ribosomal protein S16,BS17
908	-1.80	3.85E-08	*metW*	methionine biosynthesis protein MetW,16S ribosomal RNA methyltransferase KsgA/Dim1 family protein
1212	-1.79	5.04E-05	*malG*	ABC transporter permease,Maltose transport system permease protein malG
1130	-1.77	7.43E-06	-	YitT family protein
2868	-1.77	1.02E-04	*hssS*	sensor histidine kinase
1963	-1.77	1.68E-05	*chbF*	alpha-galactosidase
2558	-1.77	2.13E-07	-	NlpC/P60 family lipoprotein,invasion associated secreted endopeptidase,Membrane-bound metallopeptidase
179	-1.76	6.30E-04	-	integral membrane protein,Protein of unknown function (DUF1211)
2785	-1.76	5.78E-06	-	Mobile element protein,Transposase
2109	-1.75	1.66E-05	*ecsA4*	ABC transporter ATP-binding protein
436	-1.75	8.99E-08	*agrA1*	response regulator receiver domain-containing protein
162	-1.75	2.57E-08	-	DNA alkylation repair protein
1918	-1.74	3.46E-08	*yidC*	putative OxaA-like protein,Membrane protein YidC
2873	-1.74	2.36E-04	*panC*	pantoate-beta-alanine ligase
1942	-1.73	5.84E-08	*glyS*	glycyl-tRNA synthetase subunit beta
1211	-1.73	1.18E-04	*ycjO1*	ABC transporter permease
1216	-1.73	5.36E-08	*xpt*	xanthine phosphoribosyltransferase
837	-1.72	4.95E-07	-	helix-turn-helix domain-containing protein
2758	-1.72	1.09E-08	*rplM*	50S ribosomal protein L13
2746	-1.71	2.11E-07	*iolC*	5-dehydro-2-deoxygluconokinase
1102	-1.71	2.24E-08	-	P-ATPase superfamily cation transporter,Calcium-transporting ATPase
1953	-1.70	2.21E-05	-	ISEnfa3, transposase,
2076	-1.70	1.44E-07	*ecsA3*	ABC transporter ATP-binding protein
905	-1.70	2.88E-08	-	membrane protein,Lipid A core - O-antigen ligase and related enzymes
1546	-1.70	4.18E-07	-	hypothetical protein

1735	-1.69	1.10E-03	*yqgQ*	DUF910 family protein,hypothetical protein
736	-1.69	3.87E-06	*glcR*	DeoR family transcriptional regulator,HTH-type transcriptional repressor glcR
1088	-1.69	6.59E-09	-	calcium-translocating P-type ATPase,,magnesium-transporting ATPase MgtA
1295	-1.69	6.12E-08	*dtpT*	amino acid/peptide transporter,Di-/tripeptide transporter
343	-1.67	6.30E-08	*paiA*	GNAT family acetyltransferase,Protease synthase and sporulation negative regulatory protein PAI 1
2152	-1.67	3.30E-08	*pepC*	Aminopeptidase C
3103	-1.67	1.69E-06	*vanX2*	D-alanyl-D-alanine dipeptidase
2479	-1.67	2.32E-07	-	Cna B-type,Predicted outer membrane protein
3023	-1.67	5.25E-04	-	hypothetical protein
1577	-1.66	3.95E-06	-	membrane protein,Protein of unknown function (DUF1294)
1925	-1.66	2.14E-05	*tagE*	group 1 glycosyl transferase,Probable poly(glycerol-phosphate) alpha-glucosyltransferase
250	-1.66	1.68E-07	*rpmI*	50S ribosomal protein L35
2122	-1.66	2.81E-07	*adaA*	methyltransferase,ubiquinone/menaquinone biosynthesis methyltransferase
1148	-1.66	3.07E-07	*ylqF*	ribosome biogenesis GTP-binding protein
2347	-1.66	1.46E-07	*rplK*	50S ribosomal protein L11
152	-1.65	3.76E-07	*gla1*	glycerol uptake facilitator protein,Glyceroaquaporin
926	-1.65	2.54E-07	-	ISEnfa3, transposase
907	-1.65	3.67E-08	*tagH*	ABC transporter ATP-binding protein,Teichoic acids export ATP-binding protein TagH
2449	-1.65	1.88E-06	*uxaC*	glucuronate isomerase
2541	-1.64	1.05E-08	*lysS*	lysyl-tRNA synthetase
1859	-1.64	2.99E-07	-	RNA-binding protein
1542	-1.64	2.57E-04	-	alcohol dehydrogenase
2882	-1.63	2.43E-07	*macB2*	macrolide-specific efflux protein MacB
2821	-1.62	8.22E-08	-	RNA-binding protein,R3H domain
1083	-1.62	1.07E-02	-	hypothetical protein
1685	-1.62	2.11E-07	*ldcA*	Murein tetrapeptide carboxypeptidase
2862	-1.62	2.75E-07	-	ISEnfa3, transposase
1119	-1.62	9.01E-06	-	ferredoxin
1970	-1.62	7.66E-07	*yneF*	protein YneF,hypothetical protein
1092	-1.61	4.09E-07	-	hypothetical protein
1962	-1.61	6.71E-06	-	
904	-1.61	1.96E-07	-	YkoY family integral membrane protein
3040	-1.61	2.36E-07	-	ISEnfa3, transposase
1966	-1.61	8.36E-04	*bglG1*	transcriptional antiterminator
1045	-1.61	1.24E-04	*araD*	L-ribulose-5-phosphate 4-epimerase
2445	-1.60	1.38E-05	*ypdC2*	AraC family transcriptional regulator
2065	-1.60	2.65E-08	*ntpB*	V-type ATP synthase subunit B
251	-1.60	9.10E-08	*rplT1*	50S ribosomal protein L20
2480	-1.60	1.53E-04	-	putative site-specific integrase-resolvase
836	-1.60	1.02E-05	-	putative Zn peptidase,Domain of unknown function (DUF955)
249	-1.60	8.56E-08	*infC*	translation initiation factor IF-3

1824	-1.60	6.45E-07	-	ISEnfa3, transposase
2108	-1.60	6.15E-06	-	ABC superfamily ATP binding cassette transporter, membrane protein
1570	-1.59	1.16E-02	*xerC6*	Phage integrase,Tyrosine recombinase XerC
1042	-1.59	4.64E-04	*ulaB2*	PTS system ascorbate-specific transporter subunit IIB
1576	-1.59	1.19E-06	-	integral membrane protein,conserved hypothetical protein
2497	-1.59	2.64E-03	-	putative holin
1107	-1.59	4.73E-07	*fur*	ferric (Fe) uptake regulator
1397	-1.59	2.66E-05	*pyrE*	
1807	-1.58	1.18E-07	-	ISEnfa3, transposase
2572	-1.58	4.20E-07	-	ISEnfa3, transposase
750	-1.58	1.66E-07	*yaaT*	stage 0 sporulation protein YaaT
2928	-1.58	2.52E-08	*bglF6*	PTS system, sucrose-specific IIA, IIB, IIC component
78	-1.58	6.75E-08	gpmA1	phosphoglycerate mutase
1738	-1.58	7.35E-06	*fhuG*	ferrichrome transport system permease FhuG
2294	-1.57	1.50E-06	*araR*	GntR family transcriptional regulator,Arabinose metabolism transcriptional repressor
647	-1.57	2.51E-08	*ltaS11*	sulfatase,Lipoteichoic acid synthase 1
1158	-1.57	4.09E-07	*plsY*	acyl-phosphate glycerol 3-phosphate acyltransferase
2557	-1.57	2.19E-06	*pepF1*	oligoendopeptidase PepF/M3 family
2822	-1.56	5.41E-08	*misCA*	pheromone cCF10 percursor/lipoprotein, 60 kDa,Stage III sporulation protein J
1043	-1.56	4.10E-05	*sgbH*	3-dehydro-L-gulonate-6-phosphate decarboxylase
15	-1.56	1.81E-07	*ykpA*	ABC transporter ATP-binding protein
1167	-1.56	1.49E-06	-	integral membrane protein,hypothetical protein
1871	-1.56	2.48E-07	-	hypothetical protein
2909	-1.56	2.82E-08	-	PEP phosphonomutase family protein
163	-1.55	2.65E-08	-	HAD superfamily hydrolase,Putative bifunctional phosphatase/peptidyl-prolyl cis-trans isomerase
1626	-1.55	4.99E-07	*ccpA2*	LacI family transcriptional regulator,Catabolite control protein
1866	-1.55	3.45E-06	-	DUF523 family protein,Uncharacterized conserved protein
1745	-1.55	5.74E-08	*asnB*	asparagine synthetase B
2116	-1.55	3.23E-06	*ugl1*	family 88 glycosyl hydrolase,Unsaturated glucuronyl hydrolase
2635	-1.55	6.15E-08	*azlC*	azaleucine resistance protein AzlC,Inner membrane protein YgaZ
817	-1.55	6.88E-08	*ileS*	isoleucyl-tRNA synthetase
1302	-1.55	8.13E-07	*pheT2*	phenylalanyl-tRNA synthetase subunit beta
1734	-1.54	1.84E-03	*glcK*	glucokinase
2823	-1.54	1.41E-07	*rnpA*	Ribonuclease P protein component
1544	-1.53	1.22E-03	*gnd2*	phosphogluconate dehydrogenase (decarboxylating)
903	-1.53	3.69E-07	*rmlD*	dTDP-4-dehydrorhamnose reductase
2467	-1.53	1.56E-07	*rluB2*	16S rRNA pseudouridylate synthase A
2071	-1.53	9.69E-09	*ntpI*	V-type ATP synthase subunit I
2504	-1.52	1.37E-02	-	ISEnfa3, transposase
2489	-1.52	1.77E-07	-	radical SAM protein family,coproporphyrinogen III oxidase
36	-1.52	1.78E-06	-	Transposase and inactivated derivatives

1321	-1.52	7.65E-07	*obg*	GTPase obg,Spo0B-associated GTP-binding protein
2356	-1.52	6.19E-07	*queT2*	integral membrane protein,Queuosine precursor ECF transporter S component QueT
401	-1.52	4.77E-06	-	PadR family transcriptional regulator
2067	-1.52	7.07E-08	*ntpG*	V-type ATP synthase subunit G
1233	-1.52	4.96E-07	-	AraC family transcriptional regulator
959	-1.51	2.13E-07	*ptsI*	phosphoenolpyruvate-protein phosphotransferase
1864	-1.51	5.56E-07	*ffh*	signal recognition particle subunit FFH/SRP54 (srp54)
2939	-1.51	2.50E-07	-	Uncharacterized conserved protein,secondary thiamine-phosphate synthase enzyme
1116	-1.51	1.05E-08	-	C4-dicarboxylate anaerobic carrier
1543	-1.50	2.37E-05	*serA2*	phosphoglycerate dehydrogenase
607	-1.50	7.04E-08	-	Helix-turn-helix domain protein
2068	-1.50	2.61E-08	*ntpC*	V-type ATPase subunit C,V-type sodium pump subunit C
2210	-1.50	1.78E-07	-	ABC transporter ATP-binding protein/permease,Putative multidrug export ATP-binding/permease protein SAV1866
1307	-1.49	1.13E-06	*artQ*	ABC transporter permease,Arginine transport system permease protein ArtQ
386	-1.49	1.75E-06	*tyrS1*	tyrosyl-tRNA synthetase
2120	-1.49	7.98E-04	*ugpA2*	ABC transporter permease,sn-glycerol-3-phosphate transport system permease protein ugpA
2463	-1.49	8.14E-04	-	integral membrane protein
340	-1.49	7.50E-04	-	Predicted kinase,Zeta toxin
2144	-1.49	3.25E-07	*iscS2*	pyridoxal phosphate-dependent transferase,Cysteine desulfurase
1643	-1.49	1.35E-04	-	hypothetical protein
2072	-1.48	7.38E-08	-	V-type ATP synthase subunit G
2069	-1.48	1.77E-07	-	V-type ATPase subunit E
1967	-1.48	4.81E-04	*bglG2*	transcriptional antiterminator
673	-1.47	1.25E-06	-	ABC transporter protein,ABC-type transport system involved in multi-copper enzyme maturation
1235	-1.47	6.24E-08	*ytfQ*	LacI family transcriptional regulator
2293	-1.47	2.23E-03	*csbC*	MFS transporter, sugar porter family protein
2712	-1.47	1.20E-02	-	ISEnfa3, transposase
1149	-1.47	2.11E-06	*rnhB*	ribonuclease HII
455	-1.47	2.37E-07	*efp*	translation elongation factor P
674	-1.47	2.61E-07	*ecsA2*	ABC transporter ATP-binding protein
2070	-1.46	5.98E-08	*ntpK*	proton (H+) or sodium (Na+) translocating V-type ATPase
1198	-1.46	5.19E-05	*dcuS2*	two-component sensor histidine kinase
1773	-1.46	1.07E-06	*srrA1*	response regulator receiver domain-containing protein
958	-1.45	1.69E-08	*ptsH*	phosphocarrier protein HPr
1217	-1.45	7.39E-08	*ygfU*	xanthine permease
618	-1.45	1.57E-03	-	ISEnfa3, transposase
2417	-1.45	1.94E-06	*trmB*	
1317	-1.44	7.86E-08	-	alpha3-beta1 integrin-binding family protein
1828	-1.44	3.23E-04	-	ISEnfa3, transposase
2884	-1.44	8.17E-05	-	IS1167 transposase
2941	-1.44	1.14E-06	*gatC3*	PTS system galactitol-specific transporter subunit IIC

2636	-1.44	1.07E-06	-	branched-chain amino acid transport protein,Predicted membrane protein
2940	-1.44	1.17E-06	-	PTS system galactitol-specific transporter subunit IIB
1924	-1.44	4.84E-06	*rfbX2*	PST family polysaccharide transporter,Polysaccharide biosynthesis protein
999	-1.43	6.12E-08	*pfkA*	6-phosphofructokinase
1296	-1.43	3.69E-03	-	transposase
1564	-1.43	2.95E-02	*fabG4*	short chain dehydrogenase/reductase family oxidoreductase
1183	-1.43	8.08E-06	-	cell wall protein,chromosome segregation protein
1880	-1.43	2.02E-02	-	ISEnfa3, transposase
169	-1.42	1.46E-06	*treB*	PTS system trehalose(maltose)-specific transporter subunits IIBC
391	-1.42	2.55E-06	*ccpA1*	LacI family transcriptional regulator,Catabolite control protein
236	-1.42	9.39E-04	*siaT*	TRAP dicarboxylate transporter subunit DctM,Neu5Ac permease,L-dehydroascorbate transporter large permease subunit
1910	-1.42	4.69E-08	*dacA*	D-alanyl-D-alanine carboxypeptidase
1558	-1.42	1.22E-03	-	PTS system glucitol/sorbitol-specific transporter subunit IIA
199	-1.42	6.09E-07	*udk*	uridine kinase
1456	-1.42	2.50E-05	-	hypothetical protein
1772	-1.42	1.78E-07	*phoR2*	sensor histidine kinase,Alkaline phosphatase synthesis sensor protein phoR
2945	-1.42	9.01E-06	-	hypothetical protein
2946	-1.42	2.11E-06	-	circular bacteriocin
1109	-1.42	1.49E-07	*xerD1*	site-specific recombinase XerD,Tyrosine recombinase
1429	-1.41	9.37E-05	-	efflux ABC transporter permease,FtsX-like permease family
1319	-1.41	4.55E-06	-	x-Pro dipeptidyl-peptidase,Thermostable monoacylglycerol lipase
707	-1.41	1.17E-04	*puuR*	helix-turn-helix domain-containing protein
788	-1.41	6.64E-04	-	transposase
160	-1.41	1.51E-06	*rpsD*	SSU ribosomal protein S4P
1774	-1.41	4.38E-05	-	integral membrane protein
392	-1.41	5.50E-05	*mro*	aldose 1-epimerase
2290	-1.41	2.62E-04	*araA2*	L-arabinose isomerase
1610	-1.41	2.95E-04	*ycjP2*	ABC transporter permease
1248	-1.41	2.42E-04	-	transposase
1396	-1.40	5.63E-06	*pyrF*	Orotidine 5'-phosphate decarboxylase
1446	-1.39	9.84E-07	-	hypothetical protein
2584	-1.39	7.34E-07	*purR2*	pur operon repressor
2066	-1.39	2.64E-06	*ntpA*	V-type ATP synthase subunit A
441	-1.39	6.69E-07	*dukA*	deoxynucleoside kinase
2346	-1.39	1.03E-06	*rplA*	50S ribosomal protein L1
2874	-1.38	1.09E-06	*panD*	aspartate alpha-decarboxylase
2195	-1.38	1.67E-06	*iadA*	beta-aspartyl peptidase
1301	-1.38	1.43E-06	*pheS2*	phenylalanyl-tRNA synthetase subunit alpha
2943	-1.37	7.76E-07	*licR6*	Transcriptional antiterminator
729	-1.37	1.51E-06	*fba2*	fructose-bisphosphate aldolase

1110	-1.36	1.78E-06	-	GNAT family acetyltransferase,ribosomal-protein-alanine acetyltransferase
293	-1.35	1.11E-07	*ermG*	rRNA adenine N-6-methyltransferase
1313	-1.35	1.46E-06	*ilvE*	branched-chain-amino-acid transaminase
1239	-1.35	1.38E-07	*guaC*	GMP reductase
231	-1.35	3.28E-07	*aad*	aldehyde-alcohol dehydrogenase
1818	-1.35	1.62E-04	*nifA*	PTS system transcriptional activator
1258	-1.35	2.31E-07	*ylbL*	S16 family peptidase
789	-1.35	5.64E-08	*pepQ1*	Xaa-Pro dipeptidase
1650	-1.35	5.48E-05	-	hypothetical protein
484	-1.34	9.35E-05	-	LysM domain-containing protein
2418	-1.34	8.44E-07	-	Phosphotransferase,thiamine kinase,Phosphotransferase enzyme family
804	-1.34	2.75E-07	*mraW*	S-adenosyl-methyltransferase MraW
2490	-1.34	1.02E-06	-	rRNA methylase family protein
2644	-1.33	4.18E-06	*arlR2*	response regulator receiver domain protein
2386	-1.32	6.09E-06	*yfiC*	methyltransferase small domain-containing protein
1700	-1.32	1.28E-07	*rimP*	ribosome maturation protein RimP
1557	-1.31	7.72E-04	*fsaA*	fructose-6-phosphate aldolase
2810	-1.31	7.27E-08	*serS2*	seryl-tRNA synthetase
1312	-1.31	2.53E-06	*mscS*	transporter, small conductance mechanosensitive ion channel family protein
805	-1.31	9.08E-07	*ftsL*	cell division protein
2143	-1.31	2.80E-07	*thiI*	thiamine biosynthesis protein ThiI
564	-1.31	2.86E-05	-	hypothetical protein
3102	-1.31	5.58E-06	-	D-alanyl-D-alanine carboxypeptidase
1498	-1.31	5.42E-04	*nanE*	N-acetylmannosamine-6-phosphate 2-epimerase
624	-1.30	4.32E-06	-	sensor histidine kinase
2348	-1.30	6.09E-07	*sdhB*	L-serine dehydratase iron-sulfur-dependent subunit beta
1339	-1.30	1.36E-03	-	transposase
802	-1.29	1.74E-03	-	transposase
1197	-1.29	1.69E-03	*dcuS1*	sensor histidine kinase
1111	-1.29	2.69E-07	*scpA*	segregation and condensation protein A
2270	-1.29	1.46E-02	-	ABC-2 family transporter protein
2488	-1.28	2.65E-06	*bcrC*	PAP2 family protein,Undecaprenyl-diphosphatase BcrC
2477	-1.28	2.16E-06	-	membrane protein
1365	-1.28	4.08E-07	*ypwA*	carboxypeptidase Taq metallopeptidase
2396	-1.27	1.38E-07	*relA2*	GTP diphosphokinase,Bifunctional (p)ppGpp synthase/hydrolase RelA
1112	-1.27	1.82E-06	*scpB*	segregation and condensation protein B
711	-1.27	2.98E-06	*potD*	spermidine/putrescine-binding periplasmic protein PotD
2366	-1.27	2.12E-05	*trmL*	SpoU family RNA methyltransferase
273	-1.27	6.15E-05	*rlmH*	ribosomal RNA large subunit methyltransferase H
1548	-1.27	3.60E-04	*fms7*	LPXTG family cell surface protein Fms7,Esterase/lipase,Alpha/beta hydrolase family
1736	-1.27	1.54E-03	*gluP*	S54 family peptidase,Rhomboid protease gluP
368	-1.26	2.88E-06	-	integral membrane protein

3021	-1.26	4.12E-04	-	Phage lysin,Mannosyl-glycoprotein endo-beta-N-acetylglucosaminidase
1733	-1.26	3.68E-05	*yibN*	rhodanese family protein,hypothetical protein
1257	-1.26	5.51E-05	*coaD*	Phosphopantetheine adenylyltransferase CoaD
1965	-1.26	2.24E-05	*bglF5*	PTS system beta-glucoside-specific IIABC component
1493	-1.26	5.50E-07	*psr*	LytR family transcriptional regulator
1151	-1.26	3.72E-07	*topA*	DNA topoisomerase TopA
1819	-1.25	7.72E-05	*gmuE1*	fructokinase
2395	-1.25	1.79E-07	*dtd*	D-tyrosyl-tRNA(Tyr) deacylase
1256	-1.25	9.48E-06	*rsmD*	RsmD family RNA methyltransferase
728	-1.25	1.51E-07	*pyrG*	CTP synthetase
2942	-1.25	1.14E-06	-	PTS system galactitol-specific transporter subunit IIA
170	-1.24	3.17E-07	*treR*	GntR family transcriptional regulator,Trehalose operon transcriptional repressor
316	-1.24	1.66E-04	*gmuC2*	PTS system lactose/cellobiose-specific transporter subunit IIC
487	-1.24	1.47E-06	*thiO*	FAD dependent oxidoreductase,Glycine oxidase
687	-1.24	6.04E-06	*ung*	uracil-DNA glycosylase
944	-1.24	4.18E-08	*lytR1*	transcriptional regulator,Membrane-bound protein lytR
990	-1.24	1.56E-07	*thrS*	threonyl-tRNA synthetase
1373	-1.22	1.32E-07	*cshB*	DEAD-box ATP dependent DNA helicase
1276	-1.22	2.13E-07	*yitW*	metal-sulfur cluster biosynthetic enzyme
546	-1.22	1.41E-06	*yeiH*	membrane protein
2063	-1.22	2.66E-08	*ntpJ*	V-type sodium ATP synthase subunit J
1647	-1.22	7.77E-06	-	hypothetical protein
3101	-1.22	8.03E-04	*vanZ2*	teicoplanin resistance protein VanZ
67	-1.21	4.29E-06	*degA1*	LacI family transcriptional regulator,Degradation activator
627	-1.21	1.55E-03	-	transposase
1445	-1.21	1.96E-07	*clpX1*	ATP-dependent protease ATP-binding subunit ClpX
954	-1.21	1.47E-06	*prfC*	peptide chain release factor 3
1115	-1.21	1.10E-07	*fmnP*	membrane protein,Riboflavin ECF transporter S component FmnP
1002	-1.21	2.68E-06	-	nucleic acid-binding protein,Uncharacterized ACR, COG1399
2509	-1.21	9.70E-06	*bmr31*	MFS family major facilitator transporter, drug resistance transporter
710	-1.21	1.48E-05	*potC*	spermidine/putrescine transport system permease protein PotC
2142	-1.20	4.39E-03	*rex2*	redox-sensing transcriptional repressor Rex
3065	-1.20	5.34E-06	*repB1*	plasmid replication initiation protein
1305	-1.20	4.34E-05	*peb1A*	putative adhesin/ABC transporter aspartate/glutamate-binding protein
2061	-1.20	2.57E-04	-	ornithine cyclodeaminase/mu-crystallin family protein
1298	-1.20	5.44E-08	*addA*	ATP-dependent helicase/nuclease subunit A
2411	-1.20	2.13E-02	-	Late competence protein ComGE
2456	-1.20	4.91E-06	-	fructokinase
2643	-1.20	1.98E-06	-	sensor histidine kinase
148	-1.20	5.98E-03	-	M protein trans-acting positive regulator,Mga helix-turn-helix domain

1822	-1.20	1.11E-04	-	glycoside hydrolase family 32
360	-1.19	2.83E-05	*gla2*	glycerol uptake facilitator protein,Glyceroaquaporin
803	-1.19	2.22E-06	*mraZ*	cell division protein MraZ
1648	-1.19	5.85E-07	-	RDD family protein
2361	-1.19	1.81E-05	-	sensor histidine kinase
1594	-1.19	2.14E-06	-	SprT-like family protein
1152	-1.19	1.72E-07	-	tRNA (uracil-5-)-methyltransferase
2201	-1.19	7.43E-06	-	ComE operon protein 2,tRNA-specific adenosine deaminase
233	-1.18	5.11E-02	-	TRAP dicarboxylate transporter subunit DctP
1347	-1.18	5.95E-02	-	recombination factor protein RarA
2713	-1.18	1.34E-04	-	ISEnfa3, transposase
1701	-1.18	5.99E-08	*polC2*	DNA polymerase III PolC-type
1219	-1.18	2.25E-07	-	phosphoribosylaminoimidazole carboxylase ATPase subunit
3042	-1.17	4.53E-06	-	hypothetical protein
1297	-1.17	1.83E-07	*addB*	ATP-dependent helicase/deoxyribonuclease subunit B
2211	-1.17	1.19E-06	*folT2*	integral membrane protein,Folate ECF transporter S component FolT
263	-1.17	7.17E-04	-	transposase
1571	-1.17	3.18E-07	*lepA*	GTP-binding protein LepA
1306	-1.16	2.90E-04	*glnP2*	ABC transporter permease,Glutamine transport system permease protein glnP,amino acid ABC transporter permease,ABC-type amino acid transport system, permease component,ectoine/hydroxyectoine ABC transporter, permease protein EhuD,Binding-protein-dependent transport system inner membrane component
2867	-1.16	1.90E-07	*mprA2*	DNA-binding response regulator
1460	-1.16	5.15E-05	*nupC2*	Na dependent nucleoside transporter
2723	-1.15	1.01E-01	*rpmF2*	50S ribosomal protein L32
1777	-1.15	1.35E-03	-	transposase
1433	-1.15	2.13E-05	-	DNA integrase,Integrase core domain
984	-1.15	2.36E-07	*yeeN*	conserved hypothetical protein TIGR01033,Probable transcriptional regulatory protein YeeN
1000	-1.15	7.58E-07	-	pyruvate kinase
2769	-1.15	4.70E-03	*ycjP7*	ABC transporter permease
342	-1.15	2.34E-07	*yybA*	MarR family transcriptional regulator
467	-1.15	8.20E-05	-	DNA integrase,Integrase core domain
129	-1.14	1.32E-05	*yejE*	ABC transporter permease
1699	-1.14	6.83E-08	*nusA*	transcription elongation factor NusA
660	-1.14	6.36E-05	*nhaC2*	Na+/H+ antiporter,Sodium/proton antiporter
1547	-1.14	1.56E-03	-	Acetyl esterase
752	-1.14	4.64E-06	-	tetrapyrrole methylase family protein
2012	-1.14	4.29E-05	*yigZ*	protein co-occurring with transport system
1697	-1.13	6.56E-07	-	50S ribosomal protein L7Ae,hypothetical protein,Ribosomal protein L30E,Ribosomal protein L7Ae/L30e/S12e/Gadd45 family
544	-1.13	3.05E-06	-	glycosyl transferase family protein
2387	-1.13	3.92E-05	-	GIY-YIG nuclease superfamily protein
237	-1.13	8.14E-03	-	ISEnfa3, transposase

1457	-1.13	6.41E-05	-	ATP-dependent Zn protease
2923	-1.12	1.06E-02	-	transposase, IS200 family
2925	-1.12	6.12E-06	-	DNA integrase,Integrase core domain
1492	-1.12	2.24E-07	-	penicillin binding protein transpeptidase domain protein
2478	-1.12	3.62E-07	*sdrD*	Cna B-type,Serine-aspartate repeat-containing protein D precursor
1879	-1.11	1.18E-03	-	ISEnfa3, transposase
2917	-1.11	1.92E-05	-	PTS system transporter subunit IIA
1737	-1.11	2.01E-03	-	5-formyltetrahydrofolate cyclo-ligase
370	-1.11	1.47E-06	-	glycosyl transferase family protein
1108	-1.11	1.70E-03	-	hypothetical protein
920	-1.10	6.57E-05	-	IS204/IS1001/IS1096/IS1165 transposase
2180	-1.10	3.94E-05	-	rRNA (cytosine-C(5)-)-methyltransferase
2336	-1.10	6.69E-07	*yhhT4*	pheromone autoinducer 2 transporter
806	-1.10	7.38E-07	-	peptidoglycan glycosyltransferase,Penicillin-binding protein 2X
1113	-1.10	8.79E-06	-	16S rRNA pseudouridine synthase A
1549	-1.10	2.12E-04	-	isochorismatase family hydrolase
2571	-1.10	3.02E-06	-	guanylate kinase
2613	-1.09	2.00E-06	-	hypothetical protein
80	-1.09	6.84E-07	-	ribose-5-phosphate isomerase
2959	-1.09	8.41E-07	-	putative transcriptional regulator
1351	-1.09	6.13E-03	-	hypothetical protein
2638	-1.09	1.49E-06	-	tRNA-dihydrouridine synthase
2725	-1.09	4.45E-07	-	ABC transporter ATP-binding protein/permease
378	-1.08	1.28E-03	-	transposase
2847	-1.08	2.02E-03	-	metallophosphoesterase
2275	-1.08	3.86E-06	-	beta-lactamase
884	-1.08	2.59E-03	-	ISEnfa3, transposase
827	-1.08	1.06E-06	-	PTS system lactose/cellobiose-specific transporter subunit IIC
2958	-1.08	1.53E-06	-	Nucleotidyl transferase of unknown function (DUF1814)
3044	-1.07	9.41E-07	-	mannosyl-glycoprotein endo-beta-N-acetylglucosaminidase
1304	-1.07	4.60E-05	-	ABC transporter ATP-binding protein
2918	-1.07	1.85E-04	-	PTS system transporter subunit IIBC
2306	-1.07	1.66E-05	*mta*	MerR family transcriptional regulator,Multidrug transporter activation protein
2924	-1.07	5.28E-05	-	transposase family protein
964	-1.07	6.05E-07	-	excinuclease ABC
2349	-1.07	1.80E-07	-	L-serine dehydratase iron-sulfur-dependent subunit alpha
1350	-1.07	1.29E-04	*xerD2*	phage integrase,Tyrosine recombinase XerD
1829	-1.07	5.86E-04	-	Integrase core domain protein
1364	-1.06	3.32E-06	-	putative methylase,Ribosomal RNA large subunit methyltransferase L
2773	-1.06	2.53E-06	*purR3*	LacI family transcriptional regulator
2119	-1.06	7.05E-03	*ycjP4*	ABC transporter permease
1249	-1.06	1.97E-06	-	ATP-dependent DNA helicase RecQ

1649	-1.06	1.02E-06	-	signal peptide peptidase SppA
2902	-1.05	4.94E-03	-	ISEnfa3, transposase,Transposase and inactivated derivatives,Transposase
763	-1.05	5.09E-05	-	M42 glutamyl aminopeptidase
413	-1.05	1.08E-05	*gltC1*	LysR family transcriptional regulator
2956	-1.05	8.44E-07	*ssb2*	single-strand binding protein
1769	-1.05	7.25E-04	-	transposase
2203	-1.05	4.10E-05	-	PTS system mannose/fructose/sorbose-specific IID component
1578	-1.05	4.41E-06	-	diacylglycerol kinase catalytic subunit
1562	-1.04	1.16E-02	-	glucitol operon activator protein,DNA-binding transcriptional activator GutM,Glucitol operon activator,Glucitol operon activator protein (GutM)
638	-1.04	1.61E-04	-	hypothetical protein
2570	-1.04	5.11E-06	-	DNA-directed RNA polymerase subunit omega
1420	-1.04	1.12E-05	-	BglG family transcriptional antiterminator,Ascorbate-specific phosphotransferase enzyme IIA component
2058	-1.04	1.21E-03	-	NUDIX family hydrolase,NADH pyrophosphatase
1823	-1.04	1.91E-06	*scrR*	sucrose operon repressor ScrR,Degradation activator,DNA-binding transcriptional repressor PurR,ABC-type sugar transport system, periplasmic component,catabolite control protein A,Periplasmic binding proteins and sugar binding domain of LacI family
1923	-1.04	1.63E-02	-	VanZ-like protein,Predicted integral membrane protein
751	-1.04	4.84E-05	*yabA*	DNA replication initiation control protein YabA
1220	-1.04	4.27E-07	-	adenylosuccinate lyase
2768	-1.03	6.90E-03	-	oxidoreductase zinc-binding dehydrogenase family protein
2427	-1.03	1.20E-03	-	HAD-superfamily hydrolase,Uncharacterized phosphatase YwpJ
632	-1.03	7.95E-05	*licT2*	BglG family transcriptional antiterminator
2494	-1.03	4.94E-04	-	transposase
2219	-1.02	5.32E-07	*steT2*	APC family amino acid transporter,Serine/threonine exchanger SteT
1646	-1.02	2.88E-07	-	peptidyl-prolyl cis-trans isomerase
2485	-1.02	2.51E-07	-	leucyl-tRNA synthetase
1652	-1.01	4.09E-07	-	glucosamine-fructose-6-phosphate aminotransferase
764	-1.01	1.13E-05	-	integral membrane protein
1085	-1.01	9.23E-07	-	cardiolipin synthetase
994	-1.01	1.96E-06	-	HAD superfamily hydrolase,Phosphatase YidA
1196	-1.01	1.71E-02	*dcuR*	response regulator receiver domain-containing protein
2362	-1.01	1.68E-04	*vraR2*	LuxR family DNA-binding response regulator,Response regulator protein vraR
2929	-1.00	3.87E-06	-	fructokinase
1936	-1.00	5.47E-06	-	protein-tyrosine-phosphatase

*Locus_tag: identifier corresponding to reference genome sequence, prefix to all numbers listed is "RBWH1_", **logFC: log_2 fold change, ***FDR: false discovery rate.
