# Supplementary material for: Chemical Synergy between Ionophore PBT2 and Zinc Reverses Antibiotic Resistance
Source: mBio. 2018 Dec 11;9(6):e02391-18. doi: 10.1128/mBio.02391-18 (PMC6299484; doi:10.1128/mBio.02391-18)
Supplement: TABLE S3 [file mbo006184211st3.pdf]

Supplementary Table 3. Real-time PCR primers used in this study.

**GAS**		
Gene	Primer direction	Primer sequence 5'->3'
proS	Fwd	AGCTGATCTCTGGCGTGAAT
proS	Rev	GGGTACGAAGCAAGCCATTA
mtsA	Fwd	CAATCGGTCAAGACCCTCAT
mtsA	Rev	CCATCAGACACGGCAAAGTA
czcD	Fwd	ATTGCACTTTGCACCATGAA
czcD	Rev	ACCATCCATCGACCAAACAT
glnA	Fwd	TGGATCAGGGATGCACTGTA
glnA	Rev	CCCAAGCGACATAAACAGGT
copA	Fwd	TCGAAGCTTTGCATCAACTG
copA	Rev	GAGCGGAGGTCTGCTATCAC
dacC	Fwd	AACACGCCAGCTTATGCTCT
dacC	Rev	CCTGATGCTGCCACAAGTAA
AdcB	Fwd	ATGGCGGTAGTTGCCATTAG
AdcB	Rev	CAAAATCGCCGTTGAAATCT
mgA	Fwd	CTGCCGTCTACGACAACAAA
mgA	Rev	CCCGTTGGTGAGTCTTGTTT
**MRSA**		
Gene	Primer direction	Primer sequence 5'->3'
rrsA	Fwd	GAAAGCCACGGCTAACTACG
rrsA	Rev	CATTTCACCGCTACACATGG
znuC	Fwd	CCGTTTGTCGGAATTGATTT
znuC	Rev	TGCTCCTTTGACTAGGGTCAC
zntA	Fwd	CGGTGTAAATGATGCACCTG
zntA	Rev	TAGCCGAATGCCCAAAATAG
copZ	Fwd	GAGCTGTGGTCACTGCAAAA
copZ	Rev	CCTTGATCTTCAATTGCGTCT
sek	Fwd	CATTTATGGACATAACGGCACT
sek	Rev	TTGGTAACCCATCATCTCCTG
glnA	Fwd	AAAATGCACGCGGATTTACT
glnA	Rev	GGGTTTGCAGCTGGATCTAC
frmA	Fwd	TTGGGGATATCAGGTTTTGC
frmA	Rev	TCCCGCTAGTTTAGCTCCAA
czcD	Fwd	GTTCAGTTGGCGCCATTACT
czcD	Rev	ACATGGCAATCATGCACACT
**VRE**		
Gene	Primer direction	Primer sequence 5'->3'
23S	Fwd	CTGCATTCCTTAGCCTCCTG
23S	Rev	CTAAGGTTTCCTGGGGAAGG
glnA	Fwd	CCGTTATTTGGGATCAATGG
glnA	Rev	TAGGCACGAGCATGTTTCAG
mntB_2	Fwd	CGACTGTCGCCGAAATAAAT

mntB_2	Rev	AAAAGCAATGGGGATGAATG
copA	Fwd	TCGGAACAAAAATCCCTGAG
copA	Rev	AAAAGAATCCGGATGACACG
hyl	Fwd	TGGGAAAGAGATGGAGATGG
hyl	Rev	AAATAGCTGGCATCGCTGTT
ssaB_2	Fwd	TCTTGGTATTAGCCGGTTGC
ssaB_2	Rev	ATCGCTTGCCTTTTTGATGT
zosA	Fwd	ATTGTGCTTCCTTCGTGTCC
zosA	Rev	CAGCCGCTTCTTTAGGTGTC
dps	Fwd	CCTGCAACAAGTGTTTCCAA
dps	Rev	TACATTGCACCCAATGATGG
